# Global, regional, and national burden of HIV/AIDS, 1990–2021, and forecasts to 2050, for 204 countries and territories: the Global Burden of Disease Study 2021

**DOI:** 10.1016/S2352-3018(24)00212-1

**Published:** 2024-11-25

**Authors:** GBD 2021 HIV Collaborators, Austin Carter, Meixin Zhang, Khai Hoan Tram, Magdalene K Walters, Deepa Jahagirdar, Edmond D Brewer, Amanda Novotney, Dylan Lasher, Emmanuel A Mpolya, Avina Vongpradith, Jianing Ma, Megan Verma, Tahvi D Frank, Jiawei He, Sam Byrne, Christine Lin, Regina-Mae Villanueva Dominguez, Spencer A Pease, Haley Comfort, Erin A May, Yohannes Habtegiorgis Abate, Hedayat Abbastabar, Atef Abdelkader, Parsa Abdi, Meriem Abdoun, Jeza Muhamad Abdul Aziz, Hassan Abidi, Olumide Abiodun, Richard Gyan Aboagye, Lucas Guimarães Abreu, Yonas Derso Abtew, Eman Abu-Gharbieh, Salahdein Aburuz, Ahmed Abu-Zaid, Isaac Yeboah Addo, Oyelola A Adegboye, Victor Adekanmbi, Charles Oluwaseun Adetunji, Juliana Bunmi Adetunji, Daniel Adedayo Adeyinka, Kishor Adhikari, Qorinah Estiningtyas Sakilah Adnani, Leticia Akua Adzigbli, Fatemeh Afrashteh, Saira Afzal, Shahin Aghamiri, Feleke Doyore Agide, Antonella Agodi, Williams Agyemang-Duah, Bright Opoku Ahinkorah, Faisal Ahmad, Sajjad Ahmad, Shahzaib Ahmad, Aqeel Ahmad, Ibrar Ahmed, Haroon Ahmed, Syed Anees Ahmed, Safoora Ahmed, Ali Ahmed, Mohammed Ahmed, Ayman Ahmed, Gizachew Taddesse Akalu, Karolina Akinosoglou, Salah Al Awaidy, Hanadi Al Hamad, Amjad S Al Mosa, Omar Ali Mohammed Al Zaabi, Samer O Alalalmeh, Nazmul Alam, Noore Alam, Fahad Mashhour Alanezi, Daniel Shewaye Alayu, Mohammad T AlBataineh, Seyedeh Yasaman Alemohammad, Adel Ali Saeed Al-Gheethi, Syed Shujait Ali, Mohammed Usman Ali, Abid Ali, Liaqat Ali, Waad Ali, Akram Al-Ibraheem, Joseph Uy Almazan, Awais Altaf, Diala Altwalbeh, Nelson Alvis-Guzman, Walid Adnan Al-Zyoud, Reza Amani, Tewodros Getnet Amera, Edward Kwabena Ameyaw, Sohrab Amiri, Hubert Amu, Ganiyu Adeniyi Amusa, Abhishek Anil, Abdul-Azeez Adeyemi Anjorin, Carl Abelardo T Antonio, Saleha Anwar, Razique Anwer, Ekenedilichukwu Emmanuel Anyabolo, Anayochukwu Edward Anyasodor, Geminn Louis Carace Apostol, Ali Ardekani, er Areda, Brhane Berhe Aregawi, Abdulfatai Aremu, Keivan Armani, Mulusew A Asemahagn, Mubarek Yesse Ashemo, Tahira Ashraf, Marvellous O Asika, Haftu Asmerom Asmerom, Maha Moh'd Wahbi Atout, Avinash Aujayeb, Hamzeh Awad, Adedapo Wasiu Awotidebe, Beatriz Paulina Ayala Quintanilla, Firayad Ayele, Sina Azadnajafabad, Shahkaar Aziz, Darshan B B, Giridhara Rathnaiah Babu, Muhammad Badar, Saeed Bahramian, Abdulaziz T Bako, Wondu Feyisa Balcha, Kiran Bam, Biswajit Banik, Mainak Bardhan, Till Winfried Bärnighausen, Hiba Jawdat Barqawi, Zarrin Basharat, Hameed Akande Bashiru, Afisu Basiru, Mohammad-Mahdi Bastan, Saurav Basu, Prapthi Persis Bathini, Kavita Batra, Ravi Batra, Nebiyou Simegnew Bayleyegn, Tahmina Begum, Amir Hossein Behnoush, Maryam Beiranvand, Melaku Ashagrie Belete, Abel Cherkos Belete, Apostolos Beloukas, Alice A Beneke, Azizullah Beran, Alemshet Yirga Berhie, Amiel Nazer C Bermudez, Robert S Bernstein, Kebede A Beyene, Pankaj Bhardwaj, Nikha Bhardwaj, Ajay Nagesh Bhat, Vivek Bhat, Gurjit Kaur Bhatti, Jasvinder Singh Singh Bhatti, Keralem Anteneh Bishaw, Khushboo D Bisht, Trupti Bodhare, Aadam Olalekan Bodunrin, Azizbek A Boltaev, Hamed Borhany, Souad Bouaoud, Colin Stewart Brown, Danilo Buonsenso, Katrin Burkart, Yasser Bustanji, Zahid A Butt, Chao Cao, Rosario Cárdenas, Muthia Cenderadewi, Joshua Chadwick, Chiranjib Chakraborty, Sandip Chakraborty, Rama Mohan Chandika, Vijay Kumar Chattu, Akhilanand Chaurasia, Guangjin Chen, Patrick R Ching, Hitesh Chopra, Sonali Gajanan Choudhari, Dinh-Toi Chu, Isaac Sunday Chukwu, Eric Chung, Zinhle Cindi, Rosa A S Couto, Natalia Cruz-Martins, Silvia Magali Cuadra-Hernández, Bashir Dabo, Omid Dadras, Gizachew Worku Dagnew, Tukur Dahiru, Xiaochen Dai, Aso Mohammad Darwesh, José das Neves, Nihar Ranjan Dash, Mohsen Dashti, Fernando Pio De la Hoz, Shayom Debopadhaya, Louisa Degenhardt, Ivan Delgado-Enciso, Kebede Deribe, Don C Des Jarlais, Hardik Dineshbhai Desai, Keshab Deuba, Amol S Dhane, Sameer Dhingra, Daniel Diaz, Michael R Diaz, Delaney D Ding, Thanh Chi Do, Sushil Dohare, Deepa Dongarwar, Wendel Mombaque dos Santos, Ojas Prakashbhai Doshi, Ashel Chelsea Dsouza, Haneil Larson Dsouza, Viola Savy Dsouza, Senbagam Duraisamy, Arkadiusz Marian Dziedzic, Alireza Ebrahimi, Abdelaziz Ed-Dra, Hisham Atan Edinur, Ferry Efendi, Michael Ekholuenetale, Temitope Cyrus Ekundayo, Iman El Sayed, Muhammed Elhadi, Chadi Eltaha, Sharareh Eskandarieh, Majid Eslami, Ugochukwu Anthony Eze, Ayesha Fahim, Ali Fatehizadeh, Nelsensius Klau Fauk, Patrick Fazeli, Ginenus Fekadu, Nuno Ferreira, Belete Sewasew Firew, Florian Fischer, Morenike Oluwatoyin Folayan, Behzad Foroutan, Takeshi Fukumoto, Sridevi G, Muktar A Gadanya, Abhay Motiramji Gaidhane, Abduzhappar Gaipov, Aravind P Gandhi, Mohammad Arfat Ganiyani, Miglas Welay Gebregergis, Mesfin Gebrehiwot, Teferi Gebru Gebremeskel, Motuma Erena Getachew, Keyghobad Ghadiri, Afsaneh Ghasemzadeh, Ahmad Ghashghaee, Ehsan Gholami, Nasim Gholizadeh, Mahsa Ghorbani, Artyom Urievich Gil, Alem Abera Girmay, Mahaveer Golechha, Davide Golinelli, Alessandra C Goulart, Anmol Goyal, Mesay Dechasa Gudeta, Sapna Gupta, Bhawna Gupta, Awoke Derbie Habteyohannes, Dariush Haghmorad, Arvin Haj-Mirzaian, Rabih Halwani, Demelash Woldeyohannes Handiso, Zaim Anan Haq, Harapan Harapan, Arief Hargono, Ahmed I Hasaballah, Md Saquib Hasnain, Shoaib Hassan, Soheil Hassanipour, Omar E Hegazi, Mohammad Heidari, Kamal Hezam, Mbuzeleni Mbuzeleni Hlongwa, Nguyen Quoc Hoan, Praveen Hoogar, Mehdi Hosseinzadeh, Ahmad Hosseinzadeh Adli, Tsegaye Gebreyes Hundie, Kiavash Hushmandi, Hong-Han Huynh, Segun Emmanuel Ibitoye, Adalia Ikiroma, Kevin S Ikuta, Olayinka Stephen Ilesanmi, Irena M Ilic, Arnaud Iradukunda, Mustafa Alhaji Isa, Nahlah Elkudssiah Ismail, Ihoghosa Osamuyi Iyamu, Vinothini J, Kathryn H Jacobsen, Akhil Jain, Ammar Abdulrahman Jairoun, Mihajlo Jakovljevic, Manthan Dilipkumar Janodia, Amirreza Javadi Mamaghani, Alelign Tasew Jema, Mohammad Jokar, Jost B Jonas, Nitin Joseph, Charity Ehimwenma Joshua, Ali Kabir, Md. Awal Kabir, Zubair Kabir, Vidya Kadashetti, Feroze Kaliyadan, Kehinde Kazeem Kanmodi, Suthanthira Kannan S, Ibraheem M Karaye, Arman Karimi Behnagh, Molly B Kassel, Gbenga A Kayode, Himanshu Khajuria, Nauman Khalid, Anees Ahmed Khalil, Faham Khamesipour, Gulfaraz Khan, Ejaz Ahmad Khan, Yusra H Khan, Mohammad Jobair Khan, M Nuruzzaman Khan, Khaled Khatab, Feriha Fatima Khidri, Zahra Khorrami, Majid Khosravi, Jagdish Khubchandani, Min Seo Kim, Jong Yeob Kim, Yun Jin Kim, Adnan Kisa, Sezer Kisa, Somayeh Komaki, Shivakumar KM Marulasiddaiah Kondlahalli, Parvaiz A Koul, Sindhura Lakshmi Koulmane Laxminarayana, Kewal Krishan, Barthelemy Kuate Defo, Md Abdul Kuddus, Mukhtar Kulimbet, Vishnutheertha Kulkarni, Rakesh Kumar, Vijay Kumar, Nithin Kumar, Manasi Kumar, Muhammad Awwal Ladan, Dharmesh Kumar Lal, Thao Thi Thu Le, Nhi Huu Hanh Le, Seung Won Lee, Kate E LeGrand, Temesgen L Lerango, Ming-Chieh Li, Virendra S Ligade, Stephen S Lim, Liknaw Workie Limenh, Xuefeng Liu, Runben Liu, Rakesh Lodha, Arianna Maever Loreche, Hawraz Ibrahim M. Amin, Zheng Feei Ma, Azeem Majeed, Elaheh Malakan Rad, Hardeep Singh Malhotra, Kashish Malhotra, Ahmad Azam Malik, Iram Malik, Tauqeer Hussain Mallhi, Mohammad Ali Mansournia, Bishnu P Marasini, Bernardo Alfonso Martinez-Guerra, Francisco Rogerlândio Rogerlândio Martins-Melo, Miquel Martorell, Roy Rillera Marzo, Navgeet Mathur, Anna Laura W McKowen, Hadush Negash Meles, Endalkachew Belayneh Melese, Ziad Ahmed Memish, Walter Mendoza, Ritesh G Menezes, Tuomo J Meretoja, Tomislav Mestrovic, Peter Meylakhs, Laurette Mhlanga, Irmina Maria Michalek, Ana Carolina Micheletti Gomide Nogueira de Sá, Giuseppe Minervini, Le Huu Nhat Minh, Babak Moazen, Nouh Saad Mohamed, Sakineh Mohammad-Alizadeh-Charandabi, Abdollah Mohammadian-Hafshejani, Hussen Mohammed, Salahuddin Mohammed, Mustapha Mohammed, Ali H Mokdad, Lorenzo Monasta, Mohammad Ali Moni, Fateme Montazeri, Maryam Moradi, Yousef Moradi, Rohith Motappa, Vincent Mougin, Sumaira Mubarik, George Duke Mukoro, Francesk Mulita, Kavita Munjal, Yanjinlkham Munkhsaikhan, B.V. Murlimanju, Fungai Musaigwa, Ghulam Mustafa, Saravanan Muthupandian, Ahamarshan Jayaraman Nagarajan, Pirouz Naghavi, Gurudatta Naik, Firzan Nainu, Mohammad Sadeq Najafi, Shumaila Nargus, Samidi Nirasha Kumari Navaratna, Muhammad Naveed, Vinod C Nayak, Biswa Prakash Nayak, Sabina Onyinye Nduaguba, Chernet Tafere Negesse, Mohammad Hadi Nematollahi, Georges Nguefack-Tsague, Dang H Nguyen, Hien Quang Nguyen, Van Thanh Nguyen, Robina Khan Niazi, Yeshambel T Nigatu, Nasrin Nikravangolsefid, Vikram Niranjan, Chukwudi A Nnaji, Syed Toukir Ahmed Noor, Nawsherwan Not applicable, Jean Jacques Noubiap, Chisom Adaobi Nri-Ezedi, Fred Nugen, Jerry John Nutor, Chimezie Igwegbe Nzoputam, Ogochukwu Janet Nzoputam, Kehinde O Obamiro, Ismail A Odetokun, Onome Bright Oghenetega, Ayodipupo Sikiru Oguntade, Sylvester Reuben Okeke, Akinkunmi Paul Okekunle, Osaretin Christabel Okonji, Andrew T Olagunju, Babayemi Oluwaseun Olakunde, Oladotun Victor Olalusi, Matthew Idowu Olatubi, Abdulhakeem Abayomi Olorukooba, Isaac Iyinoluwa Olufadewa, Ahmed Omar Bali, Obinna E Onwujekwe, Abdulahi Opejin, Michal Ordak, Verner N Orish, Edgar Ortiz-Brizuela, Uchechukwu Levi Osuagwu, Amel Ouyahia, Mahesh Padukudru P A, Jagadish Rao Padubidri, Claudia Palladino, Ashok Pandey, Leonidas D Panos, Jose L Paredes, Pragyan Paramita Parija, Romil R Parikh, Ava Pashaei, Maja Pasovic, Sangram Kishor Patel, Aslam Ramjan Pathan, Shankargouda Patil, Shrikant Pawar, Veincent Christian Filipino Pepito, Emmanuel K Peprah, Prince Peprah, Marcos Pereira, Simone Perna, Ionela-Roxana Petcu, Hoang Tran Pham, Julian David Pillay, Ramesh Poluru, Maarten J Postma, Naeimeh Pourtaheri, Jalandhar Pradhan, Prem Prakash, Thejeswar N N Prakasham, Elton Junio Sady Prates, Dimas Ria Angga Pribadi, Tina Priscilla, Jagadeesh Puvvula, Ibrahim Qattea, Asma Saleem Qazi, Raghu Anekal Radhakrishnan, Quinn Rafferty, Ibrar Rafique, Fakher Rahim, Afarin Rahimi-Movaghar, Vafa Rahimi-Movaghar, Mosiur Rahman, Amir Masoud Rahmani, Shayan Rahmani, Nazanin Rahmanian, Mohammad Rahmanian, Vahid Rahmanian, Sathish Rajaa, Mahmoud Mohammed Ramadan, Hazem Ramadan, Shakthi Kumaran Ramasamy, Pushkal Sinduvadi Ramesh, Kritika Rana, Chhabi Lal Ranabhat, Mithun Rao, Sowmya J Rao, Mohammad-Mahdi Rashidi, Devarajan Rathish, Santosh Kumar Rauniyar, Salman Rawaf, Elrashdy Moustafa Mohamed Redwan, Robert C Reiner Jr., Mohsen Rezaeian, Jefferson Antonio Buendia Rodriguez, Kevin T Root, Allen Guy Ross, Kunle Rotimi, Nitai Roy, Godfrey M Rwegerera, Cameron John Sabet, Basema Ahmad Saddik, Mohammad Reza Saeb, Umar Saeed, Pooya Saeedi, Sher Zaman Zaman Safi, Rajesh Sagar, Fatemeh Saheb Sharif-Askari, Narjes Saheb Sharif-Askari, Amirhossein Sahebkar, Soumya Swaroop Sahoo, Zahra Saif, Mirza Rizwan Sajid, Nasir Salam, Afeez Abolarinwa Salami, Mohamed A Saleh, Leili Salehi, Hossein Samadi Kafil, Abdallah M Samy, Rama Krishna Sanjeev, Milena M Santric-Milicevic, Aswini Saravanan, Benn Sartorius, Anudeep Sathyanarayan, Maheswar Satpathy, Monika Sawhney, Mansour Sedighi, Birhan Ewunu Semagn, Sabyasachi Senapati, Yashendra Sethi, Allen Seylani, Pritik A Shah, Samiah Shahid, Masood Ali Shaikh, Ali Shamekh, Mohammad Ali Shamshirgaran, Anas Shamsi, Mohd Shanawaz, Mohammed Shannawaz, Amin Sharifan, Javad Sharifi-Rad, Shamee Shastry, Rekha Raghuveer Shenoy, Premalatha K Shetty, Mahabalesh Shetty, Pavanchand H Shetty, Desalegn Shiferaw, Reza Shirkoohi, Aminu Shittu, Sunil Shrestha, Migbar Mekonnen Sibhat, Emmanuel Edwar Siddig, Mark J Siedner, Jasvinder A Singh, Paramdeep Singh, Surjit Singh, Harmanjit Singh, Robert Sinto, Anna Aleksandrovna Skryabina, Amanda E Smith, Farrukh Sobia, Anton Sokhan, Shipra Solanki, Ranjan Solanki, Reed J D Sorensen, Sahabi K Sulaiman, Lukasz Szarpak, Sree Sudha T Y, Mohammad Tabish, Santosh Kumar Tadakamadla, Yasaman Taheri Abkenar, Jabeen Taiba, Iman M Talaat, Mircea Tampa, Jacques Lukenze Tamuzi, Ker-Kan Tan, Manoj Tanwar, Elvis Enowbeyang Tarkang, Nuno Taveira, Gebrehiwot Teklay, Behailu Terefe Tesfaye, Enoch Teye-Kwadjo, Ramna Thakur, Pugazhenthan Thangaraju, Rajshree Thapa, Rekha Thapar, Friedrich Thienemann, Joe Thomas, Marcos Roberto Tovani-Palone, Thang Huu Tran, Mai Thi Ngoc Tran, Alexander C Tsai, Guesh Mebrahtom Tsegay, Munkhtuya Tumurkhuu, Arit Udoh, Irfan Ullah, Atta Ullah, Muhammad Umair, Muhammad Umar, Bhaskaran Unnikrishnan, Sanaz Vahdati, Asokan Govindaraj Vaithinathan, Shoban Babu Varthya, Tommi Juhani Vasankari, Georgios-Ioannis Verras, Jorge Hugo Villafañe, Anh Truc Vo, Theo Vos, Mandaras Tariku Walde, Richard G Wamai, Yanzhong Wang, Muhammad Waqas, Paul Ward, Gizachew Tadesse Wassie, Robert G Weintraub, Haftom Legese Weldetinsaa, Gebre Adhanom Weldu, Ronny Westerman, Nuwan Darshana Wickramasinghe, Mesfin Agachew Woldekidan, Yen Jun Wong, Nigus Kassie Worku, Zenghong Wu, Xinsheng Wu, Sajad Yaghoubi, Gesila Endashaw Yesera, Saber Yezli, Siyan Yi, Arzu Yiğit, Dehui Yin, Yazachew Yismaw, Dong Keon Yon, Naohiro Yonemoto, Fathiah Zakham, Haijun Zhang, Jingya Zhang, Hanqing Zhao, Bin Zhu, Qingyuan Zhuang, Abzal Zhumagaliuly, Magdalena Zielińska, Liu Zihao, Yossef Teshome Zikarg, Mohammad Zoladl, Alimuddin Zumla, Samer H Zyoud, Peng Zheng, Aleksandr Y Aravkin, Jeffrey W Imai-Eaton, Mohsen Naghavi, Austin E Schumacher, Simon I Hay, Christopher J L Murray, Hmwe Kyu

## Abstract

**Background:**

As set out in Sustainable Development Goal 3.3, the target date for ending the HIV epidemic as a public health threat is 2030. Therefore, there is a crucial need to evaluate current epidemiological trends and monitor global progress towards HIV incidence and mortality reduction goals. In this analysis, we assess the current burden of HIV in 204 countries and territories and forecast HIV incidence, prevalence, and mortality up to 2050 to allow countries to plan for a sustained response with an increasing number of people living with HIV globally.

**Methods:**

We used the Global Burden of Diseases, Injuries, and Risk Factors Study (GBD) 2021 analytical framework to compute age-sex-specific HIV mortality, incidence, and prevalence estimates for 204 countries and territories (1990–2021). We aimed to analyse all available data sources, including data on the provision of HIV programmes reported to UNAIDS, published literature on mortality among people on antiretroviral therapy (ART) identified by a systematic review, household surveys, sentinel surveillance antenatal care clinic data, vital registration data, and country-level case report data. We calibrated a mechanistic simulation of HIV infection and natural history to available data to estimate HIV burden from 1990 to 2021 and generated forecasts to 2050 through projection of all simulation inputs into the future. Historical outcomes (1990–2021) were simulated at the 1000-draw level to support propagation of uncertainty and reporting of uncertainty intervals (UIs). Our approach to forecasting utilised the transmission rate as the basis for projection, along with new rate-of-change projections of ART coverage. Additionally, we introduced two new metrics to our reporting: prevalence of unsuppressed viraemia (PUV), which represents the proportion of the population without a suppressed level of HIV (viral load <1000 copies per mL), and period lifetime probability of HIV acquisition, which quantifies the hypothetical probability of acquiring HIV for a synthetic cohort, a simulated population that is aged from birth to death through the set of age-specific incidence rates of a given time period.

**Findings:**

Global new HIV infections decreased by 21·9% (95% UI 13·1–28·8) between 2010 and 2021, from 2·11 million (2·02–2·25) in 2010 to 1·65 million (1·48–1·82) in 2021. HIV-related deaths decreased by 39·7% (33·7–44·5), from 1·19 million (1·07–1·37) in 2010 to 718 000 (669 000–785 000) in 2021. The largest declines in both HIV incidence and mortality were in sub-Saharan Africa and south Asia. However, super-regions including central Europe, eastern Europe, and central Asia, and north Africa and the Middle East experienced increasing HIV incidence and mortality rates. The number of people living with HIV reached 40·0 million (38·0–42·4) in 2021, an increase from 29·5 million (28·1–31·0) in 2010. The lifetime probability of HIV acquisition remains highest in the sub-Saharan Africa super-region, where it declined from its 1995 peak of 21·8% (20·1–24·2) to 8·7% (7·5–10·7) in 2021. Four of the seven GBD super-regions had a lifetime probability of less than 1% in 2021. In 2021, sub-Saharan Africa had the highest PUV of 999·9 (857·4–1154·2) per 100 000 population, but this was a 64·5% (58·8–69·4) reduction in PUV from 2003 to 2021. In the same period, PUV increased in central Europe, eastern Europe, and central Asia by 116·1% (8·0–218·2). Our forecasts predict a continued global decline in HIV incidence and mortality, with the number of people living with HIV peaking at 44·4 million (40·7–49·8) by 2039, followed by a gradual decrease. In 2025, we projected 1·43 million (1·29–1·59) new HIV infections and 615 000 (567 000–680 000) HIV-related deaths, suggesting that the interim 2025 targets for reducing these figures are unlikely to be achieved. Furthermore, our forecasted results indicate that few countries will meet the 2030 target for reducing HIV incidence and HIV-related deaths by 90% from 2010 levels.

**Interpretation:**

Our forecasts indicate that continuation of current levels of HIV control are not likely to attain ambitious incidence and mortality reduction targets by 2030, and more than 40 million people globally will continue to require lifelong ART for decades into the future. The global community will need to show sustained and substantive efforts to make the progress needed to reach and sustain the end of AIDS as a public threat.

**Funding:**

The Bill & Melinda Gates Foundation and the National Institute of Allergy and Infectious Diseases.

**Research in context**

**Evidence before this study**

The global burden of HIV/AIDS has been estimated by the Global Burden of Diseases, Injuries, and Risk Factors Study (GBD) and UNAIDS in previous reports. GBD 2019 assessed sex-specific HIV burden and evaluated progress towards global targets using established metrics, including incidence-to-mortality ratio and incidence-to-prevalence ratio, in 204 countries and territories. Because existing metrics might not fully capture the nuanced risks at the population level, novel metrics could deepen our understanding of the HIV epidemic. These include metrics such as the prevalence of unsuppressed viraemia (PUV) and the period lifetime probability of acquiring HIV. We searched PubMed for the terms (“HIV” OR “HIV”[Mesh]) AND (“burden” OR “estimate*” OR “forecast*” OR “forecasting”[Mesh]) AND (“unsuppressed viremia” OR “unsuppressed viraemia” OR “lifetime probability*” OR “lifetime risk*”) AND (“1980/01/01”[PDAT] : “2024/04/29”[PDAT]), with no language restrictions, for publications up to April 29, 2024. This search yielded 52 studies. Among these, three studies focused on the population-level PUV in a single country, and five studies addressed the lifetime risk of HIV infection for a representative population within a single country. However, our search did not identify any studies that reported on either or both of these metrics at a global level across countries.

**Added value of this study**

To our knowledge, we provide the first global estimates of the period lifetime probability of HIV acquisition and PUV. These additional metrics enable comparisons between countries and over time, which succinctly characterise the probability of infection and the effectiveness of HIV control in limiting the amount of HIV circulating in a population. In addition, the combination of historical estimates and forecasts enables our study to provide commentary on progress made and the implications of continuation of recent trends. Our forecasting methods used an enhanced approach to capture the expected projection of past trends and relationships by using the transmission rate instead of the confounded incidence hazard as the driver of future transmission. Updated data from more recent time periods improved our estimates of recent trends in burden and assisted with ensuring that our forecasts reflect current trends. Finally, country-level forecasts out to 2050 assist with long-term strategic planning and inform policy investments.

**Implications of all the available evidence**

Our study expands on previous efforts to characterise the state of the HIV epidemic and project the consequences of continuation of recent trends in the level of HIV control. With few countries projected to achieve the UNAIDS 2030 targets for reductions in both HIV incidence and mortality, our analysis forecasts estimated timelines for individual countries to reach these targets at current rates. Progress in achieving substantial reductions in the burden of HIV is most evident in sub-Saharan Africa, whereas worrisome trends emerge in central Europe, eastern Europe, and central Asia, and north Africa and the Middle East. The inclusion of additional HIV-specific metrics in our HIV burden reporting enables new perspectives on the current epidemic status at the population level and suggests possible thresholds for future target setting. Sustaining and strengthening the global HIV response into the future will be crucial with an increasing number of people living with HIV in every region. These estimates could assist policy makers in planning a pathway towards ending AIDS as a public health threat globally up to 2030 and beyond.

## Introduction

Four decades since the first reported cases of AIDS, immense scientific advances and greatly expanded access to life-saving antiretroviral therapy (ART), effective HIV prevention options, and innovative care models^1^ have substantially reduced both new HIV infections and HIV-related mortality^2^—gains made possible by global coordinated efforts towards ending the AIDS epidemic.^3^, ^4^, ^5^ Despite considerable progress over this time, more than 1 million people newly acquire HIV infection each year, and HIV continues to be a major cause of mortality in many settings.^2^, ^6^ Moreover, of the estimated 40 million people living with HIV, only about three-quarters are currently on treatment,^6^ further underscoring the need to improve access and utilisation of care around the globe.^7^

In 2016, the international community reaffirmed its commitment to ending the AIDS epidemic as a public health threat by 2030, establishing this objective as one of the UN Sustainable Development Goals^7^ and adopting the UNAIDS Fast-Track strategy with interim targets—the original 90-90-90 testing and treatment targets—to be achieved by 2020.^8^ Although substantial progress was made, few countries were able to meet these intermediate targets by 2020,^9^ and in recent years the global community has had to grapple with the enormous challenges posed by the COVID-19 pandemic. Surprisingly, the impact of COVID-19 on HIV programmes was smaller than expected, with many countries able to successfully continue delivery of ART and other HIV services during the pandemic with limited interruption.^10^, ^11^, ^12^, ^13^ In 2021, the UN^14^ set out a new global strategy with a focus on inequalities, establishing new interim targets for 2025 on the path towards 2030.^15^ Broadly, these 2025 targets aim to reduce the number of new HIV infections to fewer than 370 000; reduce the number of AIDS-related deaths to fewer than 250 000; ensure 34 million people living with HIV are on treatment; and achieve 95-95-95 for all groups and in all settings (95% of people living with HIV diagnosed, of whom 95% are on treatment, of whom 95% are virally suppressed).^15^

With ambitious targets and substantial resources at our disposal, it is crucial to measure progress and identify areas needing improvement. Assessing current levels and predicting future trends in HIV incidence and mortality can help determine progress, but these metrics might not give a complete picture of the underlying risks posed at the population level. Two metrics with the potential to enhance our understanding of the HIV epidemic are population-level prevalence of unsuppressed viraemia (PUV)^16^, ^17^, ^18^ and period lifetime probability of HIV acquisition.^19^, ^20^, ^21^ PUV indicates the proportion of the total population without suppressed viral loads (viral loads <1000 copies per mL), reflecting how both increasing viral suppression among people living with HIV and decreasing prevalence from prevention of new infections shape population-level risk of HIV transmission. Period lifetime probability of HIV acquisition characterises cross-sectional, age-specific incidence rates with a single number that indicates the proportion of the population that would acquire HIV if it were to age through period age-specific incidence rates. This summary of incidence communicates the lifelong nature of HIV infection probability in terms that are more interpretable than annualised incidence rates.

Analyses to systematically assess global trends in HIV epidemiology are produced at regular intervals by Global Burden of Diseases, Injuries, and Risk Factors Study (GBD) collaborators, UNAIDS, and other modelling groups, several of whom contribute to the work of the UNAIDS Reference Group on Estimates, Modelling and Projections.^22^ At the national level, country teams have conducted national HIV prevalence surveys to inform programmatic activities.^23^ Although clearly important for understanding and responding to local epidemics, these national surveys and modelling estimates provide only a limited view of the global HIV epidemic and regional trends. The GBD approach provides a comprehensive perspective on global and regional trends by synthesising myriad datasets to generate internally consistent and comparable modelling estimates and situating HIV in the context of other disease burdens and health challenges.

This study leverages updated GBD estimates and forecasts to track progress against HIV globally, with additional metrics alongside previously reported epidemiological rates. Specifically, we used results from GBD 2021 with forecasts to 2050 to systematically assess trends in HIV burden in 204 countries and territories and across seven GBD super-regions from 1990 to 2021, as well as to provide country-specific forecasts of HIV incidence, prevalence, and mortality from 2022 to 2050.

## Methods

### Overview

This manuscript was produced as part of the GBD Collaborator Network and in accordance with the GBD protocol. Compared with our previous iteration,^2^ GBD 2021 provided annual estimates from 1990 to 2021 for 204 countries and territories, two sexes, and ages 0 years to 95 years and older. For comparison, we highlight the relative change in burden over various time periods, including declines relative to the year in which a particular metric peaked globally. The conceptual and analytical framework for the GBD, its hierarchy of causes, and detailed methods have been published elsewhere.^24^ Here, we describe the specific methods used in GBD 2021 for analysing the burden of HIV. The study complies with the GATHER statement;^25^ data and code for GBD 2021 HIV estimation process are available online. GBD results are stratified by seven super-regions, defined as follows: central Europe, eastern Europe, and central Asia; high income; Latin America and the Caribbean; north Africa and the Middle East; south Asia; southeast Asia, east Asia, and Oceania; and sub-Saharan Africa (appendix 1 p 4).

### Input data and modelling strategy

We analysed all available data sources, including data on the provision of HIV programmes reported to UNAIDS, published on-ART mortality literature identified by a systematic review, household surveys, sentinel surveillance antenatal care clinic data, vital registration data, and country-level case report data. We grouped countries and territories and applied differentiated modelling strategies according to the types of data available to estimate HIV trends. Group 1 included countries and territories with HIV prevalence data from antenatal care clinics or representative population-based seroprevalence surveys (51 countries in total, including most in sub-Saharan Africa and the Dominican Republic, Haiti, India, and Papua New Guinea). Group 2 included the remaining 153 countries, of which 120 had data on HIV deaths. 33 countries had no data on HIV-related deaths. The groups were further stratified based on peak prevalence with or without vital registration data completeness (appendix 1 p 3).

Group 1 locations were modelled with the Estimation and Projection Package Age-Sex Model (EPP-ASM), a discrete time compartmental model of adult HIV stratified by single-year age, sex, disease stage, and treatment status.^2^, ^23^ In EPP-ASM, the transmission rate, r(t), is applied at each time step (one-tenth of a year) to the susceptible and infectious populations. r represents the number of new cases expected to emanate per year from an adult aged 15–49 years with untreated HIV infection. Group 2 locations were modelled by estimating exogenous HIV incidence trend inputs to our GBD recoded implementation of Spectrum,^26^ which is also a discrete time compartmental model stratified by single-year age, sex, disease, and treatment status. The EPP-ASM and Spectrum models used for GBD estimation vary slightly from those used by UNAIDS, with differences between our estimates and UNAIDS' estimates reflecting differences in model structure, model parameters, and the location-specific data used to calibrate our models.

In addition to our previously described augmentations to EPP-ASM for GBD purposes,^2^ we further refined the use of EPP-ASM in this iteration of the GBD. For India, we used EPP-ASM in combination with Spectrum to calibrate estimates to age-disaggregated and sex-disaggregated prevalence surveys and Sample Registration System data. The Sample Registration System data were used to inform the age and sex distribution of incidence. Additionally, we modified the prior distribution for the transmission rate for India to better reflect the comparatively lower magnitude of the epidemic as compared with sub-Saharan African countries for which EPP-ASM parameter prior distributions were initially developed.^27^ Country-specific data on intervention coverage reported to UNAIDS, such as ART and prevention of mother-to-child transmission, were used across all locations, regardless of the selected modelling strategy.^28^ Similarly, all countries and territories relied on rates of disease progression and HIV-free mortality (ie, the expected background mortality not caused by HIV). Countries and territories in group 1, utilising EPP-ASM, leveraged population-representative HIV seroprevalence surveys and antenatal care data. All antenatal care data are adjusted to be population representative. Adjusted vital registration data were used for group 2 countries and territories (appendix 1 p 4).

### Statistical analysis and uncertainty estimation

EPP-ASM is calibrated using incremental mixture importance sampling, where draws of the transmission rate are generated and simulated outputs are compared with observed prevalence data to determine parameter likelihood. In GBD 2021, a binomial likelihood was used in place of the normal likelihood. This method improved the fit to the zero-proportion data while minimally affecting fits in non-zero prevalence age-sex strata. South Africa, India, Kenya, the Gambia, Niger, Burundi, Ethiopia, Rwanda, Ghana, São Tomé and Príncipe, Senegal, and Sierra Leone were affected by this change. For GBD 2021, we ran Spectrum on every combination of incidence and treatment options and determined the root mean squared error of the resulting mortality relative to the vital registration data.

On-ART mortality rates were modelled separately from off-ART mortality and disease progression rates, which we estimated jointly. 1000 draws were sampled from the posterior distributions of these transition parameter models. Additionally, we drew treatment input values from a uniform distribution around the reported level of treatment on each draw to ensure adequate uncertainty was captured. We then fit our HIV simulation to data 1000 times, each with a separate set of input draw-level transition parameters, and then sampled a single draw from each fit to create 1000 draws from which 95% uncertainty intervals (UIs) were derived, with the mean of all draws as the point estimate and the 2·5th and 97·5th percentile draws as the upper and lower bounds. In this analysis, we calculated percentage change as the difference between the final year value and the initial year value, then divided by the initial value, and multiplied by 100.

### HIV forecasting

We forecasted HIV incidence, prevalence, mortality, and treatment coverage from 2022 through to 2050 in Spectrum using input parameters extended to 2050. Our forecasts were driven by extrapolation of past trends and relationships in (1) the rate of transmission in the absence of treatment and (2) ART coverage. Changes in the probability of transmission in the absence of treatment implicitly reflect all changes in transmission reduction separate from ART, which would include behaviour change and increasing coverage of prevention interventions. In this round of estimation, we forecasted the HIV transmission rate per untreated person living with HIV, which differs from the previous round of estimation, in which we projected the incidence rate. The transmission rate interacts with prevalence dynamically and is anticipated to be stable under unaltered conditions. Forecasting the transmission rate involved two steps. We calculated a global median transmission rate by taking the population-weighted median transmission rate across the preceding decade (2011–21). Then, we generated location-specific projections of the logit transmission rate using the observed logit transmission rate within the preceding 5-year timeframe (2016–21). We combined these two projections by smoothly transitioning from the location-specific projection to the global equilibrium over the future 20-year period (2021–41). We implemented a conservative approach to prevent inducing rises in incidence; if the location-specific projection already indicated a lower transmission rate than the global equilibrium, we refrained from adjusting it upwards. Furthermore, we capped the location-specific trends at a 25% increase relative to the last observed value. We adopted an optimistic approach to forecasting ART coverage, using the largest rate of change for each age-sex combination along with a cap of 95% ART coverage within each stratum. This approach resulted in ART coverage increases across the board and prevented exponentially increasing HIV epidemics in the future. Full details on the methods for forecasting are provided in appendix 1 (pp 14–16).

### Additional metrics

The metric of period lifetime probability of HIV acquisition characterises period age-specific incidence rates with a single number approximating the hypothetical probability of acquiring HIV for a population experiencing these incidence rates throughout their lifetimes. Period lifetime probability operates very similarly to period life expectancy at birth, in that it summarises the rates of a particular time period in contrast to the cohort lifetime probability or cohort life expectancy, which is calculated using the actual changing rates experienced by a cohort over their lifetimes. To contextualise values of this period lifetime probability, we use a 1% threshold as an achievable benchmark that can serve as a target for locations that currently exceed a 1% period lifetime probability of HIV.

In practice, we calculated period lifetime probability of HIV acquisition by simulating a cohort ageing through every age group, removing the proportion of the cohort that die from non-HIV causes and removing the proportion that acquire HIV. We iterated through each age group, converting incidence and background mortality rates to probabilities following a competing risk framework^29^ and removing them from the susceptible group. After iterating through all age groups, the entire synthetic cohort was sorted into those who died before acquiring HIV and those who acquired HIV. We repeated this calculation for all time periods in every location to generate location-specific time series of lifetime probability of HIV acquisition.

PUV was calculated by combining our prevalence estimates with UNAIDS estimates of the proportion of people living with HIV who are virally suppressed.^30^ We produced estimates of PUV among the total population in all ages by multiplying the number of people living with HIV who are on ART by the proportion virally unsuppressed, adding on the number of people with HIV who are not on ART, and then dividing the sum by the total population to produce PUV among the total population. Further details on the calculation of the new metrics are provided in appendix 1 (p 16).

### Role of the funding source

The funders of the study had no role in study design, data collection, data analysis, data interpretation, or writing of the report.

## Results

**Figure 1:**
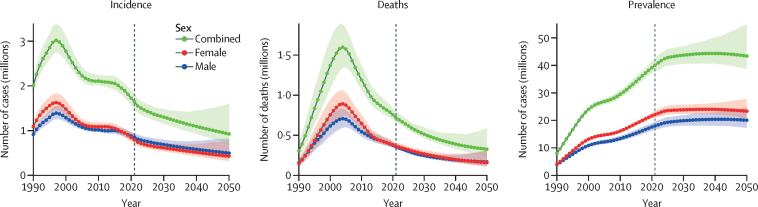
Temporal trends of HIV incidence, mortality, and prevalence counts for 1990–2050 Time trends presented for all-age males, females, and both sexes combined. The shaded areas represent 95% uncertainty intervals.

In 2021, an estimated 40·0 million (95% UI 38·0–42·4) people were living with HIV globally, of whom 18·0 million (16·6–19·4) were male and 22·1 million (21·2–23·2) were female (figure 1). The majority of people living with HIV resided in sub-Saharan Africa (29·1 million, 28·0–30·4); however, numbers of people living with HIV also increased in other GBD super-regions between 2010 and 2021, reaching the following levels in 2021: high-income countries with the most at 3·13 million (2·04–4·19); and north Africa and the Middle East with the least at 229 000 (143 000–407 000; appendix 2 p 2). Of people living with HIV, 71·8% (69·7–73·5) were on ART at the end of 2021 (appendix 2 p 1).

**Figure 2:**
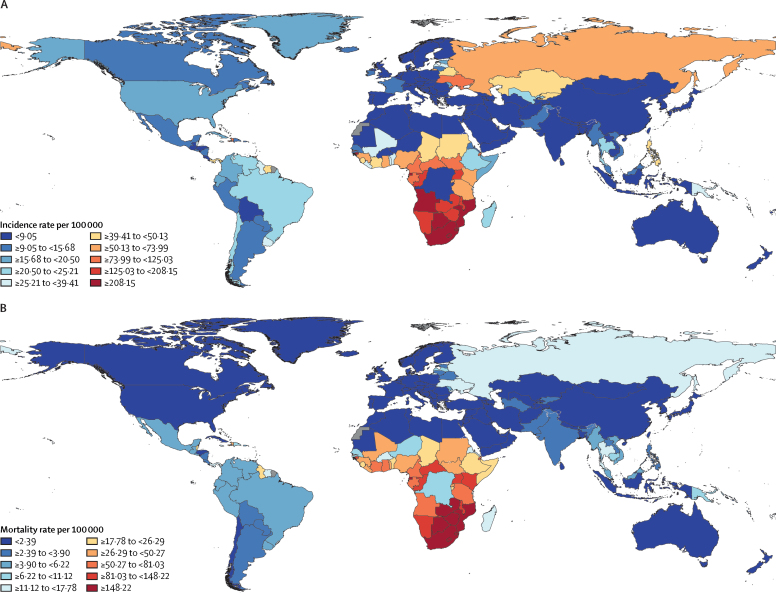
HIV incidence (A) and mortality (B) rates for both sexes combined in 2021, age-standardised Rates are presented per 100 000 individuals.

In 2021, 1·65 million (95% UI 1·48 to 1·82) people newly acquired HIV infection globally, including 852 000 males (766 000 to 944 000) and 794 000 females (703 000 to 901 000; figure 1), a reduction from 2·11 million (2·02 to 2·25) global new infections in 2010. This overall decline in HIV incidence of 21·9% (13·1 to 28·8) is largely driven by substantial reductions in new HIV infections in sub-Saharan Africa (table). The south Asia region also showed a reduction in the number of incident cases of 35·4% (0·1 to 56·5) during this time. However, new infections increased in other super-regions, most notably central Europe, eastern Europe, and central Asia (112·6% [77·8 to 145·8]) and north Africa and the Middle East (66·1% [–0·6 to 230·9]). The highest age-standardised incidence rates per 100 000 population in 2021 were in Lesotho, South Africa, and Eswatini, although these have declined since 2010 (figure 2A; appendix 2 p 7). Age-standardised incidence rates in other countries, such as Kazakhstan and Kyrgyzstan in central Asia, Belarus and Ukraine in eastern Europe, and Sudan continued to increase from 2010 to 2021 (figure 2A; appendix 2 p 7).

**Table:** HIV mortality, incidence, and prevalence counts and rates, with percentage change between 2010 and 2021, all ages and sexes combined, globally and in seven GBD super-regions

	**Mortality**						**Incidence**						**Prevalence**			
	2010 death count	2021 death count	Percentage change in death count 2010–21	2010 rate (per 100 000)	2021 rate (per 100 000)	Percentage change in rate 2010–21	2010 count	2021 count	Percentage change in count 2010–21	2010 rate (per 100 000)	2021 rate (per 100 000)	Percentage change in rate 2010–21	2010 count	2021 count	2010 rate (per 100 000)	2021 rate (per 100 000)
Global	1 190 000 (1 070 000 to 1 370 000)	718 000 (669 000 to 785 000)	−39·7 (−44·5 to −33.7)	17·1 (15·3 to 19·7)	9·1 (8·5 to 10·0)	−46·9 (−51·1 to −41·6)	2 110 000 (2 020 000 to 2 250 000)	1 650 000 (1 480 000 to 1 820 000)	−21·9 (−28·8 to −13·1)	30·3 (29·0 to 32·4)	20·8 (18·8 to 23·1)	−31·2 (−37·3 to −23·5)	29 500 000 (28 100 000 to 31 000 000)	40 000 000 (38 000 000 to 42 400 000)	423·8 (405·0 to 445·5)	507·4 (482·0 to 537·0)
Central Europe, eastern Europe, and central Asia	25 700 (25 600 to 25 700)	28 300 (28 300 to 28 400)	10·4 (10·0 to 10·9)	6·2 (6·2 to 6·2)	6·8 (6·8 to 6·8)	9·0 (8·6 to 9·5)	80 000 (68 200 to 93 900)	170 000 (137 000 to 212 000)	112·6 (77·8 to 145·8)	19·4 (16·5 to 22·8)	40·7 (32·7 to 50·7)	109·9 (75·5 to 142·7)	489 000 (335 000 to 702 000)	1 570 000 (1 240 000 to 1 940 000)	118·5 (81·1 to 170·3)	375·5 (296·3 to 463·9)
High income	17 400 (17 400 to 17 400)	12 200 (12 200 to 12 200)	−29·6 (−29·7 to −29·6)	1·7 (1·7 to 1·7)	1·1 (1·1 to 1·1)	−33·2 (−33·2 to −33·1)	106 000 (77 500 to 137 000)	110 000 (67 800 to 151 000)	3·9 (−18·9 to 15·9)	10·2 (7·5 to 13·2)	10·1 (6·2 to 13·8)	−1·3 (−23·0 to 10·1)	2 390 000 (1 580 000 to 3 190 000)	3 130 000 (2 040 000 to 4 190 000)	230·1 (152·2 to 307·5)	287·1 (186·9 to 383·6)
Latin America and Caribbean	45 300 (43 000 to 48 800)	38 200 (36 900 to 39 800)	−15·6 (−19·5 to −12·0)	8·5 (8·1 to 9·2)	6·4 (6·2 to 6·7)	−24·7 (−28·1 to −21·4)	98 600 (87 800 to 114 000)	121 000 (94 500 to 154 000)	23·0 (5·6 to 38·3)	18·6 (16·6 to 21·5)	20·4 (15·9 to 25·8)	9·8 (−5·8 to 23·5)	992 000 (754 000 to 1 300 000)	1 740 000 (1 330 000 to 2 220 000)	187·0 (142·1 to 245·7)	293·0 (223·9 to 373·7)
North Africa and Middle East	7 900 (5 540 to 11 500)	11 100 (6 900 to 22 300)	40·7 (−13·2 to 191·3)	1·5 (1·1 to 2·2)	1·8 (1·1 to 3·6)	16·9 (−27·9 to 142·1)	16 100 (10 400 to 25 300)	26 800 (11 400 to 70 400)	66·1 (−0·6 to 230·9)	3·1 (2·0 to 4·9)	4·3 (1·8 to 11·3)	38·1 (−17·4 to 175·0)	118 000 (81 100 to 171 000)	229 000 (143 000 to 407 000)	22·8 (15·7 to 32·9)	36·8 (22·9 to 65·3)
South Asia	119 000 (95 000 to 150 000)	45 800 (34 400 to 67 800)	−61·6 (−71·4 to −45·3)	7·5 (6·0 to 9·4)	2·5 (1·9 to 3·7)	−67·0 (−75·5 to −52·9)	152 000 (116 000 to 198 000)	98 400 (59 800 to 172 000)	−35·4 (−56·5 to −0·1)	9·6 (7·3 to 12·5)	5·3 (3·2 to 9·3)	−44·5 (−62·6 to −14·1)	1 890 000 (1 640 000 to 2 170 000)	2 110 000 (1 770 000 to 2 570 000)	118·9 (103·3 to 136·4)	114·3 (95·9 to 139·4)
Southeast Asia, east Asia, and Oceania	68 500 (63 300 to 76 000)	67 400 (58 200 to 78 300)	−1·6 (−22·3 to 21·9)	3·4 (3·1 to 3·8)	3·1 (2·7 to 3·6)	−9·3 (−28·4 to 12·4)	149 000 (125 000 to 196 000)	141 000 (108 000 to 195 000)	−5·6 (−17·6 to 6·7)	7·4 (6·2 to 9·7)	6·5 (4·9 to 8·9)	−13·0 (−24·0 to −1·6)	1 650 000 (1 230 000 to 2 230 000)	2 190 000 (1 590 000 to 3 120 000)	82·1 (61·1 to 110·8)	100·0 (72·6 to 142·7)
Sub-Saharan Africa	907 000 (811 000 to 1 050 000)	515 000 (467 000 to 581 000)	−43·2 (−47·4 to −38·3)	106·5 (95·2 to 123·5)	45·5 (41·2 to 51·3)	−57·3 (−60·5 to −53·6)	1 500 000 (1 400 000 to 1 660 000)	978 000 (820 000 to 1 150 000)	−35·0 (−43·0 to −25·0)	176·7 (164·9 to 194·7)	86·3 (72·4 to 101·3)	−51·2 (−57·2 to −43·6)	21 900 000 (21 500 000 to 22 500 000)	29 100 000 (28 000 000 to 30 400 000)	2575·3 (2522·2 to 2637·9)	2565·2 (2466·7 to 2685·6)

Data are shown as estimates with 95% uncertainty intervals in parentheses. GBD=Global Burden of Diseases, Injuries, and Risk Factors Study.

The number of HIV-related deaths decreased to 718 000 (95% UI 669 000 to 785 000) in 2021, less than half of its peak of 1·60 million (1·34 to 1·88) in 2004 (figure 1). Numbers of deaths were similar among males (355 000, 331 000 to 390 000) and females (363 000, 331 000 to 407 000) in 2021 (figure 1). The largest number of HIV deaths was in sub-Saharan Africa (515 000, 467 000 to 581 000), despite the region having the largest reduction in HIV deaths from 2000 to 2021. The three super-regions with the largest reductions in HIV deaths from 2010 to 2021 were sub-Saharan Africa, south Asia, and high-income countries (table). By contrast, the super-regions that had increases in HIV deaths over the same time period were central Europe, eastern Europe, and central Asia (10·4% [10·0 to 10·9]) and north Africa and the Middle East (40·7% [–13·2 to 191·3]; table).

The global HIV mortality rate in 2021 was 9·1 per 100 000 population (95% UI 8·5 to 10·0). Mortality rates remain highest in sub-Saharan Africa (45·5 per 100 000, 41·2 to 51·4) and lowest in high-income countries (1·1 per 100 000, 1·1 to 1·1; table). Within the sub-Saharan Africa super-region, southern sub-Saharan Africa had the largest burden of HIV deaths in 2021 (168·1 per 100 000, 158·9 to 178·6) and central sub-Saharan Africa had the smallest burden of HIV deaths (34·2 per 100 000, 27·8 to 43·5; figure 2). Across most countries, the HIV mortality rate has decreased considerably over time, particularly with the availability and scale-up of ART. Since 2010, HIV mortality rates have decreased by 46·9% (41·6 to 51·1; figure 1). This decrease includes large declines in south Asia (67·0%, 52·9 to 75·5) and sub-Saharan Africa (57·3%, 53·6 to 60·5; table). However, HIV mortality rates increased from 2010 to 2021 in two regions concurrently experiencing increases in new HIV infections: north Africa and the Middle East (16·9%, –27·9 to 142·1) and central Europe, eastern Europe, and central Asia (9·0%, 8·6 to 9·5; table). By country, age-standardised HIV mortality rates in 2021 were highest in Lesotho (359·8 per 100 000, 309·6 to 421·5), Eswatini (256·7 per 100 000, 218·1 to 305·8), and Botswana (218·6 per 100 000, 177·9 to 273·3), although rates were substantially reduced from their peaks before the availability of ART (figure 2B; appendix 2 p 7). In countries such as Armenia, Georgia, Mongolia, and Uzbekistan (central Asia); Estonia, Latvia, Lithuania, and Russia (eastern Europe); and Iran, Lebanon, Sudan, and Tunisia (north Africa and the Middle East), HIV mortality rates increased from 2010 to 2021 (appendix 2 p 7).

In our forecasting, we found that global progress is likely to fall short of the interim 2025 targets for new HIV infections (<370 000) and HIV-related deaths (<250 000). At the current trajectory, we project only gradual declines in global HIV incidence, from 1·43 million (95% UI 1·29–1·59) in 2025 to 1·30 million (1·16–1·48) in 2030 and 926 000 (718 000–1 602 000) by 2050 (figure 1). We estimated similar declines in mortality, with 615 000 (567 000–680 000) global HIV-related deaths in 2025, 513 000 (462 000–597 000) in 2030, and 327 000 (236 000–586 000) by 2050. Although HIV incidence and mortality are declining globally, the number of people living with HIV continues to increase. By 2030, we project 43·7 million (40·5–47·0) people will be living with HIV, and this number will continue to increase until a peak of 44·4 million (40·7–49·8) in 2039 before beginning a slow decline. By 2050, we project 43·4 million (38·7–55·0) people will be living with HIV worldwide.

By 2030 in sub-Saharan Africa, we project 711 000 (95% UI 590 000–848 000) new HIV infections and 348 000 (308 000–409 000) HIV-related deaths. By 2050, we project 431 000 (348 000–543 000) new HIV infections and 161 000 (132 000–196 000) HIV-related deaths (appendix 2 p 3). The super-regions of southeast Asia, east Asia, and Oceania; Latin America and the Caribbean; central Europe, eastern Europe, and central Asia; and high-income countries are all projected to experience overall declines in both HIV incidence and mortality, although at substantially different rates (appendix 2 p 3).

Focusing on the UNAIDS 2030 targets (compared with the baseline of 2010), only two countries are forecasted to achieve 90% reductions in HIV incident cases, and two countries are forecasted to achieve 90% reductions in HIV-related deaths (appendix 2 p 4). Looking beyond 2030, 11 countries and territories are projected to achieve these reductions in incidence and three in mortality by 2040, with 23 countries and territories set to reach the incidence targets and 14 to reach the mortality targets by 2050 (appendix 2 p 5).

**Figure 3:**
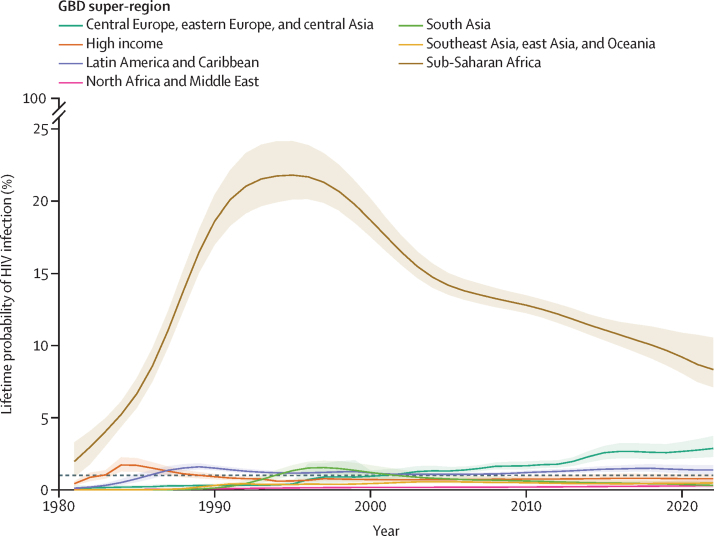
Period lifetime probability of HIV infection by GBD super-region Each line represents the period lifetime probability of HIV acquisition for each GBD super-region. The shaded areas represent 95% uncertainty intervals. GBD=Global Burden of Diseases, Injuries, and Risk Factors Study.

From 1990 to 2021, period lifetime probability of HIV acquisition was highest in the sub-Saharan Africa super-region, with a maximum of 21·8% (95% UI 20·1–24·2) in 1995 (figure 3). The 2021 lifetime probability in sub-Saharan Africa was 8·7% (7·5–10·7), a 60% reduction from its peak 26 years earlier. Increases in incidence rates over the past decade in central Europe, eastern Europe, and central Asia elevated the lifetime probability in the super-region to 2·8% (2·2–3·5) in 2021, the highest probability seen outside of sub-Saharan Africa. Four of the seven super-regions had probabilities less than 1% in 2021, a possible threshold to use as a target for tracking progress towards the end of AIDS as a public health threat.

**Figure 4:**
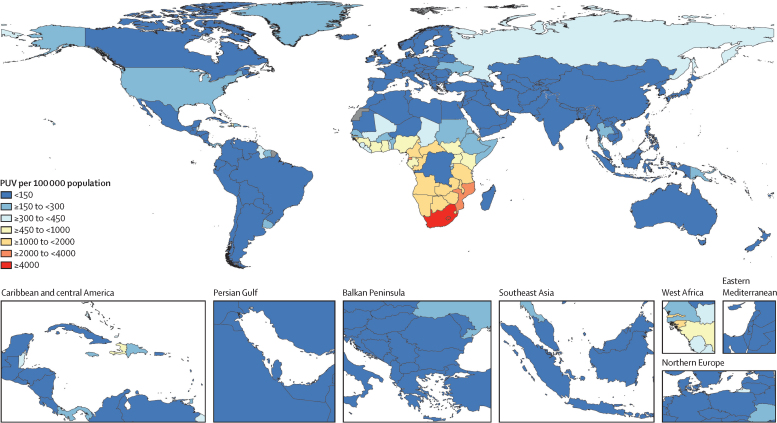
Map of PUV in 2021 PUV=prevalence of unsuppressed viraemia.

The geographical distribution of all-ages, both-sexes PUV in 2021 varied substantially across countries (figure 4). In comparison with other super-regions, the highest prevalence of PUV was within sub-Saharan Africa, particularly in southern sub-Saharan Africa. In 2021, South Africa, Lesotho, Equatorial Guinea, and Mozambique had the highest PUV, with all exceeding 2000 cases per 100 000 population. Five countries had PUVs per 100 000 population of between 1500 and 2000: Botswana, Eswatini, Central African Republic, Tanzania, and Guinea-Bissau. At the super-region level, PUV per 100 000 in 2021 varied from 24·5 (95% UI 10·3–53·5) in north Africa and the Middle East and 46·3 (10·6–96·5) in southeast Asia, east Asia, and Oceania to 163·5 (68·9–270·6) in central Europe, eastern Europe, and central Asia and 999·9 (857·4–1154·2) in sub-Saharan Africa (appendix 2 p 8). In terms of the percentage change in PUV between 2003 (the global peak year) and 2021, the largest reductions in PUV were observed in south Asia (66·2%, 47·9–80·7) and sub-Saharan Africa (64·5%, 58·8–69·4), and the largest increase in PUV was seen in central Europe, eastern Europe, and central Asia (116·1%, 8·0–218·2).

## Discussion

Globally, substantial progress has been made in reducing HIV incidence and mortality, with particularly notable reductions in sub-Saharan Africa and improvements in several other regions, such as south Asia, leading the way in preventing new HIV infections and scaling up treatment. Progress has been uneven, however, despite the existence of effective prevention tools and treatment. In the super-regions of north Africa and the Middle East and central Europe, eastern Europe, and central Asia, for example, both HIV incidence and mortality are increasing. The primary findings associated with the lifetime probability of HIV are continued high levels of risk in sub-Saharan Africa, despite substantial declines from the peak. Additionally, we estimate multiple super-regions have lifetime probabilities higher than 1%, the threshold we use to contextualise achievements in lifetime probability reduction. Additionally, in 2021, sub-Saharan Africa had the highest PUV despite large reductions since 2003, and the central Europe, eastern Europe, and central Asia super-region experienced a marked increase in PUV during the same period. Taken together, these findings reflect both the remarkable progress made in controlling the epidemic and the remaining room for improvement, playing out at different rates in different regions of the world.

Comparing estimates of HIV incidence, prevalence, and mortality between GBD and those published by UNAIDS and other groups can be helpful in highlighting key methodological differences and perspectives. For example, UNAIDS estimated 38·7 million (95% UI 32·8–45·2) people living with HIV globally, 1·4 million (1·1–1·8) new HIV infections, and 660 000 (500 000–920 000) HIV-related deaths for 174 countries in 2021, of which 143 countries' estimates are publicly available, compared with the GBD estimates for the same year of 40·0 million (95% UI 38·0–42·4), 1·65 million (1·48–1·82), and 718 000 (669 000–785 000), respectively, in 204 countries and territories. These differences in incidence and mortality estimates reflect differences in the disease progression and mortality parameters used in our respective HIV simulations, along with differences in data used. The primary source of global differences is sub-Saharan Africa, which has the highest HIV burden. Our process of triangulating mortality estimates with the implied level of HIV deaths estimated through the life table process^31^ is a key difference in estimation approaches. The life table process incorporates all-cause mortality data and the discrepancies between a standard age pattern of mortality and the observed pattern to provide a secondary estimate of HIV mortality. We utilise this additional estimate of HIV mortality to leverage the information present in our demographic estimation process.

Regarding forecasting, several mathematical models of HIV have been applied to make the case for investing in treatment and prevention interventions. A recent example of this is the simulation models published in 2021 to predict the epidemiological impact of achieving interim targets towards HIV elimination by 2030, which were used to guide development of the interim 2025 targets.^32^ Similar forecasting and cross-validation with a global, internally consistent platform such as the GBD, and consideration of additional metrics, could allow for more robust and longer-term goal setting beyond 2030. With the ability to forecast HIV estimates at an individual country level out to 2050, the GBD can help to set ambitious yet realistic and achievable targets over the next 25 years.

Although successes in the global HIV response are to be celebrated, our estimates highlight serious challenges that remain. Steep increases in the number of new HIV infections in regions such as central Europe, eastern Europe, and central Asia and north Africa and the Middle East from 2010 to 2021, compared with the large decreases in sub-Saharan Africa over the same period, reflect stark geographical disparities impeding the elimination of HIV as a public health threat. In many settings, political commitment is insufficient to mobilise resources for marginalised communities that are disproportionately affected by new HIV infections, and discriminatory attitudes, stigma, punitive laws, and physical violence prevent people from accessing needed prevention and treatment services. Low coverage of ART, as well as inadequate access to prevention and harm-reduction services for key populations (eg, people who inject drugs), remain important barriers. Unfortunately, those who are at the highest risk of acquiring HIV are often those who face the greatest barriers across the HIV care cascade.^6^ In north Africa and the Middle East, for example, only 67% of people living with HIV are aware of their status, 50% access ART, and 45% are virally suppressed,^6^ compared with 92%, 83%, and 77%, respectively, in eastern and southern Africa in 2022. For this reason, the north Africa and the Middle East region is projected to experience long-term increases in HIV incidence and mortality if the current trajectory continues.^6^

Our results highlight the lifetime probability of acquiring HIV as a single metric for comparing risk of infection across locations and time.^21^, ^33^ This metric contrasts with all-age incidence rates in that it aligns the measure of acquisition risk with the lifelong nature of HIV infections. For example, a 5% lifetime risk of acquiring HIV is equivalent to exposure to an annual incidence rate of around 64 new infections per 100 000 person-years of exposure. Whereas 64 in 100 000 is difficult to interpret, a 5% chance of acquiring HIV over a lifetime is a straightforward way to characterise the risk associated with period incidence rates in an intuitive manner. Although this intuition might not be immediate, we have seen widespread adoption of life expectancy as a metric for comparison of period mortality rates across locations. As policy makers and implementers gain familiarity with the lifetime probability metric and perceive it to be a useful summary, goals codifying the end of AIDS as a public health threat could include benchmarks for lifetime probability of HIV. A trajectory of decreasing incidence alone does not constitute removing the threat of HIV. Sub-Saharan Africa has both the largest reductions in HIV incidence and the highest lifetime probability of HIV. Actual low levels of incidence must be achieved and maintained, and perhaps these can best be summarised using lifetime probability of HIV. A memorable and intuitive goal, such as reducing the lifetime probability of HIV to less than 1%, could galvanise support and influence policy and practice in a way that current all-age incidence goals struggle to do.

PUV captures the subset of HIV prevalence that needs to be addressed through treatment expansion, and therefore goals can focus on decreasing this metric over time. By contrast, increasing prevalence could be a good thing, reflecting increased survival among people living with HIV due to treatment. In this study, the highest estimates of PUV are in sub-Saharan Africa, implying a higher risk of HIV transmission occurring in this super-region compared with other super-regions. However, the patterns of PUV by sex show a more marked improvement among females due to higher coverage of ART than among males.^34^ Negative percentage changes in PUV between the global peak in 2003 and 2021 within sub-Saharan Africa are testament to the impact of the focused attention to HIV care and treatment that occurred in the super-region because of both local and global efforts. For super-regions with positive percentage changes in PUV, contributing factors could include late presentation of HIV, inadequate testing and treatment, and insufficient political will and urgency to address stigma, discrimination, and threatening legal environments that impede individuals' access to effective HIV services or implementation of national programmes against HIV/AIDS.^35^, ^36^, ^37^

Although not on track to meet either the 2025 interim targets or the 2030 targets to reduce new HIV infections and AIDS-related deaths by 90%, the global HIV response has certainly made an indelible, historic impact. Enormous success in reducing both HIV incidence and AIDS-related mortality across sub-Saharan Africa, for example, reflects the historic progress achieved over decades. Even in the absence of an HIV cure or an effective vaccine, the availability of ART and, importantly, the rapid scale-up of treatment programmes have been instrumental to this progress. Impressively, all four countries that are estimated to have achieved the UNAIDS 95-95-95 treatment targets (Eswatini, Botswana, Zimbabwe, and Rwanda) are in sub-Saharan Africa, as are half of the remaining 17 countries that are close to the goal line,^6^ a testament to the robust HIV epidemic response that sustained commitment and financial investment can achieve. Studies have found significant increases in rapid ART initiation rates after the 2015 WHO recommendation of universal HIV treatment for all people living with HIV regardless of CD4 cell count or disease stage, the so-called treat-all policy that is now widely adopted across countries in sub-Saharan Africa.^38^, ^39^, ^40^ Coupled with newer and improved first-line regimens that have greater tolerability and a higher barrier to resistance, safe, simple, and effective treatment of HIV is now available to the majority of people who need it.^1^, ^41^

Even with accessible treatment, challenges persist in many settings with starting people living with HIV on treatment and retaining people in care. HIV care retention is a dynamic process, with some individuals cycling in and out of care or adjusting their engagement with the health system over time.^42^ Beyond treatment, itself a crucial component of HIV prevention, the remarkable reduction in HIV incidence in sub-Saharan Africa can be attributed to a combination of biomedical and behavioural interventions, both of which are constantly evolving as new technologies come to market and different service delivery models are trialled. Commonly implemented HIV prevention approaches in sub-Saharan Africa, some of which were resounding successes and others with more mixed results, include the prevention of mother-to-child-transmission, voluntary male medical circumcision, condom use, multiple pre-exposure prophylaxis (PrEP) modalities including oral PrEP, long-acting injectable PrEP, and the vaginal ring, and behavioural and harm-reduction interventions.^28^, ^43^, ^44^, ^45^, ^46^, ^47^, ^48^, ^49^ Implementation of these HIV prevention strategies, particularly for those key populations who would benefit most, remains an ongoing priority for national and local HIV programmes.^50^ Continued investment and expansion of HIV prevention strategies are essential, necessitating financial resources and a comprehensive approach that confronts social and cultural barriers, as well as stigma and discrimination, to promote equitable access and utilisation.

Historically, external funding from the Global Fund to Fight AIDS, Tuberculosis and Malaria and the US President's Emergency Plan for AIDS Relief (PEPFAR) has enabled the expansion of HIV treatment and prevention programmes in many settings. Increasingly, national governments are being asked to take on a greater responsibility for funding their national HIV programmes with the goal of achieving a more sustainable financial model.^51^ Political commitment at the global level and, perhaps most importantly, at the national level is crucial for not only providing services but also fighting inequalities, reducing stigma and discrimination, and engaging with those communities who are most vulnerable or marginalised.^6^ At the time of writing, the US Congress has failed to reauthorise PEPFAR, leaving questions about long-term development assistance for countries that rely on substantial financial support to provide treatment to people living with HIV.^52^ Further investigation is required to assess the implications of discontinued external funding without domestic spending filling the gap, resulting in excess burden due to reduced ART coverage. The economic argument for aggressively preventing new infections is straightforward, given the large costs of providing lifetime access to ART to people living with HIV.

In the context of uncertain domestic and international funding for health, global inequalities in access to treatment and care, and emerging natural and societal challenges due to climate change,^53^ measuring and producing key HIV metrics and providing consistent estimates and forecasts can only be a first step. To end HIV as a public health threat, a reinvigorated, coordinated, and self-sustaining global HIV response effort will be essential. Moving forward, accelerating the global decline in new infections will be crucial not only for reaching the 2030 targets (unlikely at current trajectories) but also for long-term epidemic control. We offer the following policy recommendations: first, optimise treatment coverage, with highly effective and robust ART (including long-acting formulations) for all. This step means aligning treatment and care delivery with the preferences and choices of individuals, whenever possible. Data from additional metrics, such as PUV, can help with understanding how well national programmes are achieving viral suppression or areas in which to improve. Second, sharpen the focus on prevention, with a particular emphasis on tailoring prevention modalities to fit the individual and specifically engaging priority populations for prevention. PrEP can be instrumental in these efforts but must be implemented effectively and reach those who will benefit most. Third, for those people who do acquire HIV infection, prompt diagnosis and linkage to care is possible with widely available and frequent HIV testing. All individuals must be empowered to understand their risks and access facility-based testing and self-testing, free from stigma, financial burden, or other barriers to testing. Although treatment, prevention, and diagnosis are the cornerstones of the current HIV response, optimising, tailoring, and expanding access to these services offers the opportunity to get closer to our shared goal of eventual elimination of HIV.

A primary strength of this study is the alignment between HIV burden estimates, burden estimates from other causes, and the all-cause envelope for all countries and regions, which ensures consistency across causes. Updated HIV burden estimates that are consistent with burden estimates for other causes within the GBD enable policy makers to evaluate the relative burden of HIV and develop appropriate strategies. Consistency across cause-specific estimates means that the total number of deaths is equal to the sum of cause-specific deaths, enabling a clear evaluation of the largest sources of burden in a population for targeting policy and resource allocation. Another strength is accounting for misclassified and unknown cause-of-death data in vital registration systems to improve HIV mortality estimates.^54^ Reclassification of deaths that should be classified as HIV enhances the comparability of mortality estimates made with data from vital registration systems of varying quality. Finally, the combined reporting of historical and forecasted estimates highlights the implications of continuation of recent trends in the level of HIV control. We report many metrics for the past and the future, allowing for progress made to be contrasted with the task that remains to achieve concrete goals.

This study has several limitations. Variations in data availability and quality motivate varying methods across locations. In most countries with generalised epidemics, prevalence data are available, whereas mortality data are sparse. By contrast, in many concentrated epidemic locations, high-quality vital registration data capture deaths, but these locations rarely have prevalence and treatment coverage and retention data. Although we seek to reflect the strength of available evidence through the propagation of uncertainty through draw-level estimation throughout our modelling process, it is difficult to account for all sources of uncertainty, including structural uncertainty (ie, uncertainty intervals are conditional on model choice, and we employ different models for different data contexts). Additionally, the timeline of the estimate production process limited our ability to incorporate newer data that were made available after the beginning of our production process but before manuscript submission. Finally, the mortality and disease progression parameters that drive our HIV simulation are estimated through the pooling of available data, resulting in three broad categories of parameter estimates: sub-Saharan Africa, high income, and other. This crude stratification reflects any appropriate primary classification of differences in rates, but evidence indicates location-specific differences in these rates, particularly by demographic stratification, that could affect our more granular estimates. Future work to develop location-specific transition parameter estimates could improve the precision of stratified estimates. Our forecasts reflect the single chosen approach to projecting ART coverage, and future work could investigate how burden projections vary according to various approaches to ART coverage projection through a sensitivity analysis. One opportunity for improvement would be to use a diffusion of innovations approach—a theory regarding the pace at which improvements made in one location can be adopted elsewhere—to model achievable coverage through a stochastic frontier model.^55^ Similarly, the projection of the transmission rate in our forecasts captures implicit assumptions about changes in non-ART HIV prevention activities. Explicitly capturing the impact of these programme activities on the transmission rate would expand the policy relevance of our forecasts. The introduction of the PUV metric brings a new focus to the role of viral suppression in our modelling. A limitation of this study is that we do not directly model viral suppression and its variation across time and locations. In future work, we would like to integrate data on viral suppression into location-specific transmission rates. Interrogation of age-specific variation in PUV could reveal interesting opportunities to prevent transmission of HIV across generations. Possible metrics to include in future manuscripts could build on the lifetime probability concept, such as looking at the lifetime probability of death from HIV versus non-HIV causes among people living with HIV.

In conclusion, to sustain and invigorate the global HIV response, with a forecast of 44 million people living with HIV by 2030 and still more than 1 million new infections each year, the global community will require leadership, commitment, collaboration, and innovation. Investments in HIV programmes will need to match increasing demand and be reflective of true partnership. Crucially, public health efforts, such as PEPFAR, which has helped to provide treatment to more than 20 million people living with HIV over two decades, need to be protected and strengthened. As the population of people living with HIV ages, health-care needs will evolve and include the management of other chronic diseases. Prevention services, with a multitude of existing and emerging technologies in the pipeline, can help to further reduce the number of new infections. Stigma, discrimination, punitive laws, and other obstacles to providing care and treatment must be eliminated, whereas key populations and marginalised communities who are at the greatest risk must be identified and receive special attention and necessary resources. With an abundance of tools and strategies at our disposal, interventions and care delivery models that work must be studied and implemented effectively and equitably. As a global community, decades into the fight, measuring and understanding progress and remaining gaps can help us chart the path towards our collective goal of ending the HIV epidemic.

### GBD 2021 HIV/AIDS Collaborators

### Contributors

### Data sharing

The data used in these analyses can be downloaded from the Global Health Data Exchange website at https://ghdx.healthdata.org/record/ihme-data/gbd-2021-hiv-1990-2050.

## Declaration of interests

S Afzal reports support for the present manuscript from King Edward Medical University; payment or honoraria for lectures, presentations, speakers bureaus, manuscript writing, or educational events from King Edward Medical University and collaborative partners including Johns Hopkins University, University of California, University of Massachusetts, KEMCAANA, and KEMCA_UK international scientific conferences; support for attending meetings or travel from King Edward Medical University; participation on a Data safety monitoring board or advisory board with National Bioethics Committee Pakistan, King Edward Medical University Ethical Review Board, and Ethical Review Board of Fatimah Jinnah Medical University, and Sir Ganga Ram Hospital; leadership or fiduciary roles in board, society, committee or advocacy groups, paid or unpaid with King Edward Medical University as Dean of Public Health and Preventive Medicine as well as Director of *Quality Enhancement Cell* and *Annals of King Edward Medical University* since 2014 as Chief Editor, Pakistan Association of Medical Editors as Member, Technical Expert Advisory Group of the Government as Member, Faculty of Public Health Royal Colleges UK (FFPH) as Fellow, Society of Prevention, Advocacy And Research, King Edward Medical University (SPARK) as Member, Pakistan Society of Infectious Diseases, Technical Expert Advisory Group of the Government as Member, Scientific Session, KEMCA-UK as Advisory Board Member and Chair, International Scientific Conference, KEMCAANA as Chairperson, Research and Publications Higher Education Commission, HEC Pakistan as Member, Research and Journals Committee Pakistan Medical and Dental Council, Pakistan as Member, National Bioethics Committee, Pakistan as Member, Corona Experts Advisory Group as Member, Technical Working Group for Infectious Diseases as Member, Dengue Experts Advisory Group as Member, and Punjab Residency Program Research Committee as Chair. T W Bärnighausen reports grants or contracts from the National Institutes of Health (NIH), Alexander von Humboldt Foundation, German National Research Foundation (DFG), EU, German Ministry of Educational and Research, Germany Ministry of the Environment, Wellcome, and KfW; payment or honoraria for lectures, presentations, speakers bureaus, manuscript writing, or educational events from PLOS as Editor-in-Chief; participation on a data safety monitoring board or advisory board with scientific advisory boards for NIH-funded research projects in Africa on climate change and health; and stock or stock options in CHEERS. A Beloukas reports grants or contracts from Gilead and GSK to the University of West Attica; payment or honoraria for lectures, presentations, speakers bureaus, manuscript writing, or educational events from Gilead and GSK; support for attending meetings or travel from Gilead and GSK; receipt of equipment, materials, drugs, medical writing, gifts, or other services from Cepheid. C S Brown reports support from occasional contributions to market research, outside the submitted work. L Degenhardt reports leadership or fiduciary roles in board, society, committee or advocacy groups, paid or unpaid, with AIDS Council of NSW Board as the volunteer Vice President of the Board. I M Ilic reports support for the present manuscript from Ministry of Education, Science and Technological Development, Republic of Serbia for project number 175042, 2011–2023. N E Ismail reports leadership or fiduciary roles in board, society, committee, or advocacy groups, paid or unpaid, with Malaysian Academy of Pharmacy, Malaysia, as Bursar and Council Member, and Malaysian Pharmacists Society Education Chapter, Malaysia, as Committee Member. K Krishan reports non-financial support from the UGC Centre of Advanced Study, CAS II, awarded to the Department of Anthropology, Panjab University, Chandigarh, India. M Li reports grants or contracts from the National Science and Technology Council, Taiwan (NSTC 112-2410-H-003–031); leadership or fiduciary roles in board, society, committee, or advocacy groups, paid or unpaid, with the *Journal of the American Heart Association* as a Technical Editor. W Mendoza reports support in his role as Program Analyst in Population and Development at the UN Population Fund Country Office in Peru, an institution which does not necessarily endorse this study. P Meylakhs reports support for the present manuscript and grants or contracts from the Russian Science Foundation (grant 23-15-00428). L Monasta acknowledges support for the present manuscript from the Italian Ministry of Health (Ricerca Corrente 34/2017), payments made to the Institute for Maternal and Child Health IRCCS Burlo Garofolo. E A Mpolya reports support for the present manuscript and support for attending meetings or travel from the Department of State, USA, which provided funding for a Fulbright Senior Visiting Fellowship at the Institute for Health Metrics and Evaluation between August, 2023, and April, 2024. A P Okekunle reports support for the present manuscript and support for attending meetings or travel from the National Research Foundation of Korea funded by the Ministry of Science and ICT (2020H1D3A1A04081265). C Palladino reports grants or contracts from Fundação para a Ciência e a Tecnologia, I P (national funding), under a contract programme as defined by DL number 57/2016 and law number 57/2017 (DL57/2016/CP1376/CT0004; DOI 10.54499/DL57/2016/CP1376/CT0004). V C F Pepito reports grants or contracts from Sanofi Consumer Healthcare and the International Initiative for Impact Evaluation. A Sharifan reports leadership or fiduciary roles in board, society, committee, or advocacy groups with Cochrane as an unpaid steering member of the Cochrane Early Career Professionals Network; receipt of equipment, materials, drugs, medical writing, gifts, or other services from Elsevier and Cochrane. S Shrestha reports support from the School of Pharmacy, Monash University Malaysia, as recipient of a Graduate Research Merit Scholarship. J A Singh reports consulting fees from ROMTech, Atheneum, Clearview Healthcare Partners, American College of Rheumatology, Yale, Hulio, Horizon Pharmaceuticals, DINORA, Frictionless Solutions, Schipher, Crealta/Horizon, Medisys, Fidia, PK Med, Two Labs, Adept Field Solutions, Clinical Care Options, Putnam Associates, Focus Forward, Navigant Consulting, Spherix, MedIQ, Jupiter Life Science, UBM, Trio Health, Medscape, WebMD, Practice Point Communications, and NIH; payment or honoraria for speakers bureaus from Simply Speaking; support for attending meetings or travel from OMERACT as a past steering committee member; leadership or fiduciary roles in board, society, committee, or advocacy groups, paid or unpaid with OMERACT as a past steering committee member, Veterans Affairs Rheumatology Field Advisory Committee as Chair, and USA Cochrane Musculoskeletal Group Satellite Center on Network Meta-analysis as Editor and Director; stock or stock options in Atai Life Sciences, Kintara Therapeutics, Intelligent Biosolutions, Acumen Pharmaceutical, TPT Global Tech, Vaxart Pharmaceuticals, Atyu Biopharma, Adaptimmune Therapeutics, GeoVax Labs, Pieris Pharmaceuticals, Enzolytics, Seres Therapeutics, Tonix Pharmaceuticals Holding Corp, Aebona Pharmaceuticals, and Charlotte's Web Holdings, as well as previously owned stock options in Amarin, Viking, and Moderna Pharmaceuticals. A C Tsai reports payment or honoraria for lectures, presentations, speakers bureaus, manuscript writing, or educational events from Elsevier; participation on a data safety monitoring board for *Piloting Risk Stratification and Tailored Interventions with Pregnant and Postpartum Women with HIV in Kenya to Prevent Disengagement from Care and Viral Failure* (NIH R34MH126857, MPI: L Abuogi, M Onono, J Turan); leadership or fiduciary roles in board, society, committee, or advocacy groups, paid or unpaid, with Interdisciplinary Association for Population Health Science as a member of the Board of Directors. M Zielińska reports support as an AstraZenaca employee. A Zumla reports support for the present manuscript from the Pan-African Network on Emerging and Re-Emerging Infections (PANDORA-ID-NET) funded by the EDCTP—the EU Horizon 2020 Framework Programme; grants or contracts from UK NIHR Senior Investigator Award, Mahathir Science Award, and EU-EDCTP Pascoal Mocumbi Prize Laureate; participation on a data safety monitoring board or advisory board with WHO Infection Prevention Control Acute Respiratory Infections Guideline Development Group as a Member; leadership or fiduciary roles in board, society, committee or advocacy groups, paid or unpaid, with the Institute of Medicine, University of Bolton, UK, as a Board Member.
