# Supplementary material for: Global, regional, and national burden of HIV/AIDS, 1990–2021, and forecasts to 2050, for 204 countries and territories: the Global Burden of Disease Study 2021
Source: Lancet HIV. 2024 Nov 25;11(12):e807–22. doi: 10.1016/S2352-3018(24)00212-1 (PMC11612058; doi:10.1016/S2352-3018(24)00212-1)
Supplement: Supplementary appendix 1 [file mmc1.pdf]

THE LANCET
HIV

Supplementary appendix 1

This appendix formed part of the original submission and has been peer reviewed. We post it as supplied by the authors.

Supplement to: GBD 2021 HIV Collaborators. Global, regional, and national burden of HIV/AIDS, 1990–2021, and forecasts to 2050, for 204 countries and territories: the Global Burden of Disease Study 2021. *Lancet HIV* 2024; published online Nov 25. https://doi.org/10.1016/S2352-3018(24)00212-1.

Appendix 1

Supplementary Methods Appendix to "Global, regional, and national burden of HIV/AIDS, 1990-2021, and forecasts to 2050, for 204 countries and territories: the Global Burden of Diseases Study 2021"

This appendix provides further methodological detail for "Global, regional, and national burden of HIV/AIDS, 1990-2021, and forecasts to 2050, for 204 countries and territories: the Global Burden of Diseases Study 2021"

Portions of this appendix have been reproduced or adapted from previous Global Burden of Diseases (GBD) efforts.[1,2] References are provided for reproduced sections.

Contents

Section 1. High Level Methods 3
1.1. Case definition 3
1.2. Country Groupings.................................... 3
Section 2. Input Data 4
2.1. Prevalence Data 4
2.2. GBD demographic inputs 4
2.3. Data from countries.................................... 4
2.4. Changes for GBD 2021.................................... 5
2.5. Vital registration data.................................... 5
2.6. On-ART mortality literature data.................................... 5
2.7. Off-ART mortality and CD4 progression literature data 6
Section 3. Modeling HIV 7
3.1. On–ART, off–ART mortality and CD4 progression parameters.................................... 8
3.2. Disease model 9
3.2.1 Group 1 - EPP–ASM 10
3.2.2. Group 2 - Spectrum.................................... 11
3.2.3. Group 2 – Countries Without Survey Data and Vital Registration Data 12
3.3. Spatiotemporal Gaussian Process Regression (ST–GPR) 13
3.4. Additional Adjustments 13
Section 4. Forecasting HIV 14
4.1. Forecasting ART Coverage.................................... 14
4.2. Forecasting the Transmission rate 15
Section 5. Key Metrics 16
5.1. Prevalence of Unsuppressed Viremia 16
5.2. Lifetime Probability of Acquiring HIV 17
Section 6. Statement of GATHER compliance 19
Section 7. References 21

Section 1. High Level Methods

1.1. Case definition

Infection with the human immunodeficiency virus (HIV) causes influenza-like symptoms during the acute period following infection and can lead to acquired immunodeficiency syndrome (AIDS) if untreated. HIV attacks the immune system of its host, leaving infected individuals more susceptible to opportunistic infections like tuberculosis. Although there are two different subtypes of HIV, HIV-1 and HIV-2, no distinction is made in our estimation process or presentation of results. For HIV, ICD-10 codes are B20-B24, C46-C469, D84.9; ICD-9 codes are 042-044, 112-118 (after 1980), 130 (after 1980), 136.3-136.8 (after 1980), 176.0-176.9 (after 1980), 279 (after 1980); and ICD-9 BTL codes are B184-B185.

1.2. Country Groupings

Countries were divided into groups: Groups 1A, 1B, and 2A, 2B, and 2C.

Group 1 includes countries with HIV prevalence data from antenatal care clinics or nationally- or subnationally-representative population-based seroprevalence surveys. Group 1A included countries with a peak of at least 0.5% prevalence, and Group 1B includes countries with a peak of at least 0.25% prevalence and vital registration completeness less than 65%.

The remaining countries made up Group 2, which are further subdivided in Group 2A, 2B and 2C based on availability of vital registration data. Group 2A consisted of countries with high-quality vital registration data; Group 2B consisted of countries with available vital registration data that is not high-quality; and Group 2C countries were those without any vital registration data. Quality was measured based on a star rating system as described elsewhere.[3]

Both groups of countries relied on the same approach to modelling on- and off-antiretroviral therapy (ART) mortality, as described below.

Results were aggregated by super region as defined by the Global Burden of Disease study. These super regions are depicted in figure S2.

Figure S1. HIV specific country groupings based on data availability.

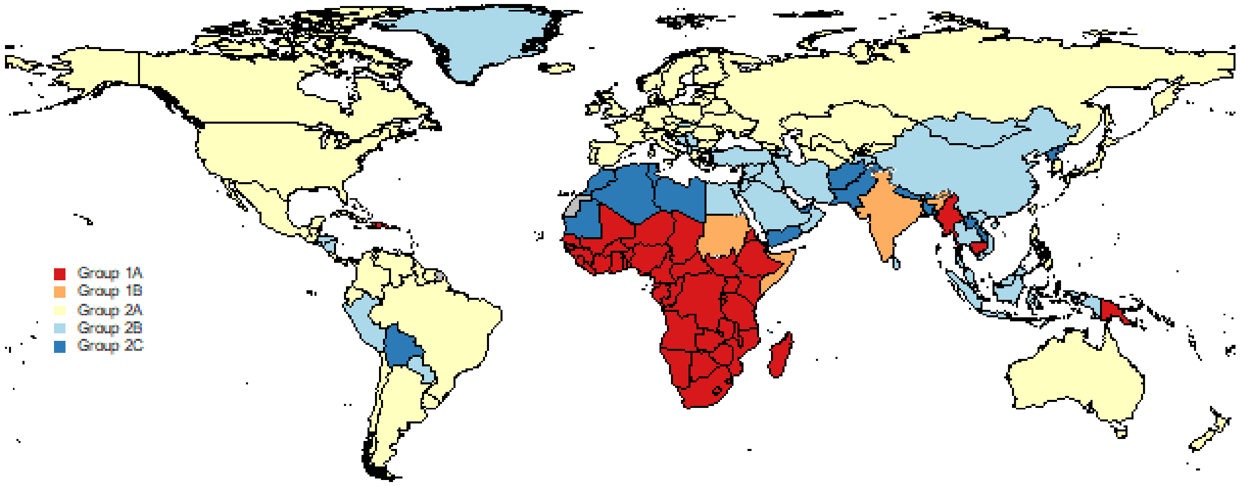

Figure S2. Global Burden of Disease 2021 super regions.

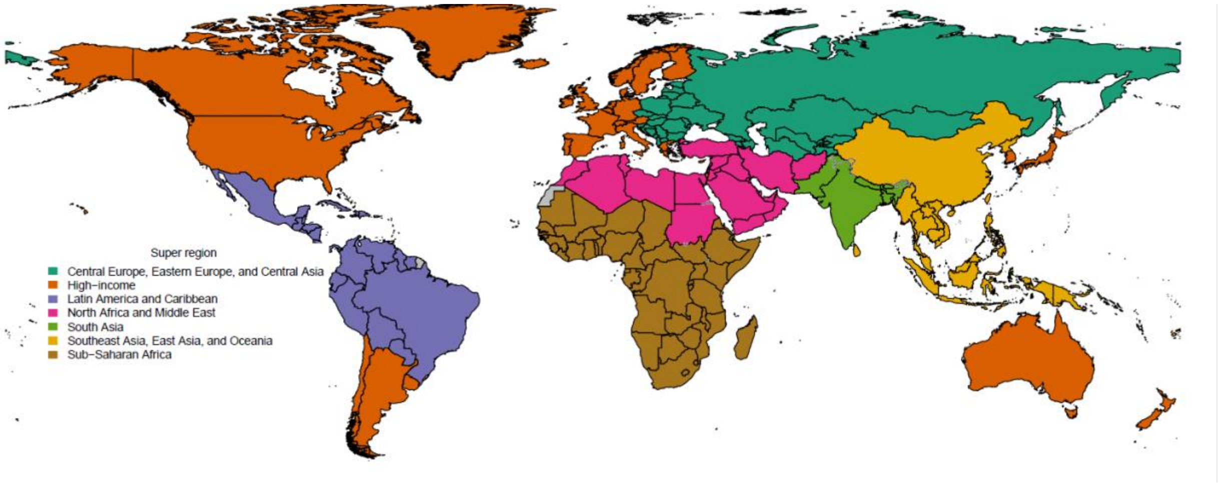

Section 2. Input Data

2.1. Prevalence Data

Household seroprevalence surveys

Geographically representative HIV seroprevalence survey results were used as inputs to the model for countries with generalised HIV epidemics where available. From these surveys, we used age- and sex-specific prevalence data.

2.2. GBD demographic inputs

Location-specific population, births, fertility, migration, and HIV-free survival rates from GBD 2021 were used as inputs in modelling all locations.

2.3. Data from countries

The files compiled by UNAIDS for their HIV/AIDS estimation process were our main source of data for producing estimates of HIV burden. The files are often built by within-country experts with the support of UNAIDS, which publishes estimates annually on behalf of countries and only shares their files when permission is granted. The files contain the HIV-specific information which is needed to run the Spectrum[4], and Estimation and Projection Package-Age Sex (EPP-ASM)[5] models. Spectrum and EPP-ASM require the following input data: AIDS mortality among people living with HIV with and without ART, CD4 progression among people living with HIV not on ART, ART coverage among adults and children, cotrimoxazole coverage among children, coverage of breastfeeding among women living with HIV, prevention of mother-to-child transmission (PMTCT) coverage, and CD4 thresholds for treatment eligibility. EPP-ASM additionally uses HIV prevalence data from surveillance sites and representative surveys. Antenatal care clinic (ANC) and treatment coverage data from UNAIDS were used in modelling Group 1 countries. We extracted all of these data from the proprietary format used by UNAIDS.

The EPP-ASM and Spectrum models used for GBD estimation vary slightly from those used by UNAIDS, with details on this variation included below. On top of the differences in model structure, we integrate our estimates of input model parameters, including new transition parameters and demographic rates. The differences between our estimates and UNAIDS' estimates reflect differences in model structure, model parameters, and the location-specific data used to calibrate our models.

2.4. Changes for GBD 2021

We supplemented the ANC and treatment coverage data available through processing done by the Local Burden of Disease team[6] and retrieving data on adult ART coverage rates from country reports, respectively. The addition of ANC sites affected 33 countries, while ART data were added in 45 countries. During the Local Burden of Disease alignment process, the ANC prevalence estimates were corrected in a number of facets. There were 17 estimates with placeholder sample sizes that were corrected, duplicate observations in Togo were removed, 123 additional observations were added, 1491 non-ANC observations were removed, and 232 points were outliered.

2.5. Vital registration data

We used all available sources of vital registration and sample registration data from the GBD Causes of Death database after garbage code redistribution and HIV/AIDS mis-coding correction in Group 2 countries and India[3,7]. There are two different cause of death data sources for HIV/AIDS in China: the Disease Surveillance Points (DSP) system and the Notifiable Infectious Disease Reporting (NIDR) system. Both systems are administered by the Chinese Center for Disease Control and Prevention, but the reported number of deaths due to HIV is significantly lower in DSP. Therefore, we have used the provincial-level ratio of deaths due to HIV/AIDS from NIDR to those from DSP, choosing the larger ratio between years 2013 and 2014, and scaled the reported deaths in the DSP system, which is in turn used in the spatiotemporal Gaussian process regression (ST-GPR).

2.6. On-ART mortality literature data

Data were identified by using search string: "hiv"[MeSH Terms] OR "hiv"[All Fields]) AND ("mortality"[Subheading] OR "mortality"[All Fields] OR "mortality"[MeSH Terms]) AND antiretroviral[All Fields] AND ("therapy"[Subheading] OR "therapy"[All Fields] OR "therapeutics"[MeSH Terms] OR "therapeutics"[All Fields]) in PubMed.

To be included, studies must include only HIV-positive people over the age of 15 who receive antiretroviral therapy (ART) but who were ART-naïve prior to the study. In addition, studies must report either a duration-specific (time since initiation of ART) mortality proportion or a hazard ratio across age or sex, and must not include children.

For duration-specific survival data, studies must report uncertainty on mortality estimates or provide stratum-specific sample sizes and must include duration-specific data to allow for calculation of 0–6, 7–12, or 13–24-month conditional mortality. In addition, studies must either report separate mortality and loss-to-follow-up (LTFU) curves, be corrected for LTFU using vital registration data or double sampling, or be conducted in a high-income setting. Finally, studies must report the percentage of participants who are male and the median age of participants.

Hazard ratio data for ages or sexes can only be used if the hazard ratios are controlled for other variables of interest (age, sex, and CD4 category). In GBD 2021, we included 61 studies, 13 of which were new this cycle. Of these studies, we added ten to inform the estimation age-sex hazard ratios, and three studies informed LFTU curves.

PRISMA flow diagram for GBD 2021 on-ART systematic review

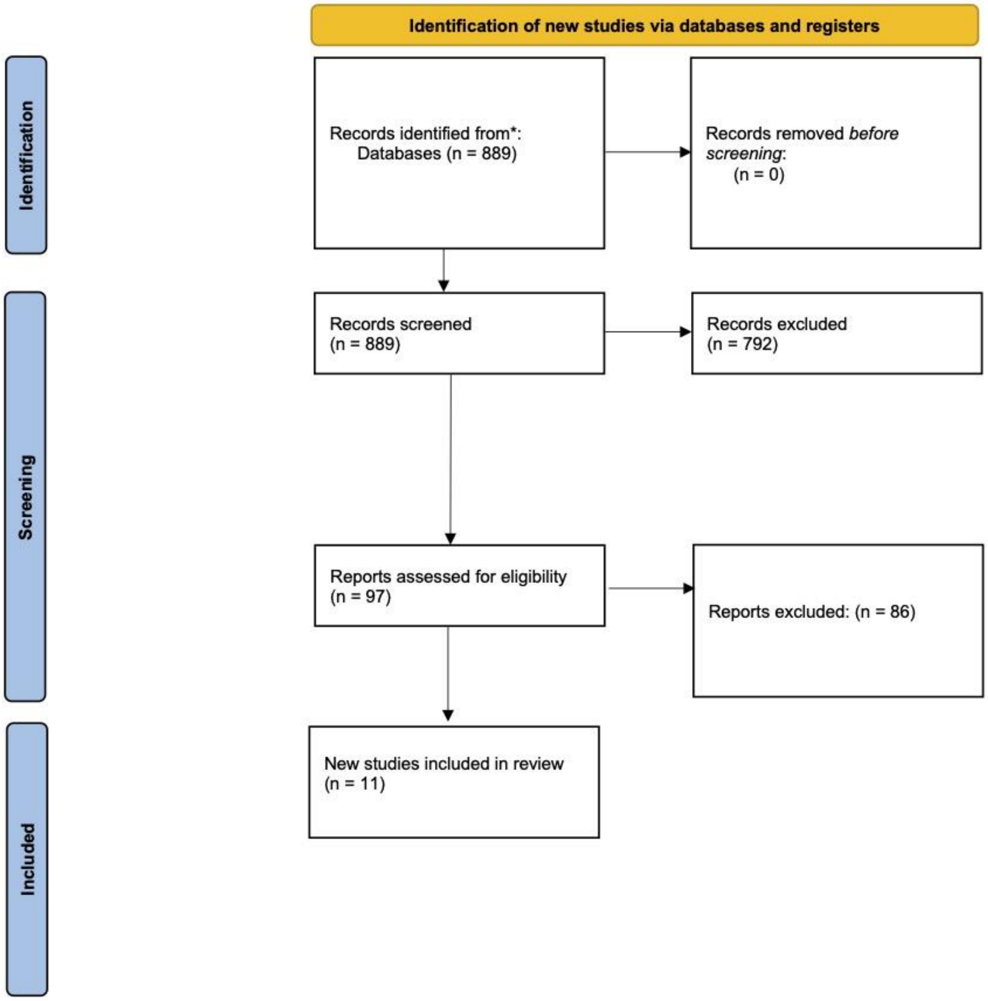

*Note: This systematic review was an update to the GBD 2019 review and doubled as a historical review of sources to capture previously missed studies. As a result, the number of sources being reviewed and excluded are ongoing.

From: Page MJ, McKenzie JE, Bossuyt PM, Boutron I, Hoffmann TC, Mulrow CD, et al. The PRISMA 2020 statement: an updated guideline for reporting systematic reviews. BMJ 2021;372:n71. doi: 10.1136/bmj.n71

For more information, visit: http://www.prisma-statement.org/

2.7. Off-ART mortality and CD4 progression literature data

In GBD 2013, we systematically reviewed the literature on mortality without ART to characterise uncertainty in the progression and death rates. We searched terms related to pre-ART or ART-naïve survival since seroconversion.[8] After screening, we identified 13 cohort studies that included the cohorts used by UNAIDS, from which we extracted survival at each one-year point after infection. Screening for additional, recently published studies in GBD 2015, GBD 2016, and GBD 2017 identified no new cohort studies for inclusion in this analysis. We did not search for new studies in GBD 2019 or GBD 2021.

Section 3. Modeling HIV

Figure S3: Flow chart of modelling process

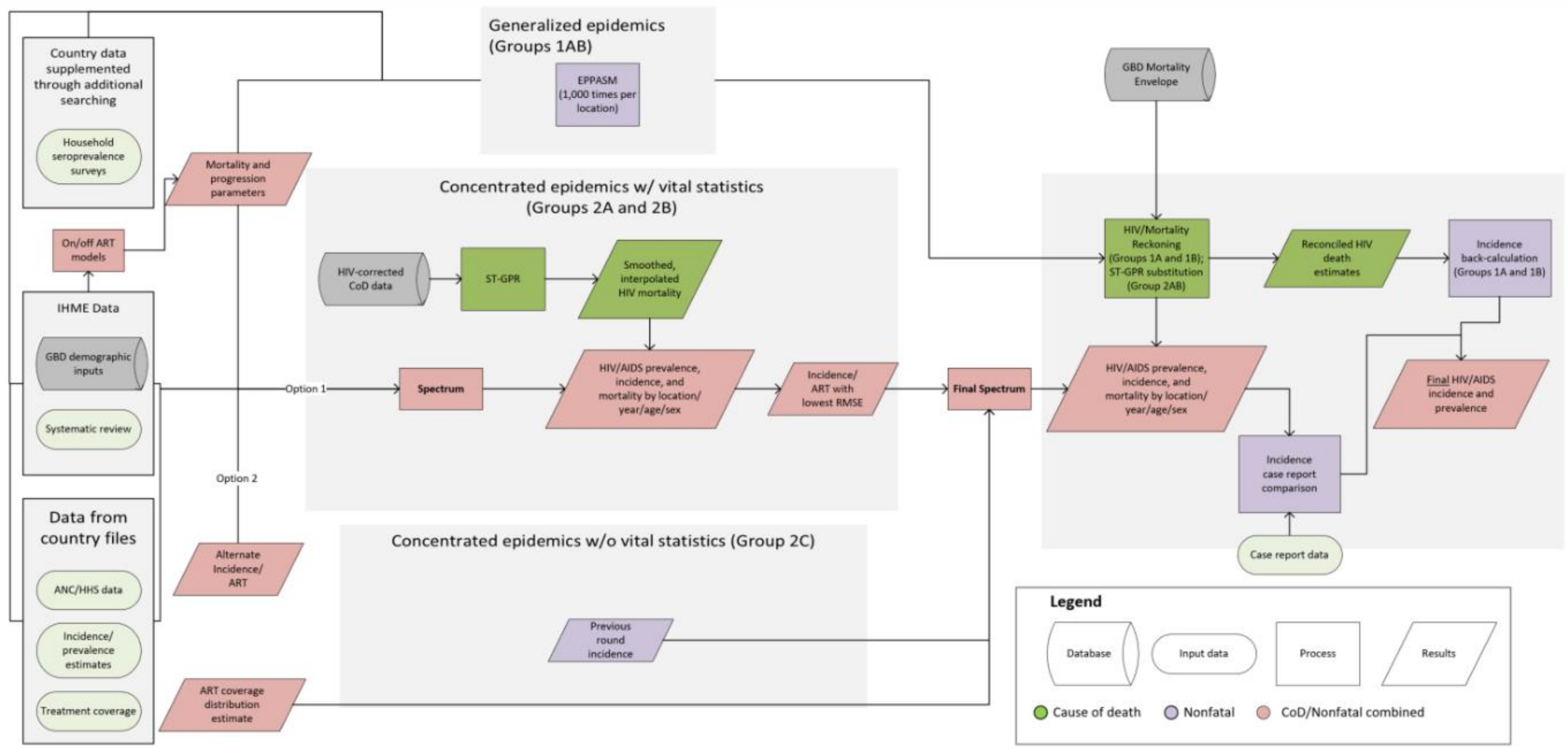

3.1. On–ART, off–ART mortality and CD4 progression parameters

3.1.1 On-ART mortality

In GBD 2021, we replaced the use of DisMod-MR[3] in favour of the meta-regression—Bayesian, regularised, trimmed (MR-BRT) model.[3] This model is a mixed effects meta-regression that accounts for between-study heterogeneity and bias. We ran a total of 90 models to arrive at our final on-ART mortality results: one for each age group (15-24, 25-34, 35-44, 45-54, or 55-100 years), sex (male or female), duration since ART initiation (0–6, 7–12 or 13–24 months) and super-region (sub-Saharan Africa, high-income, or other) strata.

We corrected reported probabilities of death for loss to follow-up using an approach developed by Verguet and colleagues.[9] Verguet and colleagues used tracing and follow-up studies to empirically estimate the relationship between death in LTFU and the rate of LTFU.

To create estimates of sex-specific hazard ratios, we use the *metan* function in Stata to create estimates of relative risks separately by region, using female age groups as the reference group within each age strata. The age and sex hazard ratios were applied to the study-level mortality rates, accounting for the distribution of ages and sexes in the mortality data. We then subtracted HIV-free mortality from the model life table process to calculate study-level age-sex HIV-specific mortality.

We then used MR-BRT to synthesise the age-sex-split study-level data into estimates of conditional probability of death over initial CD4 count. We replaced our on-ART mortality rates with those estimated off treatment if they were higher. Prior to analyzing the data, we apply a logit transformation of the outcome variable, which is the conditional probability of mortality after individuals initiate treatment with ART. This transformation, along with the corresponding variance transformation, enables the data to be analyzed using a powerful Gaussian mixed effects model (MR-BRT), with random (Gaussian) effects by study. The random errors in our model are assumed to follow a normal distribution. The link function used in our meta-regression model is the logit function. The random-effect term in our model accounts for between-study variability. This term is assumed to follow a normal distribution, capturing the heterogeneity across different studies included in the meta-analysis.

We estimate mortality for each region in its own DisMod model based on data from the IeDEA cohort collaboration[10], and include a covariate for year as mortality among the LFTU has been found to decline in recent years.[11] Finally, in cases where on-ART rates were higher, we replaced our estimated on-ART mortality rates by rates off ART to account for progression to lower CD4 categories. This ensured individuals would not experience higher mortality when they entered treatment in Spectrum or EPP-ASM.

3.1.2. Off-ART mortality and CD4 progression

Following UNAIDS assumptions, off-ART mortality and CD4 progression is modelled as shown in the figure below.

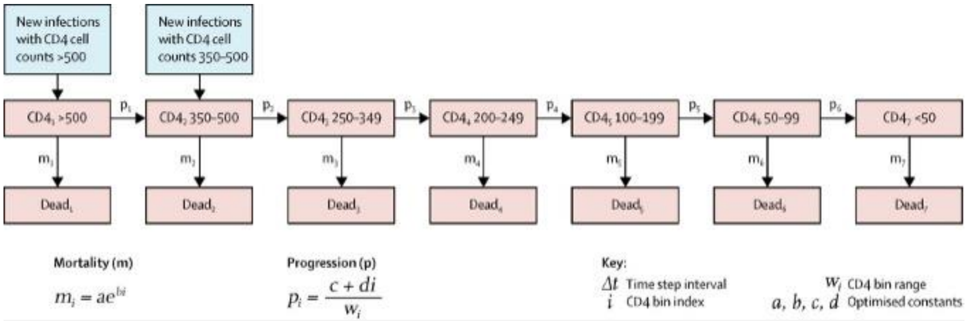

The death and progression rates between CD4 categories vary by age according to four age groups: 15–24 years, 25–34 years, 35–44 years, and 45 years or older. We modelled the logit of the conditional probability of death between years in these studies using the following formula:

$$logit(m_{ijk}) = \beta_0 + \sum_{i=1}^{4} \beta_{1i} a_i + \sum_{j=1}^{12} \beta_{2j} t_j + u_k + \epsilon_{ijk}$$

In the formula, m is conditional probability of death from year t_j to t_{j+1}, a_i is an indicator variable for age group at seroconversion (15–24 years, 25–34 years, 35–44 years, and 45 years or older), t_j is an indicator variable of year since seroconversion, and u_k is a study-level random effect. The categories of the variable t_j are one-year intervals up to 12 years since sero-conversion, after which data availability was sparse. The baseline level was the lowest category for each indicator, so 15-24 years for age at seroconversion and 0 years for years since seroconversion. The model assumed a multivariate normal distribution for the study-level random effects, u_k, and the error term, ε, after the logit transformation of the conditional probabilities of death.

By sampling the multivariate normal distribution represented by the fitted mean and variance-covariance matrix, we generated 1000 survival curves for each age group that capture the systematic variation in survival across the available studies. For each of the 1000 survival curves, we used a framework modelled after the UNAIDS optimisation framework in which we find a set of progression and death rates that minimises the sum of the squared errors for the fit to the survival curve.[12,13]

3.2. Disease model

We used two different components to derive year-, age- and sex-specific estimates of HIV incidence, prevalence, and mortality depending on locations' availability of data and extent of HIV burden, as described below:

1. EPP-ASM was used to estimate incidence, prevalence, and mortality that are consistent with serosurveillance data from antenatal care clinics and/or prevalence surveys.
2. Spectrum is a compartmental HIV progression model used to generate age-sex-specific incidence, prevalence, and death rates from input incidence and prevalence curves and assumptions about intervention scale-up and local variation in epidemiology. This model was used in conjunction with EPP-ASM for India and for all Group 2 countries.

3.2.1 Group 1 - EPP–ASM

53 countries – as well as subnational locations in India, Kenya, Ethiopia, Nigeria, and South Africa – were included in Group 1 with available ANC data and/or at least one geographically representative HIV seroprevalence survey. For all these locations we used EPP-ASM, which was updated to incorporate the new ANC bias adjustment.

In EPP-ASM[5], the transmission rate, r(t), is a simple transmission model applied at each time step (1/10 of a year) to the population. 'r' represents the number of new cases expected to emanate from a single case. Over 3000 iterations, a new r(t) is drawn, the full epidemic is determined and compared to the observed prevalence data to determine its likelihood. Beyond the end of the data, a prior distribution on r(t) helps to determine how we should expect the epidemic to behave. This assumption was different in EPP-ASM versus EPP. In EPP-ASM in most countries, we extended a random walk into the future based on the 'r-hybrid' r(t). The r-hybrid assumes a logistic decay until the year 2003, a linear interpolation until year 2008, and a random walk form after this.

For GBD 2021, we continued to use our modified version of EPP-ASM both to improve the fit to data and to generate paediatric estimates. We built a paediatric module in EPP-ASM that mirrored early updates to the paediatric module in Spectrum.[4] This child module included CD4 progression and CD4-specific mortality rates taken from a model fit to survival data from IeDEA and child initiation of ART based on ART distribution data from IeDEA. Perinatal and breastfeeding transmission was calculated as a function of prevalence among pregnant women and PMTCT programme data. We were thus able to utilise EPP-ASM to produce HIV incidence, prevalence, and mortality estimates for all ages. Additionally, we improved fit to prevalence data through allowing flexibility in the age distribution of incidence over time. We parameterised the ratio of incidence among ages 15–24:25+ as a constant before year 2000 and a linear regression thereafter. This allowed for the shifts in the age distribution of incidence observed over the course of the HIV epidemic to be reflected in our results. Finally, we utilised GBD demographic inputs and substituted in our own assumptions about HIV progression rates and on/off-ART mortality.

To incorporate uncertainty in our demographic and progression parameters, we run EPP-ASM with separate draws of CD4 progression, on- and off-ART mortality rates, fertility, and HIV-free mortality. This process produced 1000 posterior distributions for each of the locations that make up Group 1. For every location in the group, we sampled one draw from each of the sets of EPP-ASM results to create a final distribution. By sampling one draw from each set, we ensured that the distribution of mortality parameters dictating the relationship between incidence and prevalence aligned with those used in the GBD demographics estimates.

We also continued to use the approach implemented in GBD 2019[1] to address selection bias resulting from temporal and geographical variation in ANC reporting. The ANC data which EPP-ASM uses cannot be assumed as representative of HIV prevalence in the full population. This is especially the case when there are minimal or no nationally representative prevalence surveys to anchor estimates, as in the early epidemic.[14]

EPP-ASM has embedded approaches to adjust for the bias associated with using prevalence among ANC-site-attending pregnant women to estimate prevalence among the both-sexes population. For the bias between pregnant women and the national both-sexes population, it makes assumptions around the difference in total fertility rate among HIV-positive and HIV-negative women, and the difference in prevalence between men and women. For the bias associated with the data coming from ANC sites, the

specification of the likelihood of observed ANC data includes random intercepts for each clinic. The random intercepts allow each site's baseline prevalence to vary randomly around the overall mean prevalence. In other words, factors that could drive differences between sites' HIV prevalence levels are "adjusted" for.

However, the embedded approach does not explicitly account for the fact that the location of the clinic in space may also drive its HIV prevalence level. For example, we might expect rural sites to be more correlated than urban sites. Thus, to further adjust for this bias, we used an offset term that represents the difference in the prevalence among the national, both-sexes population and the prevalence among the female, pregnant population associated with an ANC site location. The offset term was derived for each location as the difference between the adjusted prevalence in a given site-year and the adjusted national prevalence in that year. These estimates are adjusted for covariates that are thought to influence prevalence, for example, access to health-care facilities, malaria incidence, and male circumcision.

Thus, our final strategy for estimating the likelihood of the observed ANC data was:

$$\boldsymbol{W_{st} = \varphi^{-1}(\rho_t) + \vartheta_{st} + u_s + e_{st}}$$

$$\boldsymbol{e_{st} \sim N(0, \sigma_{st}^2)}$$

$$\boldsymbol{u_s \sim N(0, \sigma_s^2)}$$

Where:
W_{st} = the probit transformed prevalence at site *s* and time *t*
ρ_t= The national prevalence adjusted to represent prevalence among pregnant women from the model simulation
ϑ_{st} = The offset term representing the difference between the adjusted prevalence in a given site–year and the adjusted national prevalence in that year
φ^{-1} = probit transformation
e_{st} = Site–specific error term
u_s = Site specific intercept

3.2.2. Group 2 - Spectrum

For GBD 2013, we created an exact replica of Spectrum in Python. This enabled us to run thousands of iterations of the model at once on our computing cluster and allowed for more flexible input data structures. Additionally, we scaled all input values by a uniformly sampled factor between 0.9 and 1.1 to generate estimates with realistic ranges of uncertainty. For example, if treatment retention rates across CD4 categories were 0.906, 0.759, 0.787, 0.795, 0.785, 0.756, 0.813, and 0.700, we multiplied each number by an array of equivalent size that contained factors ranging from 0.9 to 1.1. At each draw, the array would contain different, randomly selected factors in the same range. Further, we previously improved our sex-specific modelling strategy in Spectrum by sex-splitting incidence based on a model fit to the sex ratio of prevalence observed in countries with representative surveys and updated the Spectrum paediatric module to reflect changes made by UNAIDS.[13] Our child module was revised to include CD4 progression and CD4-specific mortality rates taken from a model fit to survival data from IeDEA. Finally, we updated child initiation of ART to include data on ART distribution from IeDEA. These changes were retained in GBD 2021.

ART coverage distribution

Spectrum determines the number of people initiating ART treatment across each CD4 category based on eligibility criteria, and the number of expected deaths and untreated people. In other words, groups with a large proportion of people living with HIV and high numbers of expected deaths initiated the most individuals into treatment.

We improved the basis for this distribution using survey microdata and country-level wealth information. Three relevant surveys were available: Uganda AIS 2011 and Kenya AIS 2007 and 2012. These surveys conducted CD4 count measurements and include a question regarding the amount of time that an individual receiving ART had been enrolled in treatment. Survey data provide cross-sectional CD4 count information; however, the Spectrum modelling framework tracks individuals by categorical CD4 count at the initiation of treatment. In order to crosswalk the cross-sectional survey data into estimates of CD4 count at treatment initiation, we built a model using relevant cohort data which tracked changes in CD4 count after initiation of treatment to translate an individual's current CD4 count and duration on treatment into CD4 count at initiation of treatment. The functional form for changes in CD4 count as a function of duration on treatment was a natural spline on duration with knots at 3, 12, 24, and 36 months, and an interaction between initial CD4 count and duration.

After crosswalking, we predicted the probability of being on treatment as a function of individual income (measured through an asset-based index), stratified by CD4 count, age, and sex. The results of this prediction were translated into country-specific age-sex-year-CD4 count probabilities of coverage using a conversion factor between individual income and lag-distributed GDP per capita. We used stochastic frontier analysis to constrain the maximum possible coverage for a given degree of income and CD4 count.

Predicted probabilities of coverage were input to Spectrum to inform the distribution, and not the overall level, of ART treatment by CD4 count. Within Spectrum, the probabilities of coverage are converted to counts of expected individuals on treatment in each CD4 count group. These are scaled to the distribution across CD4 count groups to match the input data on the number of people on ART coming from UNAIDS country files. In cases where the predicted number of individuals initiating treatment exceeds the total number of untreated individuals in a CD4 count group, we reallocate treatment evenly to other CD4 count groups.

3.2.3. Group 2 – Countries Without Survey Data and Vital Registration Data

32 countries had neither geographically representative seroprevalence surveys nor reliable vital registration systems, these make up group 2C locations. To produce estimates of HIV burden in these countries, we used Spectrum to produce estimates of burden. As above, the estimates of incidence, prevalence, and mortality were incorporated into the rest of the machinery via the reckoning process.

Changes in GBD 2021

For India, we used EPP-ASM to model HIV burden for GBD 2021. For India, we used EPP-ASM in combination with Spectrum, to be able to capitalise on SRS data. We also used an 'equilibrium prior,' for r(t) rather than 'r-hybrid', which provided a better fit to the comparatively lower magnitude of the epidemic. The equilibrium prior extends into the future with a rate of change following a normal distribution with a mean equal to the value of r expected when the proportion of the population

infected is saturated, ie, the epidemic has stabilised. Group 2C countries no longer sampled bias adjustment ratios from other Group 2 countries within the same super-region.

3.3. Spatiotemporal Gaussian Process Regression (ST–GPR)

Countries with vital registration data (Groups 2A and 2B)

Vital registration is one of the highest-quality sources of data on HIV burden in many countries, so generating estimates that are consistent with these data with necessary adjustment to account for any potential underreporting is critical. We identified 121 countries – as well as 760 subnational locations from China, Japan, Indonesia, India, Mexico, Sweden, the Philippines, Poland, Italy, the UK, Ukraine, Russia, New Zealand, Iran, Norway, and the USA – with usable points of vital registration data, verbal autopsy (VA) data, or sample registration system (SRS) data. In India, Vietnam, and Indonesia, we used SRS and VA data, respectively, as input mortality for CIBA. For India, we extracted the resulting age-sex distribution of incidence but scaled the level to match the adult incidence rate estimated from EPP for each state.

We estimated full time series for HIV deaths using ST-GPR fit to available cause of death data. We analysed mortality trends using ST-GPR starting in 1981, the year that HIV was first identified in the USA.[15] For ST-GPR, we adjusted the lambda (time weight) and GPR scale according to the completeness of vital registration data, with 4- and 5-star quality vital registration using parameters designed to follow the data more closely. We produced separate splines by country/age group, up to the peak year of death rate. We then ran a linear regression with fixed effects on region, age, and sex. Following this, we ran space-time residual smoothing, in which time, age, and space weights are used to inform smoothing of the residuals between datapoints and the linear regression estimate. From this process, we generated space-time estimates with the applied weights, along with the median absolute deviation (MAD) of the space-time estimates from the data. The MAD was calculated at various levels of the geographical hierarchy (eg, subnational and national), and was added into the data variance term. The data variance and space-time estimates were then analysed using Gaussian process regression to return a final estimate of mortality along with uncertainty. ST-GPR deaths were used as final deaths in group 2A and group 2B.

Although Spectrum produces HIV mortality estimates that are within the realm of possibility in most countries using the incidence curves provided in the UNAIDS country files, it is a deterministic model that has not yet been integrated into an optimisable framework. Therefore, in order to “fit” it to vital registration data, we need to adjust input incidence. In contrast to GBD 2019 and previous cycles, in addition to adjusting input incidence, we determined the most plausible best treatment input based on fit to vital registration as well.

3.4. Additional Adjustments

Additional adjustments enabled us to use case surveillance data and HIV mortality estimated as part of the GBD all-cause mortality life table process. In countries and territories with high-quality case notification data, we scaled incidence results to align with case reports after accounting for an assumed average of five years’ lag to diagnosis. For Group 1 countries and territories, we used an ensemble approach to reconcile the differences between HIV mortality estimated by EPP-ASM and by HIV mortality estimated as a part of the GBD all-cause mortality life table process to generate final HIV mortality. For ages 15 and over, the ensemble model averaged HIV mortality estimates from the two

processes with equal weights. For under age 15, we applied the fraction of deaths due to HIV in Spectrum to estimate all-cause mortality to generate HIV-specific mortality and mortality from all other causes (HIV-free mortality).

The HIV/mortality reckoning process is intended as a method of reconciling separate estimates of HIV mortality (and its resulting effect on estimates of HIV-free and all-cause mortality) in Group 1 countries by averaging estimates of HIV mortality from the model life table process and EPP-ASM. Additional details on the reckoning can be found elsewhere.[3]

Since EPP-ASM produces HIV incidence, prevalence, and deaths that are consistent with one another over time, the reckoning process results in death numbers that are no longer consistent with the incidence and prevalence produced in EPP-ASM. To recreate this consistency, we recalculated incidence for all Group 1 locations using reckoned deaths and deaths produced by EPP-ASM. The updated incidence is calculated by aggregating counts of new infections, HIV deaths from EPP-ASM, and HIV deaths after reckoning at the year-sex level. The difference between reckoned HIV deaths and HIV deaths from EPP-ASM is added to EPP-ASM incidence, and we calculate the ratio between updated incidence and EPP-ASM incidence. Age-specific counts of new infections are then scaled by their corresponding sex-year ratios.

Changes for GBD 2021
For GBD 2021, we created a grid of incidence and treatment options and reran Spectrum for each location using each of these options, rather than using the CIBA-adjusted incidence for our final Spectrum run in all locations. The incidence options included the CIBA-adjusted incidence and the non-CIBA-adjusted incidence from the initial Spectrum run, both using the most recent data and the last cycle, in addition to incidence data available from public-use UNAIDS files. The adult ART options included the data available from public-use UNAIDS files. Where these data were provided in terms of the number of people on treatment, we created additional treatment options by dividing the number on treatment by prevalence, as estimated by the current and previous GBD cycles. We ran Spectrum on every combination of incidence and treatment options and determined the root mean squared error of the resulting mortality relative to the vital registration data.

Finally, to produce location-, year-, age-, and sex-specific estimates of HIV incidence, prevalence, and mortality, we ran a final Spectrum run using the incidence and treatment option that resulted in the best fit to vital registration data, or the lowest RMSE.

Section 4. Forecasting HIV

4.1. Forecasting ART Coverage

In the past, ART coverage is projected using the spending on HIV care and treatment and ART price[2]. However, we found that some locations maintained flat coverage at lower levels into the future. We interrogated our ART forecasting methods to determine the reason. We started by assessing the capping system and the strength of the relationship between coverage and GDP/ART dose equivalents. ART dose was calculated using Spend/ART price, and we discovered that the Spending in some locations is zero.

To avoid using spending and to address the problem that ART projections maintain flat in the future, we projected the ART coverage using historical rate of change (ROC). The cap is 0.95 for all locations, CD4 counts, ages, and both sexes. We calculated ROCs using ART coverage from past 5 years by age, sex, and

CD4 count. After projection, we found out that in the same age group and sex combination, the future trend of ART coverage by CD4 count differs. We wanted to force the future trend of ART coverage to be the same across CD4 count groups. So, we selected the largest ROC across all CD4 groups in each age-sex combination and apply this ROC to all CD4 groups. By doing so, the projected ART coverage has the same future trend in each age-sex combination.

4.2. Forecasting the Transmission rate

We explored various approaches to calculating and forecasting using the transmission rate. A few challenges came up related to differences when incidence is calculated within EPP-ASM compared to Spectrum. For this purpose transmission rate = incidence hazard / prevalence.

In EPP-ASM, incidence is calculated at the beginning of each time loop, while in Spectrum it is at the end. This affected both the back-calculation of the "true" transmission rate and the forward calculation of incidence with the forecasted transmission rate. In the end, we realized that we were not interested in back-calculating the true transmission rate, but the transmission rate that would reproduce new infections in Spectrum, the simulation we are currently using for forecasting. We then switched the input incidence for the transmission rate calculation from Spectrum/EPPASM output incidence to final GBD incidence.

Using the transmission rate in Spectrum required us to augment the code to calculate new infections using the transmission rate, prevalence, susceptible population, and ART coverage. The first year of the transmission rate based calculation requires an epidemic seed, given that there are zero PLHIV to infect others. The parameter name used in the code is *iota*, and it operates just like incidence hazard (multiplied by the susceptible population). We included back-calculation of *iota* in the transmission rate calculation and set up Spectrum to pull the iota value from that file and use it to kick off the epidemic.

After back-calculating the transmission rates and plotting them by region, we observed that the transmission rate generally exhibits a high starting value followed by a logistic decline, with random variation over the past ~15 years. This follows the r-hybrid functional form used to parameterize the time-trend for the transmission rate in EPP-ASM. R-hybrid combines a logistic function with a linear piecewise spline with a random-walk penalty. In general, high-income locations exhibited higher transmission rates (>0.3), and lower-income locations lower transmission rates (<0.3). We chose to use a simple global approach to forecasting after trying various super region-specific approaches and encountering issues of "runaway" epidemics (more to say about this below). We took the global median across all location-year values of the transmission rate in the last ten years (choosing this for its relative stability in contrast to the high variation in earlier periods) to get a global equilibrium transmission rate value to use as a target to converge towards over time.

The choice of a global equilibrium value followed experimentation where we used superregion equilibrium values. In higher income locations, we saw many forecasts that resulted in what we are calling "runaway" epidemics, where the effective reproductive rate is above one even in the presence of very high ART coverage and prevalence grows to greater than ten percent. This seems to partially be a result of the fact that the *entire* population is considered as potentially susceptible in these locations, even though the epidemics are dominated by higher-risk groups like injections drug users, sex workers, and men who have sex with men. Taking into consideration the true size of the susceptible population would be one approach to constraining new infections in the future, but this would represent a substantial change in methodology and would require some difficult calculations of the proportion of the population within in each high-risk group. This approach of estimating the size of high-risk groups

and modeling them separately is taken in a few concentrated epidemics in Spectrum, but we are not clear on the quality of the estimation of the population sizes, nor the data available to support estimation. Instead, we can control the transmission rate and ART coverage. Therefore, we took a conservative approach and forecasted global convergence towards a lower global equilibrium and sought to increase ART coverage into the future.

To forecast location-specific trends in the transmission rate, we projected the location-specific trend over the last five years and took a shifting weighted average between that projection and the global equilibrium. We used a logistic weighting scheme that smoothly moved from fully weighting the location-specific projection to fully weighting the global equilibrium over a twenty-year window.

We averaged the location-specific projections with the global equilibrium value only in locations with increasing trend and where the projected transmission rate is larger than the global equilibrium value. If the location-specific projections decrease, even if the location-specific projections are higher than global equilibrium, it won't cause "runaway". And if the increasing location-specific projections are lower than global equilibrium in the twenty-year averaging window, there is no need to drag the projections up to global equilibrium. We cap the projection using the value 1.25 times transmission rate at extension year: 2021.

4.3. Projections of HIV incidence, prevalence, and mortality

In order to produce age- and sex-specific estimates of HIV incidence, prevalence, and mortality, we input projected transmission rates along with ART, PMTCT, and Cotrimoxazole coverage, as well as a number of other predicted demographic inputs, into the Spectrum model. Spectrum is a cohort component model originally developed by UNAIDs that we have modified to incorporate CD4-specific probability of treatment in addition to a number of other methods developments made for GBD.[1] Spectrum ages a population over time using demographic parameters while applying HIV incidence, disease progression, treatment coverage, and mortality. Our final results are age-,sex-, location-specific Spectrum outputs through 2100.

Section 5. Key Metrics

5.1. Prevalence of Unsuppressed Viremia

For each location time point the PUV is calculated with the following equation, where each variable is location and time specific:

PUV = (untreated + treated but not virally suppressed) / population

= ((PLHIV * (1 – ART Coverage) + (1-vir_sup)*PLHIV * ART Coverage) / population

Where PLHIV is the count of people living with HIV, ART coverage is the proportion of PLHIV who are receiving ART, vir_sup is the proportion of people on ART who are virally suppressed, and population is the total population.

As an example of PUV calculation, consider a 100-person population with 10 percent HIV prevalence, 80 percent ART coverage, and 90 percent of people on ART are virally suppressed. We calculate PUV by taking the total PHIV (10 people) and splitting them into off-treatment PLHIV (2 people) and PLHIV on treatment who have unsuppressed viremia ((1 – 0.9) * 8 people = 0.8 people), leading to a total of 2.8

people with unsuppressed viremia. This turned into a prevalence by either dividing by the total population (2.8 / 100 = 2.8 % PUV).

5.2. Lifetime Probability of Acquiring HIV

Equations for calculation of life time probability of acquiring HIV

Let $\mu_i$be the HIV-free mortality rate and β_i be the incidence rate in age group i. The sum of the two rates is used to calculate the probability of exiting the susceptible population during age group i:

$$P(\text{exit}_i) = 1 - \exp\left(-(\mu_i + \beta_i) \cdot n_i\right)$$

where n_i is the size of age group i.

We then calculate the proportion of those you enter age group i who acquire HIV during age group i:

$$P(\text{HIV}_i) = P(\text{exit}_i) \cdot \frac{\beta_i}{\beta_i + \mu_i}$$

We then calculate the proportion of the initial cohort acquiring HIV during age group i:

$$H_i = S_i \cdot P(\text{HIV}_i)$$

Finally, the lifetime risk of HIV is calculated in a lifetable fashion, iteratively calculating $P(\text{HIV}_i)$ and the remaining susceptible group, S_i, according to the following algorithm.

<u>Lifetime risk of HIV acquisition algorithm</u>

Inputs:

$\boldsymbol{\mu}$, Period age-specific HIV-free mortality rates
$\boldsymbol{\beta}$, Period age-specific HIV incidence rates
$\boldsymbol{n}$, Age group sizes

Algorithm:

Set S_1 equal to 1, the size of the initial susceptible cohort
For i in the number of age groups:
Calculate the probability of exiting: $P(\text{exit}_i) = 1 - \exp\left(-(\mu_i + \beta_i) \cdot n_i\right)$
Calculate the proportion of exits that are HIV acquisition: $P(\text{HIV}_i) = P(\text{exit}_i) \cdot \frac{\beta_i}{\beta_i + \mu_i}$
Calculate the proportion of the initial cohort that acquired HIV: $H_i = S_i \cdot P(\text{HIV}_i)$

Calculate the proportion of the initial cohort still susceptible: $S_{i+1} = S_i \cdot (1 - P(\mathrm{exit}_i)$

Sum over all H_i to get the cumulative lifetime risk of HIV acquisition

Section 6. Statement of GATHER compliance

Checklist of information that should be included in reports of global health estimates, with description of compliance and location of information the current study

#	GATHER checklist item	Description of compliance	Reference
**Objectives and funding**			
1	Define the indicator(s), populations (including age, sex, and geographic entities), and time period(s) for which estimates were made.	Narrative provided in paper and appendix describing indicators, definitions, populations, and time periods	Main text (Methods) and Appendix (Methods)
2	List the funding sources for the work.	Funding sources listed in paper	Summary (Funding)
**Data Inputs**			
For all data inputs from multiple sources that are synthesized as part of the study:			
3	Describe how the data were identified and how the data were accessed.	Narrative description of data seeking methods provided	Main text (Methods) and Appendix (Methods)
4	Specify the inclusion and exclusion criteria. Identify all ad-hoc exclusions.	Narrative about inclusion and exclusion criteria provided; ad hoc exclusions in appendix supplementary methods	Main text (Methods) and Appendix (Methods)
5	Provide information on all included data sources and their main characteristics. For each data source used, report reference information or contact name/institution, population represented, data collection method, year(s) of data collection, sex and age range, diagnostic criteria or measurement method, and sample size, as relevant.	An interactive, online data source tool that provides metadata for data sources by component, geography, cause, risk, or impairment has been developed, and data source citations provided	Appendix (Methods) with additional information about these sources available at https://ghdx.healthdata.org/gbd-2021 And https://ghdx.healthdata.org/record/ihme-data/global-life-expectancy-all-cause-mortality-and-cause-specific-mortality-forecasts-2022-2050 And https://ghdx.healthdata.org/record/ihme-data/gbd-2021-hiv-acquisition-viraemia-1990-2021
6	Identify and describe any categories of input data that have potentially important biases (e.g., based on characteristics listed in item 5).	Summary of known biases included in appendix supplementary methods	Appendix (Methods)
For data inputs that contribute to the analysis but were not synthesized as part of the study:			
7	Describe and give sources for any other data inputs.	Included in online data source tool	Global Health Data Exchange: https://ghdx.healthdata.org/gbd-2021 And https://ghdx.healthdata.org/record/ihme-data/global-life-expectancy-all-cause-mortality-and-cause-specific-mortality-forecasts-2022-2050 And https://ghdx.healthdata.org/record/ihme-data/gbd-2021-hiv-acquisition-viraemia-1990-2021
For all data inputs:			
8	Provide all data inputs in a file format from which data can be efficiently extracted (e.g., a spreadsheet rather than a PDF), including all relevant meta-data listed in item 5. For any data inputs that cannot be shared because of ethical or legal reasons, such as third-party ownership, provide a contact name or the name of the institution that retains the right to the data.	Downloads of input data available through online data tools; input data not available in tools will be made available upon request	Global Health Data Exchange:https://ghdx.healthdata.org/gbd-2021 And https://ghdx.healthdata.org/record/ihme-data/global-life-expectancy-all-cause-mortality-and-cause-specific-mortality-forecasts-2022-2050 And

			https://ghdx.healthdata.org/record/ihme-data/gbd-2021-hiv-acquisition-viraemia-1990-2021
**Data analysis**			
**9**	Provide a conceptual overview of the data analysis method. A diagram may be helpful.	Flow diagram of methodological process provided, as well as narrative descriptions of modelling process	Main text (Methods) and Appendix (Methods)
**10**	Provide a detailed description of all steps of the analysis, including mathematical formulae. This description should cover, as relevant, data cleaning, data pre-processing, data adjustments and weighting of data sources, and mathematical or statistical model(s).	Flow diagram and detailed methods write-up covering all data extraction, processing, and modelling processes provided	Main text (Methods) and Appendix (Methods)
**11**	Describe how candidate models were evaluated and how the final model(s) were selected.	Provided in methodological write-up	Appendix (Methods)
**12**	Provide the results of an evaluation of model performance, if done, as well as the results of any relevant sensitivity analysis.	Provided in methodological write-up	Appendix (Methods)
**13**	Describe methods for calculating uncertainty of the estimates. State which sources of uncertainty were, and were not, accounted for in the uncertainty analysis.	Provided in main text methods narrative description and appendix methodological writeup	Main text (Methods) and Appendix (Methods)
**14**	State how analytic or statistical source code used to generate estimates can be accessed.	Remote code repository for access to analytic code provided	Remote code repository https://ghdx.healthdata.org/gbd-2021/code
**Results and Discussion**			
**15**	Provide published estimates in a file format from which data can be efficiently extracted.	Tables in appendices and online results tool	Appendix Results and https://ghdx.healthdata.org/gbd-2021 And https://ghdx.healthdata.org/record/ihme-data/global-life-expectancy-all-cause-mortality-and-cause-specific-mortality-forecasts-2022-2050 And https://ghdx.healthdata.org/record/ihme-data/gbd-2021-hiv-acquisition-viraemia-1990-2021
**16**	Report a quantitative measure of the uncertainty of the estimates (e.g. uncertainty intervals).	Uncertainty provided with all results	Main text (Results), Appendix Results
**17**	Interpret results in light of existing evidence. If updating a previous set of estimates, describe the reasons for changes in estimates.	Discussion of results and methodological changes between GBD rounds provided in manuscript narrative and appendix	Main text (Methods, Results and Discussion) and Appendix (Methods)
**18**	Discuss limitations of the estimates. Include a discussion of any modelling assumptions or data limitations that affect interpretation of the estimates.	Discussion of limitations, including modelling assumptions and data limitations, included in manuscript narrative and appendix	Main text (Methods and Discussion) and Appendix (Methods)

Section 7. References

1. Jahagirdar D, Walters MK, Novotney A, Brewer ED, Frank TD, Carter A, et al. Global, regional, and national sex-specific burden and control of the HIV epidemic, 1990–2019, for 204 countries and territories: the Global Burden of Diseases Study 2019. Lancet HIV. 2021 Oct;8(10):e633–51.

2. Frank TD, Carter A, Jahagirdar D, Biehl MH, Douwes-Schultz D, Larson SL, et al. Global, regional, and national incidence, prevalence, and mortality of HIV, 1980–2017, and forecasts to 2030, for 195 countries and territories: a systematic analysis for the Global Burden of Diseases, Injuries, and Risk Factors Study 2017. Lancet HIV. 2019 Dec 1;6(12):e831–59.

3. Vos T, Lim SS, Abbafati C, Abbas KM, Abbasi M, Abbasifard M, et al. Global burden of 369 diseases and injuries in 204 countries and territories, 1990–2019: a systematic analysis for the Global Burden of Disease Study 2019. The Lancet. 2020 Oct 17;396(10258):1204–22.

4. Stover J, Glaubius R, Mofenson L, Dugdale CM, Davies MA, Patten G, et al. Updates to the Spectrum/AIM model for estimating key HIV indicators at national and subnational levels. AIDS Lond Engl. 2019 Dec 15;33(Suppl 3):S227–34.

5. Eaton JW, Brown T, Puckett R, Glaubius R, Mutai K, Bao L, et al. The Estimation and Projection Package Age-Sex Model and the r-hybrid model: new tools for estimating HIV incidence trends in sub-Saharan Africa. AIDS Lond Engl. 2019 Dec 15;33(Suppl 3):S235–44.

6. Dwyer-Lindgren L, Cork MA, Sligar A, Steuben KM, Wilson KF, Provost NR, et al. Mapping HIV prevalence in sub-Saharan Africa between 2000 and 2017. Nature. 2019;570(7760):189–93.

7. Kyu HH, Jahagirdar D, Cunningham M, Walters M, Brewer E, Novotney A, et al. Accounting for misclassified and unknown cause of death data in vital registration systems for estimating trends in HIV mortality. J Int AIDS Soc. 2021 Sep 21;24(Suppl 5):e25791.

8. Murray CJL, Ortblad KF, Guinovart C, Lim SS, Wolock TM, Roberts DA, et al. Global, regional, and national incidence and mortality for HIV, tuberculosis, and malaria during 1990–2013: a systematic analysis for the Global Burden of Disease Study 2013. The Lancet. 2014 Sep 13;384(9947):1005–70.

9. Verguet S, Lim SS, Murray CJL, Gakidou E, Salomon JA. Incorporating Loss to Follow-up in Estimates of Survival Among HIV-Infected Individuals in Sub-Saharan Africa Enrolled in Antiretroviral Therapy Programs. J Infect Dis. 2013 Jan 1;207(1):72–9.

10. Tarwater PM, Margolick JB, Jin J, Phair JP, Detels R, Rinaldo C, et al. Increase and plateau of CD4 T-cell counts in the 3 1/2 years after initiation of potent antiretroviral therapy. J Acquir Immune Defic Syndr. 2001 Jun 1;27(2):168–75.

11. Zhou J, Kumarasamy N, Ditangco R, Kamarulzaman A, Lee CKC, Li PCK, et al. The TREAT Asia HIV Observational Database. J Acquir Immune Defic Syndr 1999. 2005 Feb;38(2):174–9.

12. Ghys PD, Żaba B, Prins M. Survival and mortality of people infected with HIV in low and middle income countries: results from the extended ALPHA network. AIDS Lond Engl. 2007 Nov;21(Suppl 6):S1–4.

13. Hallett TB, Zaba B, Todd J, Lopman B, Mwita W, Biraro S, et al. Estimating Incidence from Prevalence in Generalised HIV Epidemics: Methods and Validation. PLoS Med. 2008 Apr;5(4):e80.

14. Ng M, Gakidou E, Murray CJ, Lim SS. A comparison of missing data procedures for addressing selection bias in HIV sentinel surveillance data. Popul Health Metr. 2013 Jul 24;11:12.

15. Pneumocystis Pneumonia --- Los Angeles [Internet]. [cited 2024 Feb 12]. Available from: https://www.cdc.gov/mmwr/preview/mmwrhtml/june_5.htm
