# Supplementary material for: Global, regional, and national burden of HIV/AIDS, 1990–2021, and forecasts to 2050, for 204 countries and territories: the Global Burden of Disease Study 2021
Source: Lancet HIV. 2024 Nov 25;11(12):e807–22. doi: 10.1016/S2352-3018(24)00212-1 (PMC11612058; doi:10.1016/S2352-3018(24)00212-1)
Supplement: Supplementary appendix 3 [file mmc3.pdf]

THE LANCET
HIV

Supplementary appendix 3

This appendix formed part of the original submission and has been peer reviewed. We post it as supplied by the authors.

Supplement to: GBD 2021 HIV Collaborators. Global, regional, and national burden of HIV/AIDS, 1990–2021, and forecasts to 2050, for 204 countries and territories: the Global Burden of Disease Study 2021. *Lancet HIV* 2024; published online Nov 25. https://doi.org/10.1016/S2352-3018(24)00212-1.

Appendix 3: Authorship appendix to “Global, regional, and national burden of HIV/AIDS, 1990–2021, and forecasts to 2050, for 204 countries and territories: the Global Burden of Disease Study 2021”

This appendix provides further authorship detail for “Global, regional, and national burden of HIV/AIDS, 1990–2021, and forecasts to 2050, for 204 countries and territories: the Global Burden of Disease Study 2021”

Table of Contents

GBD 2021 HIV/AIDS Collaborators ... 2

Affiliations ... 5

Authors’ Contributions ... 23

Managing the overall research enterprise ... 23

Writing the first draft of the manuscript ... 23

Primary responsibility for applying analytical methods to produce estimates ... 23

Primary responsibility for seeking, cataloguing, extracting, or cleaning data; designing or coding figures and tables ... 23

Providing data or critical feedback on data sources ... 23

Developing methods or computational machinery ... 25

Providing critical feedback on methods or results ... 25

Drafting the work or revising it critically for important intellectual content ... 28

Managing the estimation or publications process ... 30

GBD 2021 HIV/AIDS Collaborators

Austin Carter*, Meixin Zhang*, Khai Hoan Tram*, Magdalene K Walters, Deepa Jahagirdar, Edmond D Brewer, Amanda Novotney, Dylan Lasher, Emmanuel A Mpolya, Avina Vongpradith, Jianing Ma, Megan Verma, Tahvi D Frank, Jiawei He, Sam Byrne, Christine Lin, Regina-Mae Villanueva Dominguez, Spencer A Pease, Haley Comfort, Erin A May, Yohannes Habtegiorgis Abate, Hedayat Abbastabar, Atef Abdelkader, Parsa Abdi, Meriem Abdoun, Jeza Muhamad Abdul Aziz, Hassan Abidi, Olumide Abiodun, Richard Gyan Aboagye, Lucas Guimarães Abreu, Yonas Derso Abtew, Eman Abu-Gharbieh, Salahdein Aburuz, Ahmed Abu-Zaid, Isaac Yeboah Addo, Oyelola A Adegboye, Victor Adekanmbi, Charles Oluwaseun Adetunji, Juliana Bunmi Adetunji, Daniel Adedayo Adeyinka, Kishor Adhikari, Qorinah Estiningtyas Sakilah Adnani, Leticia Akua Adzigbli, Fatemeh Afrashteh, Saira Afzal, Shahin Aghamiri, Feleke Doyore Agide, Antonella Agodi, Williams Agyemang-Duah, Bright Opoku Ahinkorah, Faisal Ahmad, Sajjad Ahmad, Shahzaib Ahmad, Aqeel Ahmad, Ibrar Ahmed, Haroon Ahmed, Syed Anees Ahmed, Safoora Ahmed, Ali Ahmed, Mohammed Ahmed, Ayman Ahmed, Gizachew Taddesse Akalu, Karolina Akinosoglou, Salah Al Awaidy, Hanadi Al Hamad, Amjad S Al Mosa, Omar Ali Mohammed Al Zaabi, Samer O Alalalmeh, Nazmul Alam, Noore Alam, Fahad Mashhour Alanezi, Daniel Shewaye Alayu, Mohammad T AlBataineh, Seyedeh Yasaman Alemohammad, Adel Ali Saeed Al-Gheethi, Syed Shujait Ali, Mohammed Usman Ali, Abid Ali, Liaqat Ali, Waad Ali, Akram Al-Ibraheem, Joseph Uy Almazan, Awais Altaf, Diala Altwalbeh, Nelson Alvis-Guzman, Walid Adnan Al-Zyoud, Reza Amani, Tewodros Getnet Amera, Edward Kwabena Ameyaw, Sohrab Amiri, Hubert Amu, Ganiyu Adeniyi Amusa, Abhishek Anil, Abdul-Azeez Adeyemi Anjorin, Carl Abelardo T Antonio, Saleha Anwar, Razique Anwer, Ekenedilichukwu Emmanuel Anyabolo, Anayochukwu Edward Anyasodor, Geminn Louis Carace Apostol, Ali Ardekani, er Areda, Brhane Berhe Aregawi, Abdulfatai Aremu, Keivan Armani, Mulusew A Asemahagn, Mubarek Yesse Ashemo, Tahira Ashraf, Marvellous O Asika, Haftu Asmerom Asmerom, Maha Moh'd Wahbi Atout, Avinash Aujayeb, Hamzeh Awad, Adedapo Wasiu Awotidebe, Beatriz Paulina Ayala Quintanilla, Firayad Ayele, Sina Azadnajafabad, Shahkaar Aziz, Darshan B B, Giridhara Rathnaiah Babu, Muhammad Badar, Saeed Bahramian, Abdulaziz T Bako, Wondu Feyisa Balcha, Kiran Bam, Biswajit Banik, Mainak Bardhan, Till Winfried Bärnighausen, Hiba Jawdat Barqawi, Zarrin Basharat, Hameed Akande Bashiru, Afisu Basiru, Mohammad-Mahdi Bastan, Saurav Basu, Prapthi Persis Bathini, Kavita Batra, Ravi Batra, Nebiyou Simegnew Bayleyegn, Tahmina Begum, Amir Hossein Behnoush, Maryam Beiranvand, Melaku Ashagrie Belete, Abel Cherkos Belete, Apostolos Beloukas, Alice A Beneke, Azizullah Beran, Alemshet Yirga Berhie, Amiel Nazer C Bermudez, Robert S Bernstein, Kebede A Beyene, Pankaj Bhardwaj, Nikha Bhardwaj, Ajay Nagesh Bhat, Vivek Bhat, Gurjit Kaur Bhatti, Jasvinder Singh Bhatti, Keralem Anteneh Bishaw, Khushboo D Bisht, Trupti Bodhare, Aadam Olalekan Bodunrin, Azizbek A Boltaev, Hamed Borhany, Souad Bouaoud, Colin Stewart Brown, Danilo Buonsenso, Katrin Burkart, Yasser Bustanji, Zahid A Butt, Chao Cao, Rosario Cárdenas, Muthia Cenderadewi, Joshua Chadwick, Chiranjib Chakraborty, Sandip Chakraborty, Rama Mohan Chandika, Vijay Kumar Chattu, Akhilanand Chaurasia, Guangjin Chen, Patrick R Ching, Hitesh Chopra, Sonali Gajanan Choudhari, Dinh-Toi Chu, Isaac Sunday Chukwu, Eric Chung, Zinhle Cindi, Rosa A S Couto, Natalia Cruz-Martins, Silvia Magali Cuadra-Hernández, Bashir Dabo, Omid Dadras, Gizachew Worku Dagnew, Tukur Dahiru, Xiaochen Dai, Aso Mohammad Darwesh, José das Neves, Nihar Ranjan Dash, Mohsen Dashti, Fernando Pio De la Hoz, Shayom Debopadhaya, Louisa Degenhardt, Ivan Delgado-Enciso, Kebede Deribe, Don C Des Jarlais, Hardik Dineshbhai Desai, Keshab Deuba, Amol S Dhane, Sameer Dhingra, Daniel Diaz, Michael R Diaz, Delaney D Ding, Thanh Chi Do, Sushil Dohare, Deepa Dongarwar, Wendel Mombaque dos Santos, Ojas Prakashbhai Doshi, Ashel Chelsea Dsouza, Haneil Larson Dsouza, Viola Savy Dsouza, Senbagam Duraisamy, Arkadiusz Marian Dziedzic, Alireza Ebrahimi,

Abdelaziz Ed-Dra, Hisham Atan Edinur, Ferry Efendi, Michael Ekholuenetale, Temitope Cyrus Ekundayo, Iman El Sayed, Muhammed Elhadi, Chadi Eltaha, Sharareh Eskandarieh, Majid Eslami, Ugochukwu Anthony Eze, Ayesha Fahim, Ali Fatehizadeh, Nelsensius Klau Fauk, Patrick Fazeli, Ginenus Fekadu, Nuno Ferreira, Belete Sewasew Firew, Florian Fischer, Morenike Oluwatoyin Folayan, Behzad Foroutan, Takeshi Fukumoto, Sridevi G, Muktar A Gadanya, Abhay Motiramji Gaidhane, Abduzhappar Gaipov, Aravind P Gandhi, Mohammad Arfat Ganiyani, Miglas Welay Gebregergis, Mesfin Gebrehiwot, Teferi Gebru Gebremeskel, Motuma Erena Getachew, Keyghobad Ghadiri, Afsaneh Ghasemzadeh, Ahmad Ghashghaee, Ehsan Gholami, Nasim Gholizadeh, Mahsa Ghorbani, Artyom Urievich Gil, Alem Abera Girmay, Mahaveer Golechha, Davide Golinelli, Alessandra C Goulart, Anmol Goyal, Mesay Dechasa Gudeta, Sapna Gupta, Bhawna Gupta, Awoke Derbie Habteyohannes, Dariush Haghmorad, Arvin Haj-Mirzaian, Rabih Halwani, Demelash Woldeyohannes Handiso, Zaim Anan Haq, Harapan Harapan, Arief Hargono, Ahmed I Hasaballah, Md Saquib Hasnain, Shoaib Hassan, Soheil Hassanipour, Omar E Hegazi, Mohammad Heidari, Kamal Hezam, Mbuzeleni Mbuzeleni Hlongwa, Nguyen Quoc Hoan, Praveen Hoogar, Mehdi Hosseinzadeh, Ahmad Hosseinzadeh Adli, Tsegaye Gebreyes Hundie, Kiavash Hushmandi, Hong-Han Huynh, Segun Emmanuel Ibitoye, Adalia Ikiroma, Kevin S Ikuta, Olayinka Stephen Ilesanmi, Irena M Ilic, Arnaud Iradukunda, Mustafa Alhaji Isa, Nahlah Elkudssiah Ismail, Ihoghosa Osamuyi Iyamu, Vinothini J, Kathryn H Jacobsen, Akhil Jain, Ammar Abdulrahman Jairoun, Mihajlo Jakovljevic, Manthan Dilipkumar Janodia, Amirreza Javadi Mamaghani, Alelign Tasew Jema, Mohammad Jokar, Jost B Jonas, Nitin Joseph, Charity Ehimwenma Joshua, Ali Kabir, Md. Awal Kabir, Zubair Kabir, Vidya Kadashetti, Feroze Kaliyadan, Kehinde Kazeem Kanmodi, Suthanthira Kannan S, Ibraheem M Karaye, Arman Karimi Behnagh, Molly B Kassel, Gbenga A Kayode, Himanshu Khajuria, Nauman Khalid, Anees Ahmed Khalil, Faham Khamesipour, Gulfaraz Khan, Ejaz Ahmad Khan, Yusra H Khan, Mohammad Jobair Khan, M Nuruzzaman Khan, Khaled Khatab, Feriha Fatima Khidri, Zahra Khorrami, Majid Khosravi, Jagdish Khubchandani, Min Seo Kim, Jong Yeob Kim, Yun Jin Kim, Adnan Kisa, Sezer Kisa, Somayeh Komaki, Shivakumar KM Marulasiddaiah Kondlahalli, Parvaiz A Koul, Sindhura Lakshmi Koulmane Laxminarayana, Kewal Krishan, Barthelemy Kuate Defo, Md Abdul Kuddus, Mukhtar Kulimbet, Vishnutheertha Kulkarni, Rakesh Kumar, Vijay Kumar, Nithin Kumar, Manasi Kumar, Muhammad Awwal Ladan, Dharmesh Kumar Lal, Thao Thi Thu Le, Nhi Huu Hanh Le, Seung Won Lee, Kate E LeGrand, Temesgen L Lerango, Ming-Chieh Li, Virendra S Ligade, Stephen S Lim, Liknaw Workie Limenh, Xuefeng Liu, Runben Liu, Rakesh Lodha, Arianna Maever Loreche, Hawraz Ibrahim M. Amin, Zheng Feei Ma, Azeem Majeed, Elaheh Malakan Rad, Hardeep Singh Malhotra, Kashish Malhotra, Ahmad Azam Malik, Iram Malik, Tauqeer Hussain Mallhi, Mohammad Ali Mansournia, Bishnu P Marasini, Bernardo Alfonso Martinez-Guerra, Francisco Rogerlândio Rogerlândio Martins-Melo, Miquel Martorell, Roy Rillera Marzo, Navgeet Mathur, Anna Laura W McKowen, Hadush Negash Meles, Endalkachew Belayneh Melese, Ziad Ahmed Memish, Walter Mendoza, Ritesh G Menezes, Tuomo J Meretoja, Tomislav Mestrovic, Peter Meylakhs, Laurette Mhlanga, Irmina Maria Michalek, Ana Carolina Micheletti Gomide Nogueira de Sá, Giuseppe Minervini, Le Huu Nhat Minh, Babak Moazen, Nouh Saad Mohamed, Sakineh Mohammad-Alizadeh-Charandabi, Abdollah Mohammadian-Hafshejani, Hussen Mohammed, Salahuddin Mohammed, Mustapha Mohammed, Ali H Mokdad, Lorenzo Monasta, Mohammad Ali Moni, Fateme Montazeri, Maryam Moradi, Yousef Moradi, Rohith Motappa, Vincent Mougin, Sumaira Mubarik, George Duke Mukoro, Francesk Mulita, Kavita Munjal, Yanjinlkham Munkhsaikhan, B.V. Murlimanju, Fungai Musaigwa, Ghulam Mustafa, Saravanan Muthupandian, Ahamarshan Jayaraman Nagarajan, Pirouz Naghavi, Gurudatta Naik, Firzan Nainu, Mohammad Sadeq Najafi, Shumaila Nargus, Samidi Nirasha Kumari Navaratna, Muhammad Naveed, Vinod C Nayak, Biswa Prakash Nayak, Sabina Onyinye Nduaguba, Chernet Tafere

Negesse, Mohammad Hadi Nematollahi, Georges Nguefack-Tsague, Dang H Nguyen, Hien Quang Nguyen, Van Thanh Nguyen, Robina Khan Niazi, Yeshambel T Nigatu, Nasrin Nikravangolsefid, Vikram Niranjan, Chukwudi A Nnaji, Syed Toukir Ahmed Noor, Nawsherwan Not applicable, Jean Jacques Noubiap, Chisom Adaobi Nri-Ezedi, Fred Nugen, Jerry John Nutor, Chimezie Igwegbe Nzoputam, Ogochukwu Janet Nzoputam, Kehinde O Obamiro, Ismail A Odetokun, Onome Bright Oghenetega, Ayodipupo Sikiru Oguntade, Sylvester Reuben Okeke, Akinkunmi Paul Okekunle, Osaretin Christabel Okonji, Andrew T Olagunju, Babayemi Oluwaseun Olakunde, Oladotun Victor Olalusi, Matthew Idowu Olatubi, Abdulhakeem Abayomi Olorukooba, Isaac Iyinoluwa Olufadewa, Ahmed Omar Bali, Obinna E Onwujekwe, Abdulahi Opejin, Michal Ordak, Verner N Orish, Edgar Ortiz-Brizuela, Uchechukwu Levi Osuagwu, Amel Ouyahia, Mahesh Padukudru P A, Jagadish Rao Padubidri, Claudia Palladino, Ashok Pandey, Leonidas D Panos, Jose L Paredes, Pragyan Paramita Parija, Romil R Parikh, Ava Pashaei, Maja Pasovic, Sangram Kishor Patel, Aslam Ramjan Pathan, Shankargouda Patil, Shrikant Pawar, Veincent Christian Filipino Pepito, Emmanuel K Peprah, Prince Peprah, Marcos Pereira, Simone Perna, Ionela-Roxana Petcu, Hoang Tran Pham, Julian David Pillay, Ramesh Poluru, Maarten J Postma, Naeimeh Pourtaheri, Jalandhar Pradhan, Prem Prakash, Thejeswar N N Prakasham, Elton Junio Sady Prates, Dimas Ria Angga Pribadi, Tina Priscilla, Jagadeesh Puvvula, Ibrahim Qattea, Asma Saleem Qazi, Raghu Anekal Radhakrishnan, Quinn Rafferty, Ibrar Rafique, Fakher Rahim, Afarin Rahimi-Movaghar, Vafa Rahimi-Movaghar, Mosiur Rahman, Amir Masoud Rahmani, Shayan Rahmani, Nazanin Rahmanian, Mohammad Rahmanian, Vahid Rahmanian, Sathish Rajaa, Mahmoud Mohammed Ramadan, Hazem Ramadan, Shakthi Kumaran Ramasamy, Pushkal Sinduvadi Ramesh, Kritika Rana, Chhabi Lal Ranabhat, Mithun Rao, Sowmya J Rao, Mohammad-Mahdi Rashidi, Devarajan Rathish, Santosh Kumar Rauniyar, Salman Rawaf, Elrashdy Moustafa Mohamed Redwan, Robert C Reiner Jr., Mohsen Rezaeian, Jefferson Antonio Buendia Rodriguez, Kevin T Root, Allen Guy Ross, Kunle Rotimi, Nitai Roy, Godfrey M Rwegerera, Cameron John Sabet, Basema Ahmad Saddik, Mohammad Reza Saeb, Umar Saeed, Pooya Saeedi, Sher Zaman Zaman Safi, Rajesh Sagar, Fatemeh Saheb Sharif-Askari, Narjes Saheb Sharif-Askari, Amirhossein Sahebkar, Soumya Swaroop Sahoo, Zahra Saif, Mirza Rizwan Sajid, Nasir Salam, Afeez Abolarinwa Salami, Mohamed A Saleh, Leili Salehi, Hossein Samadi Kafil, Abdallah M Samy, Rama Krishna Sanjeev, Milena M Santric-Milicevic, Aswini Saravanan, Benn Sartorius, Anudeep Sathyanarayan, Maheswar Satpathy, Monika Sawhney, Mansour Sedighi, Birhan Ewunu Semagn, Sabyasachi Senapati, Yashendra Sethi, Allen Seylani, Pritik A Shah, Samiah Shahid, Masood Ali Shaikh, Ali Shamekh, Mohammad Ali Shamshirgaran, Anas Shamsi, Mohd Shanawaz, Mohammed Shannawaz, Amin Sharifan, Javad Sharifi-Rad, Shamee Shastry, Rekha Raghuveer Shenoy, Premalatha K Shetty, Mahabalesh Shetty, Pavanchand H Shetty, Desalegn Shiferaw, Reza Shirkoohi, Aminu Shittu, Sunil Shrestha, Migbar Mekonnen Sibhat, Emmanuel Edwar Siddig, Mark J Siedner, Jasvinder A Singh, Paramdeep Singh, Surjit Singh, Harmanjit Singh, Robert Sinto, Anna Aleksandrovna Skryabina, Amanda E Smith, Farrukh Sobia, Anton Sokhan, Shipra Solanki, Ranjan Solanki, Reed J D Sorensen, Sahabi K Sulaiman, Lukasz Szarpak, Sree Sudha T Y, Mohammad Tabish, Santosh Kumar Tadakamadla, Yasaman Taheri Abkenar, Jabeen Taiba, Iman M Talaat, Mircea Tampa, Jacques Lukenze Tamuzi, Ker-Kan Tan, Manoj Tanwar, Elvis Enowbeyang Tarkang, Nuno Taveira, Gebrehiwot Teklay, Behailu Terefe Tesfaye, Enoch Teye-Kwadjo, Ramna Thakur, Pugazhenthan Thangaraju, Rajshree Thapa, Rekha Thapar, Friedrich Thienemann, Joe Thomas, Marcos Roberto Tovani-Palone, Thang Huu Tran, Mai Thi Ngoc Tran, Alexander C Tsai, Guesh Mebrahtom Tsegay, Munkhtuya Tumurkhuu, Arit Udoh, Irfan Ullah, Atta Ullah, Muhammad Umair, Muhammad Umar, Bhaskaran Unnikrishnan, Sanaz Vahdati, Asokan Govindaraj Vaithinathan, Shoban Babu Varthya, Tommi Juhani Vasankari, Georgios-Ioannis Verras, Jorge Hugo Villafañe, Anh Truc Vo, Theo Vos, Mandaras Tariku

Walde, Richard G Wamai, Yanzhong Wang, Muhammad Waqas, Paul Ward, Gizachew Tadesse Wassie, Robert G Weintraub, Haftom Legese Weldetinsaa, Gebre Adhanom Weldu, Ronny Westerman, Nuwan Darshana Wickramasinghe, Mesfin Agachew Woldekidan, Yen Jun Wong, Nigus Kassie Worku, Zenghong Wu, Xinsheng Wu, Sajad Yaghoubi, Gesila Endashaw Yesera, Saber Yezli, Siyan Yi, Arzu Yiğit, Dehui Yin, Yazachew Yismaw, Dong Keon Yon, Naohiro Yonemoto, Fathiah Zakham, Haijun Zhang, Jingya Zhang, Hanqing Zhao, Bin Zhu, Qingyuan Zhuang, Abzal Zhumagaliuly, Magdalena Zielińska, Liu Zihao, Yossef Teshome Zikarg, Mohammad Zoladl, Alimuddin Zumla, Samer H Zyoud, Peng Zheng, Aleksandr Y Aravkin, Jeffrey W Imai-Eaton, Mohsen Naghavi, Austin E Schumacher, Simon I Hay, Christopher J L Murray**, and Hmwe H Kyu**.

* Authors share co-first authorship
** Authors share co-senior authorship

Affiliations

Institute for Health Metrics and Evaluation (A Carter MPH, M K Walters BS, D Jahagirdar PhD, E D Brewer, A Novotney MPH, D Lasher MS, A Vongpradith BA, J He MSc, S Byrne MPH, R V Dominguez BS, S A Pease BS, H Comfort MPH, E A May MS, K Burkart PhD, X Dai PhD, Prof L Degenhardt PhD, K S Ikuta MD, M B Kassel BA, K E LeGrand MPH, Prof S S Lim PhD, A W McKowen MA, T Mestrovic PhD, A H Mokdad PhD, V Mougin BA, M Pasovic MEd, Q Rafferty BA, R C Reiner Jr. PhD, A E Smith MPA, R J D Sorensen PhD, A T Vo ScM, Prof T Vos PhD, M Zhang MS, P Zheng PhD, A Y Aravkin PhD, J W Imai-Eaton PhD, Prof M Naghavi PhD, A E Schumacher PhD, Prof S I Hay FMedSci, Prof C J L Murray DPhil, H H Kyu PhD), Division of Allergy and Infectious Diseases, Department of Medicine (K Tram MD), Department of Health Metrics Sciences (E A Mpolya PhD), Department of Health Metrics Sciences, School of Medicine (K Burkart PhD, X Dai PhD, Prof S S Lim PhD, A H Mokdad PhD, R C Reiner Jr. PhD, B Sartorius PhD, Prof T Vos PhD, P Zheng PhD, A Y Aravkin PhD, Prof M Naghavi PhD, Prof S I Hay FMedSci, Prof C J L Murray DPhil, H H Kyu PhD), Department of Global Health (R J D Sorensen PhD), Department of Applied Mathematics (A Y Aravkin PhD), University of Washington, Seattle, WA, United States of America; Department of Health and Biomedical Sciences (E A Mpolya PhD), Nelson Mandela African Institution of Science and Technology, Arusha, Tanzania; Center for Biostatistics (J Ma MS), Ohio State University, Columbus, OH, United States of America; Institute for Health Metrics and Evaluation (M Verma), University of Washington, Seattle, WA; Teachers College, Columbia University (A W McKowen), New York, NY, United States of America; Vagelos College of Physicians and Surgeons (T D Frank MPH), Columbia University Medical Center, New York, NY, United States of America; The Allen Institute, Seattle, WA, United States of America (C Lin BS); Department of Clinical Governance and Quality Improvement (Y H Abate MSc), Aleta Wondo General Hospital, Aleta Wondo, Ethiopia; Advanced Diagnostic and Interventional Radiology Research Center (H Abbastabar PhD), Non-communicable Diseases Research Center (M Bastan MD, S Rahmani MD, M Rashidi MD), School of Medicine (A Behnoush BS), Iranian Research Center for HIV/AIDS (IRCHA) (O Dadras PhD), Multiple Sclerosis Research Center (S Eskandarieh PhD), Department of Virology (A Hosseinzadeh Adli PhD), Department of Pediatric Cardiology (Prof E Malakan Rad MD), Department of Epidemiology and Biostatistics (M Mansournia PhD), Tehran Heart Center (M Najafi MD), Research Center for Advanced Technologies in Cardiovascular Medicine (M Najafi MD), Iranian National Center for Addiction Studies (Prof A Rahimi-Movaghar MD), Sina Trauma and Surgery Research Center (Prof V Rahimi-Movaghar MD), Research Center for Rational Use of Drugs (A Sharifan PharmD), Cancer Research Center (R Shirkoohi PhD), Cancer Biology Research Center (R Shirkoohi PhD), Tehran University of Medical Sciences, Tehran, Iran; Department of

Mathematics and Sciences (A Abdelkader PhD), Department of Clinical Sciences (S O Alalalmeh BPharm, O E Hegazi BPharm), Center for Medical and Bio-Allied Health Sciences Research (A Shamsi PhD, S H Zyoud PhD), Ajman University, Ajman, United Arab Emirates; Department of Medicine (P Abdi BEng), Memorial University, St. John's, NL, Canada; Department of Medicine (Prof M Abdoun PhD), University of Setif Algeria, Sétif, Algeria; Department of Health, Sétif, Algeria (Prof M Abdoun PhD); Biomedical Science Department (J M Abdul Aziz MSc), Komar University of Science and Technology, Sulaymaniyah, Iraq; Baxshin Hospital (J M Abdul Aziz MSc), Baxshin Research center, Sulaymaniyah, Iraq; Laboratory Technology Sciences Department (H Abidi PhD), Department of Nursing (M Zoladl PhD), Yasuj University of Medical Sciences, Yasuj, Iran; Department of Community Medicine (O Abiodun MPH), Babcock University, Ilishan-Remo, Nigeria; Department of Family and Community Health (R G Aboagye MPH), Department of Epidemiology and Biostatistics (L A Adzigbli BSc), Department of Population and Behavioural Sciences (H Amu PhD, Prof E E Tarkang PhD), Department of Microbiology and Immunology (Prof V N Orish PhD), University of Health and Allied Sciences, Ho, Ghana; Department of Pediatric Dentistry of the School of Dentistry (Prof L Abreu PhD), Department of Maternal-Child Nursing and Public Health (Prof A C Micheletti Gomide Nogueira de Sá MSc, E J S Prates BS), Federal University of Minas Gerais, Belo Horizonte, Brazil; Department of Biomedical science (Y D Abtew MSc), Department of Midwifery (A C Belete MSc), School of Nursing (G E Yesera MSc), Arba Minch University, Arba Minch, Ethiopia; Department of Biopharmaceutics and Clinical Pharmacy (Prof E Abu-Gharbieh PhD), Diagnostic Radiology and Nuclear Medicine (Prof A Al-Ibraheem MD), School of Pharmacy (Prof Y Bustanji PhD), The University of Jordan, Amman, Jordan; Clinical Sciences Department (Prof E Abu-Gharbieh PhD, H J Barqawi MPhil, N R Dash MD, Prof R Halwani PhD, Prof M M Ramadan PhD, N Saheb Sharif-Askari PhD, Prof I M Talaat PhD), Department of Basic Biomedical Sciences (Prof Y Bustanji PhD), College of Medicine (Prof R Halwani PhD, Prof B A Saddik PhD, Prof M A Saleh PhD), Sharjah Institute of Medical Sciences (F Saheb Sharif-Askari PhD), University of Sharjah, Sharjah, United Arab Emirates; Department of Therapeutics (Prof S Aburuz PhD), Department of Medical Microbiology & Immunology (Prof G Khan PhD), United Arab Emirates University, Al Ain, United Arab Emirates; College of Pharmacy (Prof S Aburuz PhD), University of Jordan, Amman, Jordan; Department of Biochemistry and Molecular Medicine (A Abu-Zaid PhD), College of Medicine (Prof Z A Memish MD), Alfaisal University, Riyadh, Saudi Arabia; College of Graduate Health Sciences (A Abu-Zaid PhD), University of Tennessee, Memphis, TN, United States of America; Centre for Social Research in Health (I Y Addo PhD, S R Okeke PhD), National Drug and Alcohol Research Centre (Prof L Degenhardt PhD), School of Population Health, Faculty of Medicine and Health (Prof B A Saddik PhD), University of New South Wales, Sydney, NSW, Australia; Quality and Systems Performance Unit (I Y Addo PhD), Cancer Institute NSW, Sydney, NSW, Australia; Menzies School of Health Research (Prof O A Adegboye PhD), Charles Darwin University, Darwin, NT, Australia; Department of Obstetrics and Gynecology (V Adekanmbi PhD), University of Texas Medical Branch, Galveston, TX, United States of America; Department of Microbiology (Prof C O O Adetunji PhD), Edo State University Uzairue, Iyamho, Nigeria; Department of Biochemistry (J B Adetunji PhD), Osun State University, Osogbo, Nigeria; Department of Community Health and Epidemiology (D A Adeyinka PhD), University of Saskatchewan, Saskatoon, SK, Canada; Department of Public Health (D A Adeyinka PhD), Federal Ministry of Health, Abuja, Nigeria; School of Public Health (Prof K Adhikari PhD), Tribhuvan University, Bharatpur, Nepal; Public Health Section (Prof K Adhikari PhD), Himalayan Environment and Public Health Network (HEPHN), Chitwan, Nepal; Department of Public Health (Q Adnani PhD), Padjadjaran University, Bandung, Indonesia; School of Medicine (F Afrashteh MD, M Bastan MD), Minimally Invasive Surgery Research Center (A Kabir MD), Endocrine Research Center (A Karimi Behnagh

MD), Department of Echocardiography (A Karimi Behnagh MD), Research Center of Pediatric Infectious Diseases (F Khamesipour PhD), Department of Health Economics (M Khosravi PhD), Iran University of Medical Sciences, Tehran, Iran (M Moradi MD); Department of Community Medicine (Prof S Afzal PhD), King Edward Memorial Hospital, Lahore, Pakistan; Department of Public Health (Prof S Afzal PhD), Public Health Institute, Lahore, Pakistan; Department of Biotechnology (S Aghamiri PhD), Department of Internal Medicine (H Borhany MD), Obesity Research Center (A Haj-Mirzaian MD), Ophthalmic Epidemiology Research Center (Z Khorrami PhD), School of Medicine (S Rahmani MD), Student Research Committee (M Rahmanian MD), Social Determinants of Health Research Center (M Rashidi MD), Shahid Beheshti University of Medical Sciences, Tehran, Iran; Department of Health Education and Health Promotion (F D Agide PhD), Department of Public Health (M Y Ashemo MPH, D Handiso MPH), Wachemo University, Hossana, Ethiopia; Department of Medical and Surgical Sciences and Advanced Technologies "GF Ingrassia" (Prof A Agodi PhD), University of Catania, Catania, Italy; Department of Geography and Planning (W Agyemang-Duah PhD), Queen's University, Kingston, ON, Canada; School of Public Health (B O Ahinkorah MPhil), University of Technology Sydney, Sydney, NSW, Australia; Biological Production Unit National Institute of Health Islamabad Pakistan (F Ahmad), National Institute of Health, Islamabad, Pakistan; World Health Organization - Pakistan (F Ahmad), World Health Organisation, Islamabad, Pakistan; Department of Health and Biological Sciences (S Ahmad PhD), Abasyn University, Peshawar, Pakistan; Department of Natural Sciences (S Ahmad PhD), Lebanese American University, Beirut, Lebanon; Department of Medical Oncology (S Ahmad MD), Department of Medicine (M Ganiyani MD), Miami Cancer Institute, Miami, FL, United States of America; Department of Community Medicine and Preventive Health (S Ahmad MD), King Edward Medical University Lahore, Lahore, Pakistan; Department of Medical Biochemistry (A Ahmad PhD), College of Medicine (Prof G Mustafa MD), Department of Pharmacology (A R Pathan PhD, M Tabish MPharm), Shaqra University, Shaqra, Saudi Arabia; R&D (I Ahmed PhD), Alpha Genomics Private Limited, Islamabad, Pakistan (Z Basharat PhD); Microbiological Analysis Team (I Ahmed PhD), Korea Research Institute of Standards and Science (KRISS), Daejeon, South Korea; Department of Biosciences (H Ahmed PhD), COMSATS Institute of Information Technology, Islamabad, Pakistan; Brody School of Medicine (S Ahmed PhD), Department of Computer Science (A O Bodunrin MSc), Department of Geography (A Opejin MSc), Department of Physiology (M Tumurkhuu PhD), East Carolina University, Greenville, NC, United States of America; Department of Biochemistry (S Ahmed BSc), Jamia Hamdard, Delhi, India; Department of Pharmacy Practice (A Ahmed PhD), Riphah Institute of Pharmaceutical Sciences, Islamabad, Pakistan; Division of Infectious Diseases and Global Public Health (IDGPH) (A Ahmed PhD), University of California San Diego, San Diego, CA, United States of America; Health Systems Strengthening (M Ahmed MPH), State Health Resource Centre, Raipur, India; Institute of Endemic Diseases (A Ahmed MSc), Unit of Basic Medical Sciences (E E Siddig MD), University of Khartoum, Khartoum, Sudan; Swiss Tropical and Public Health Institute (A Ahmed MSc), University of Basel, Basel, Switzerland; Department of Microbiology, Immunology and Parasitology (G T Akalu MSc), St. Paul's Hospital Millennium Medical College, Addis Ababa, Ethiopia; Department of Microbial, Cellular, and Molecular Biology (G T Akalu MSc), School of Public Health (K Deribe PhD), Department of Anatomy (Y T Zikarg MSc), Addis Ababa University, Addis Ababa, Ethiopia; Department of Internal Medicine (K Akinosoglou PhD), University of Patras, Patras, Greece; Department of Internal Medicine and Infectious Diseases (K Akinosoglou PhD), University General Hospital of Patras, Patras, Greece; Department of Communicable Diseases (S Al Awaidy MSc), Ministry of Health, Muscat, Oman; Middle East, Eurasia, and Africa Influenza Stakeholders Network, Muscat, Oman (S Al Awaidy MSc); Department of Geriatric and Long Term Care (H Al Hamad MD),

Rumailah Hospital (H Al Hamad MD), Hamad Medical Corporation, Doha, Qatar; Department of Medicine (A S Al Mosa MD), Division of Forensic Medicine (Prof R G Menezes MD), Imam Abdulrahman Bin Faisal University, Dammam, Saudi Arabia (F M Alanezi PhD); Department of Adult Health and Critical Care, College of Nursing (O A M Al Zaabi PhD), Department of Geography (W Ali PhD), Adult Health and Critical Care (D Altwalbeh PhD), Sultan Qaboos University, Muscat, Oman; Department of Public Health (Prof N Alam DrPH), Asian University for Women, Chittagong, Bangladesh; Asian University for Women (Prof N Alam DrPH), McGill University, Chittagong, Bangladesh; Public Health Intelligence Branch (N Alam MAE), Department of Medicine (V Kulkarni MS), Queensland Health, Brisbane, QLD, Australia; Centre for Environment and Population Health (N Alam MAE), Griffith University, Nathan, QLD, Australia; Department of Epidemiology and Biostastics (D S Alayu MPH), Department of Pharmaceutics (L W Limenh MSc), Department of Internal Medicine (E Melese MD), University of Gondar, Gondar, Ethiopia; Faculty of Medicine (Prof M T AlBataineh PhD), Yarmouk University, Irbid, Jordan; Robert Stempel College of Public Health and Social Work (S Alemohammad MD), Florida International University, Miami, FL, United States of America; Global Centre for Environmental Remediation (A A S Al-Gheethi PhD), University of Newcastle, Newcastle, NSW, Australia; Cooperative Research Centre for Contamination Assessment and Remediation of the Environment, Newcastle, NSW, Australia (A A S Al-Gheethi PhD); Center for Biotechnology and Microbiology (S S Ali PhD), University of Swat, Swat, Pakistan; Department of Medical Rehabilitation (Physiotherapy) (M U Ali MSc), Department of Microbiology (M A Isa PhD), University of Maiduguri, Maiduguri, Nigeria; Department of Rehabilitation Sciences (M U Ali MSc, M Khan MPH), Department of Applied Social Sciences (C T Antonio MD), Hong Kong Polytechnic University, Hong Kong, China; Department of Zoology (A Ali PhD), Abdul Wali Khan University Mardan, Mardan, Pakistan; Department of Biological Sciences (L Ali PhD, A S Qazi PhD), National University of Medical Sciences (NUMS), Rawalpindi, Pakistan; Department of Nuclear Medicine (Prof A Al-Ibraheem MD), King Hussein Cancer Center, Amman, Jordan; Department of Medicine (J U Almazan PhD, A Gaipov PhD), Nazarbayev University, Astana, Kazakhstan; Institute of Molecular Biology and Biotechnology (A Altaf PhD, S Shahid PhD), University Institute of Radiological Sciences and Medical Imaging Technology (T Ashraf MS), Department of Oral Biology (A Fahim PhD), University Institute of Diet and Nutritional Sciences (A Khalil PhD), University Institute of Public Health (S Nargus PhD), Research Centre for Health Sciences (RCHS) (S Shahid PhD), Lahore Business School (M Umar MBA), The University of Lahore, Lahore, Pakistan; Department of Allied Medical science (D Altwalbeh PhD), AL-Balqa Applied University, karak, Jordan; Research Group in Health Economics (Prof N Alvis-Guzman PhD), Universidad de Cartagena, Cartagena, Colombia; Research Group in Hospital Management and Health Policies (Prof N Alvis-Guzman PhD), Universidad de la Costa, Barranquilla, Colombia; Department of Biomedical Engineering (W A Al-Zyoud PhD), German Jordanian University, Amman, Jordan; Interdisciplinary Graduate Program in Human Toxicology (R Amani DVM), University of Iowa, Iowa City, IA, United States of America; Health Policy Research Center (R Amani DVM, A Ardekani MD), Shiraz University of Medical Sciences, Shiraz, Iran; Department of Public Health (T G Amera MPH, H Mohammed PhD, N K Worku MPH), Dire Dawa University, Dire Dawa, Ethiopia; School of Graduate Studies (E K Ameyaw MPhil), Lingnan University, Hong Kong, China; Quran and Hadith Research Center (S Amiri PhD), Nephrology and Urology Research Center (K Hushmandi PhD), Baqiyatallah University of Medical Sciences, Tehran, Iran; Department of Medicine (G A Amusa MD), University of Jos, Jos, Nigeria; Department of Internal Medicine (G A Amusa MD), Jos University Teaching Hospital, Jos, Nigeria; Department of Pharmacology (A Anil MD, S Singh MD, S B Varthya MD), Department of Community Medicine and Family Medicine (Prof P Bhardwaj MD), School of Public Health (Prof P Bhardwaj MD),

Department of Anatomy (N Bhardwaj MD), Department of Anesthesiology and Critical Care (R Kumar MD), Department of Pharmacology and Research (A Saravanan MD), All India Institute of Medical Sciences, Jodhpur, India; All India Institute of Medical Sciences, Bhubaneswar, India (A Anil MD); Department of Microbiology (A A Anjorin PhD), Lagos State University, Ojo, Lagos, Nigeria; Department of Health Policy and Administration (C T Antonio MD), Department of Epidemiology and Biostatistics (Prof A C Bermudez MD), National Institutes of Health (A Loreche BS), University of the Philippines Manila, Manila, Philippines; Centre for Interdisciplinary Research in Basic Sciences (CIRBSc) (S Anwar PhD), Department of Biosciences (N Salam PhD), Centre For Interdisciplinary Research In Basic Sciences (CIRBSc) (A Shamsi PhD), Jamia Millia Islamia, New Delhi, India; School of Chemical and Life Sciences (SCLS) (S Anwar PhD), Jamia Hamdard, New Delhi, India; Department of Pathology (R Anwer PhD), Imam Mohammad Ibn Saud Islamic University, Riyadh, Saudi Arabia; Department of Medical Laboratory Sciences (E E Anyabolo BMLSc, M O Asika BMLSc), Department of Pharmacology and Therapeutics (Prof O E Onwujekwe PhD), University of Nigeria Nsukka, Enugu, Nigeria; Operations Department (E E Anyabolo BMLSc), Breast Without Spot, Enugu, Nigeria; Rural Health Research Institute (A E Anyasodor PhD), Rural Health research Institute (Prof A G Ross MD), Charles Sturt University, Orange, NSW, Australia; School of Medicine and Public Health (G C Apostol MD, A Loreche BS), Center for Research and Innovation, School of Medicine and Public Health (V F Pepito MSc), Ateneo De Manila University, Pasig City, Philippines; Inter-Agency Committee on Environmental Health (G C Apostol MD), Department of Health Philippines, Manila, Philippines; College of Art and Science (e Areda PhD), Ottawa University, Surprise, AZ, United States of America; School of Life Sciences (e Areda PhD), Arizona State University, Tempe, AZ, United States of America; College of Medicine and Health Sciences (B B Aregawi PhD), Department of Midwifery (M W Gebregergis MSc), Department of Medical Laboratory Sciences (H N Meles MSc, H L Weldetinsaa MSc, G A Weldu MSc), Adigrat University, Adigrat, Ethiopia; Department of Veterinary Pharmacology and Toxicology (A Aremu PhD), Department of Veterinary Physiology and Biochemistry (A Basiru PhD), Department of Veterinary Public Health and Preventive Medicine (I A Odetokun PhD), University of Ilorin, Ilorin, Nigeria; School of Public Health (K Armani PhD), Department of Infection (C S Brown MD), Department of Primary Care and Public Health (Prof A Majeed MD, Prof S Rawaf MD), Department of Infectious Disease Epidemiology (J W Imai-Eaton PhD), Imperial College London, London, United Kingdom; Faculty of Pharmaceutical Sciences (K Armani PhD), UCSI University, Kuala Lumpur, Malaysia; School of Public Health (M A Asemahagn PhD), Department of Midwifery (W F Balcha MSc), School of Health Science (A Y Berhie MSc), Reproductive Health (G W Dagnew MPH), Department of Pediatrics and Child Health (B S Firew MD), Department of Medical Microbiolog and Virology (A D Habteyohannes PhD), Department of Pharmacy (C T Negesse MSc), Department of Epidemiology and Biostatistics (G T Wassie MPH), Department of Pharmacology (Y Yismaw MSc), Bahir Dar University, Bahir Dar, Ethiopia; Department of Public Health (M Y Ashemo MPH, M E Getachew MPH), Department of Surgery (N S Bayleyegn MD), Department of Epidemiology (D Shiferaw MPH), Department of Clinical Pharmacy (B Tesfaye MSc), Jimma University, Jimma, Ethiopia; Department of Telemedicine (M O Asika BMLSc), Society For Disease Prevention, Inc., Hummelstown, PA, United States of America; School of Medical Laboratory Sciences (H A Asmerom MSc, F Ayele MSc), Department of Clinical Pharmacy (M D Gudeta MSc), Department of Psychiatry (M T Walde MSc), Haramaya University, Harar, Ethiopia; Faculty of Nursing (M M W Atout PhD), Philadelphia University, Amman, Jordan; Northumbria HealthCare NHS Foundation Trust, Newcastle upon Tyne, United Kingdom (A Aujayeb MBBS); Department of Health Sciences (H Awad PhD), Higher College of Technology, Abu Dhabi, United Arab Emirates; Department of Physiotherapy (A W Awotidebe PhD), Department of Community

Medicine (Prof M A Gadanya MD), Department of Nursing Science (M Ladan PhD), Bayero University Kano, Kano, Nigeria; School of Nursing and Public Health (A W Awotidebe PhD, Prof E E Tarkang PhD), School of Nursing and Public Health Medicine (M M Hlongwa PhD), University of KwaZulu-Natal, Durban, South Africa; The Judith Lumley Centre (B Ayala Quintanilla PhD), School of Nursing and Midwifery (F Efendi PhD), La Trobe University, Melbourne, VIC, Australia; Universidad de San Martin de Porres, Lima, Peru (B Ayala Quintanilla PhD); Leeds Institute of Rheumatic and Musculoskeletal Medicine (S Azadnajafabad MD), University of Leeds, Leeds, United Kingdom; Institute of Biotechnology and Genetic Engineering (S Aziz MS), The University of Agriculture, Peshawar, Pakistan; Kasturba Medical College, Mangalore (D B B MD), Prasanna School of Public Heath (PSPH) (H L Dsouza MD, V S Dsouza MSc), Department of Pharmaceutical Regulatory Affairs and Management (V S Ligade PhD), Department of Forensic Medicine, Kasturba Medical College, Manipal (Prof V C Nayak MD), Manipal College of Dental Sciences (Prof R A Radhakrishnan PhD), Kasturba Medical College Mangalore (M Rao MD, Prof B Unnikrishnan MD), Department of Immunohematology and Blood Transfusion (Prof S Shastry MD), Department of Pharmacology (R R Shenoy PhD), Manipal Academy of Higher Education, Manipal, India; Department of Population Medicine (Prof G Babu PhD), QU Health (M Mohammed PhD), Qatar University, Doha, Qatar; Gomal Center of Biochemistry and Biotechnology (M Badar PhD), Gomal University, Dera Ismail Khan, Pakistan; School of Medicine (S Bahramian MD), Department of Environmental Health Engineering (A Fatehizadeh PhD), Isfahan University of Medical Sciences, Isfahan, Iran; Department of Neurosurgery (A T Bako PhD), Houston Methodist Hospital, Houston, TX, United States of America (M Elhadi MD); Department of Medicine (K Bam MPH), Monash University, Clayton, VIC, Australia; Institute of Health and Wellbeing (B Banik PhD), Federation University Australia, Melbourne, VIC, Australia; Manna Institute (B Banik PhD), University of New England, Armidale, NSW, Australia; Miami Cancer Institute (M Bardhan MD), Baptist Health South Florida, Miami, FL, United States of America; Heidelberg Institute of Global Health (HIGH) (Prof T W Bärnighausen MD, B Moazen MSc), Heidelberg University, Heidelberg, Germany; T.H. Chan School of Public Health (Prof T W Bärnighausen MD), Department of Global Health and Population (A Iradukunda MD), Harvard Medical School (M J Siedner MD), Harvard University, Boston, MA, United States of America; Department of Animal Sciences (H A Bashiru PhD), Department of Child Dental Health (Prof M O Folayan FWACS), Obafemi Awolowo University, Ile-Ife, Nigeria; Department of Academics (S Basu MD), Indian Institute of Public Health, Gurgaon, India; Department of Clinical Pharmacology (Prof P P Bathini MD), Department of Dermatology, Venereology and Leprosy-DVL (Prof T Priscilla MD), Apollo Institute of Medical Sciences and Research, Hyderabad, India; Department of Medical Education (K Batra PhD), School of Public Health (R Batra MS), University of Nevada Las Vegas, Las Vegas, NV, United States of America; IT Department (R Batra MS), Coforge, Georgia, GA, United States of America; Center of Research Excellence in Stillbirth (T Begum PhD), Department of Urology (Prof E Chung MD), Faculty of Medicine (B Sartorius PhD), The University of Queensland, Brisbane, QLD, Australia (M Moni PhD); Health System and Population Studies Division (T Begum PhD), Department of Maternal and Child health (S Noor MS), International Centre for Diarrhoeal Disease Research, Bangladesh, Dhaka, Bangladesh; Non-Communicable Diseases Research Center (NCDRC), Tehran, Iran (A Behnoush BS, F Montazeri MD); Division of Pulmonary, Critical Care, and Sleep (M Beiranvand PhD), College of Medicine (A A Beneke MS, K T Root BS), Department of Epidemiology (D D Ding BS), University of Florida, Gainesville, FL, United States of America; Department of Medical Laboratory Science (M A Belete MSc), Department of Environmental Health (M Gebrehiwot DSc), Wollo University, Dessie, Ethiopia; Department of Biomedical Sciences (Prof A Beloukas PhD), National AIDS Reference Center of Southern Greece (Prof A

Beloukas PhD), University of West Attica, Athens, Greece; School of Medicine (A Beran MD), Indiana University, Indianapolis, IN, United States of America; Department of Epidemiology (Prof A C Bermudez MD), Department of Biology and Medicine (P Fazeli MSc), The Warren Alpert Medical School (Z A Haq BA), Brown University, Providence, RI, United States of America; Hubert Department of Global Health (R S Bernstein MD), Emory University, Atlanta, GA, United States of America; Department of Global Health (R S Bernstein MD), George Washington University, Washington, DC, United States of America; Department of Pharmaceutical and Administrative Sciences (K A Beyene PhD), University of Health Sciences and Pharmacy in St. Louis, St Louis, MO, United States of America; School of Pharmacy (K A Beyene PhD), University of Auckland, Auckland, New Zealand; Department of General Medicine (A N Bhat MD), Department of Community Medicine (N Joseph MD, N Kumar MD, R Motappa MD, R Thapar MD), Department of Anatomy (B Murlimanju MD), Department of Forensic Medicine and Toxicology (Prof J Padubidri MD, P H Shetty MD), Manipal College of Dental Sciences (Prof P K Shetty MDS), Manipal Academy of Higher Education, Mangalore, India; Department of Internal Medicine (V Bhat MBBS), St. John's National Academy of Health Sciences, Bangalore, India; Department of Medical Lab Technology (Prof G K Bhatti PhD), Chandigarh University, Mohali, India; Department of Human Genetics and Molecular Medicine (Prof J S Bhatti PhD, S Senapati PhD), Central University of Punjab, Bathinda, India; Department of Midwifery (K A Bishaw MSc), Debre Markos University, Debre Markos, Ethiopia; Department of Pharmacology (K D Bisht DM), Amrita Institute of Medical Sciences, Faridabad, India; Pharmacology (K D Bisht DM), Department of Paediatrics (Prof R Lodha MD), Department of Psychiatry (Prof R Sagar MD), All India Institute of Medical Sciences, New Delhi, India; Department of Community & Family Medicine (Prof T Bodhare MD), All India Institute of Medical Sciences, Ramanathapuram, Tamil Nadu, India; Department of Mental Health/Bikhara state medical institute (A A Boltaev MD), Tashkent Institute of Postgraduate Medical Education, Bukhara, Uzbekistan; Department of Medicine (Prof S Bouaoud DrPH), Faculty of Medicine (Prof A Ouyahia PhD), University Ferhat Abbas of Setif, Setif, Algeria; Department of Epidemiology and Preventive Medicine (Prof S Bouaoud DrPH), University Hospital Saadna Abdenour, Setif, Algeria; HCAI, Fungal, AMR, AMU, & Sepsis Division (C S Brown MD), United Kingdom Health Security Agency, London, United Kingdom; Department of Woman and Child Health and Public Health (D Buonsenso MD), Fondazione Policlinico Universitario A. Gemelli IRCCS (Agostino Gemelli University Polyclinic IRCCS), Rome, Italy; Global Health Research Institute (D Buonsenso MD), Università Cattolica del Sacro Cuore (Catholic University of Sacred Heart), Rome, Italy; School of Public Health Sciences (Z A Butt PhD), University of Waterloo, Waterloo, ON, Canada; Al Shifa School of Public Health (Z A Butt PhD), Al Shifa Trust Eye Hospital, Rawalpindi, Pakistan; Dana-Farber Cancer Institute, Boston, MA, United States of America (C Cao MPH); Department of Health Care (Prof R Cárdenas DSc), Metropolitan Autonomous University, Mexico City, Mexico; College of Public Health, Medical, and Veterinary Sciences (M Cenderadewi MPHTM), James Cook University, Townsville, QLD, Australia (K O Obamiro PhD); Department of Public Health (M Cenderadewi MPHTM), University of Mataram, Mataram, Indonesia; Noncommunicable Diseases Division (J Chadwick MD), National Institute of Epidemiology, Chennai, India; Department of Biotechnology (Prof C Chakraborty PhD), Adamas University, Kolkata, India; Institute for Skeletal Aging & Orthopedic Surgery (Prof C Chakraborty PhD), Hallym University, Chuncheon, South Korea; State Disease Investigation Laboratory (S Chakraborty MVSc), Animal Resources Development Department, Agartala, India; Department of Clinical Nutrition (R M Chandika PhD), Department of Epidemiology (S Dohare MD), Department of Health Education and Promotion (M Shanawaz MD, F Sobia PhD), Jazan University, Jazan, Saudi Arabia; Temerty Faculty of Medicine (V Chattu MD), University of Toronto, Toronto, ON, Canada; Department of Community

Medicine (V Chattu MD), Datta Meghe Institute of Medical Sciences, Sawangi, India; Department of Oral Medicine and Radiology (A Chaurasia MD), Department of Neurology (Prof H S Malhotra DM), King George's Medical University, Lucknow, India; Department of Stomatology (G Chen DMD), Division of Gastroenterology (Prof Z Wu PhD), Huazhong University of Science and Technology, Wuhan, China; Hubei Province Key Laboratory of Oral and Maxillofacial Development and Regeneration, Wuhan, China (G Chen DMD); Division of Infectious Diseases (P R Ching MD), Virginia Commonwealth University, Richmond, VA, United States of America; Centre for Research Impact & Outcome (H Chopra PhD), Chitkara University, Rajpura, India; Department of Community Medicine (Prof S G Choudhari MD), Jawaharlal Nehru Medical College, Wardha, India; Center for Biomedicine and Community Health (D Chu PhD), International School, Vietnam National University Hanoi (VNUIS), Hanoi, Vietnam; Department of Paediatric Surgery (I S Chukwu BMedSc), Federal Medical Centre, Umuahia, Nigeria; Department of AndroUrology (Prof E Chung MD), AndroUrology Centre, Brisbane, QLD, Australia; Department of Genetics (Z Cindi PhD), School of Veterinary Medicine (F Musaigwa PhD), Department of Biostatistics, Epidemiology, and Informatics (J Puvvula PhD), Department of Otorhinolaryngology (P S Ramesh PhD), University of Pennsylvania, Philadelphia, PA, United States of America; Department of Chemical Sciences (R A S Couto MD), Institute for Research and Innovation in Health (i3S) (Prof N Cruz-Martins PhD), Institute for Research and Innovation in Health (J das Neves PhD), Institute of Biomedical Engineering (INEB) (J das Neves PhD), University of Porto, Porto, Portugal; Department of Diagnostic and Therapeutic Technologies (Prof N Cruz-Martins PhD), Cooperativa de Ensino Superior Politécnico e Universitário (Polytechnic and University Higher Education Cooperative), Vila Nova de Famalicão, Portugal; Center for Health Systems Research (S M Cuadra-Hernández PhD), National Institute of Public Health, Cuernavaca, Mexico; Epidemiology & Biostatistics, College of Public Health (B Dabo MSPH), Department of Medical Engineering (D H Nguyen BS), University of South Florida, Tampa, FL, United States of America; Department of Medical Microbiology and Parasitology (B Dabo MSPH), Bayero University, Kano, Nigeria; Department of Global Public Health and Primary Care (O Dadras PhD), Center for International Health (CIH) (S Hassan MPhil), Bergen Center for Ethics and Priority Setting (BCEPS) (S Hassan MPhil), University of Bergen, Bergen, Norway; Department of Community Medicine (Prof T Dahiru MA, A A Olorukooba MD), Ahmadu Bello University, Zaria, Nigeria; Department of Information Technology (A M Darwesh PhD), Department of Computer Science (Prof M Hosseinzadeh PhD), Diplomacy and Public Relations Department (A Omar Bali PhD), University of Human Development, Sulaymaniyah, Iraq; Immunology Research Center (M Dashti MD, A Ghasemzadeh MD), Department of Virology (A Hosseinzadeh Adli PhD), Social Determinants of Health Research Center (Prof S Mohammad-Alizadeh-Charandabi PhD), Midwifery Department (Prof S Mohammad-Alizadeh-Charandabi PhD), Drug Applied Research Center (H Samadi Kafil PhD), Faculty of Medicine (A Shamekh MD), Aging Research Institute (A Shamekh MD), Tabriz University of Medical Sciences, Tabriz, Iran; Department of Public Health (Prof F P De la Hoz PhD), National University of Colombia, Bogota, Colombia; Albany Medical College, Albany, NY, United States of America (S Debopadhaya BS); School of Medicine (I Delgado-Enciso DSc), University of Colima, Colima, Mexico; Department of Research (I Delgado-Enciso DSc), Colima State Cancer Institute, IMSS-Bienestar, Colima, Mexico; Wellcome Trust Brighton and Sussex Centre for Global Health Research (K Deribe PhD), Brighton and Sussex Medical School, Brighton, United Kingdom; Department of Psychiatry (Prof D C Des Jarlais PhD), Icahn School of Medicine at Mount Sinai, New York, NY, United States of America; Research Department (H D Desai MD), Gujarat Adani Institute of Medical Sciences and G.K. General Hospital, Bhuj, India; National Centre for AIDS and STD Control (K Deuba DrPH), Save the Children, Kathmandu, Nepal; Department of Global Public Health (K Deuba DrPH), Karolinska Institute, Stockholm, Sweden; Research

and Development Cell (A S Dhane MBA), Dr. D. Y. Patil University, Pune, India; Department of Pharmacy Practice (S Dhingra PhD), National Institute of Pharmaceutical Education and Research Hajipur, Hajipur, India; Faculty of Science (Prof D Diaz PhD), National Autonomous University of Mexico, Mexico City, Mexico; Children Without Worms (M R Diaz MPH), Task Force for Global Health, Decatur, GA, United States of America; Department of Medicine (T C Do MD), Pham Ngoc Thach University of Medicine, Ho Chi Minh City, Vietnam; Health Science Center (D Dongarwar MS), University of Texas, Houston, TX, United States of America; Departamento de Responsabilidade Social (Department of Social Responsibility) (W M dos Santos PhD), Oswaldo Cruz German Hospital, São Paulo, Brazil; Brazilian Centre for Evidence-based Healthcare (W M dos Santos PhD), Joanna Briggs Institute, São Paulo, Brazil; Independent Consultant, South Plainfield, NJ, United States of America (O P Doshi MSc); Department of Medicine (A C Dsouza MBBS, A Sathyanarayan MD), Bangalore Medical College and Research Institute, Bangalore, India; Department of Forensic Medicine and Toxicology (H L Dsouza MD), Kasturba Medical College Mangalore, Mangalore, India; Faculty of Science and Humanities (S Duraisamy PhD), Sri Ramaswamy Memorial Institute of Science and Technology, Kattankulathur, India; Department of Conservative Dentistry with Endodontics (A M Dziedzic DSc), Medical University of Silesia, Katowice, Poland; Department of Orthopaedic Surgery (A Ebrahimi MD), Department of Radiology (A Haj-Mirzaian MD), Division of Cardiology (D H Nguyen BS), Department of Psychiatry (A C Tsai MD), Massachusetts General Hospital, Boston, MA, United States of America (M Kim MD); Higher School of Technology (Prof A Ed-Dra PhD), Sultan Moulay Slimane University, Beni Mellal, Morocco; School of Health Sciences (H A Edinur PhD), Universiti Sains Malaysia, Kubang Kerian, Malaysia; Department of Community Health Nursing (F Efendi PhD), Department of Epidemiology, Biostatistics, Population Studies and Health Promotion (A Hargono MD), Universitas Airlangga (Airlangga University), Surabaya, Indonesia; Faculty of Science and Health (M Ekholuenetale PhD), University of Portsmouth, Hampshire, United Kingdom; Department of Microbiology (T C Ekundayo PhD), University of Medical Sciences, Ondo, Ondo, Nigeria; Biomedical Informatics and Medical Statistics Department (I El Sayed PhD), Department of Pathology (Prof I M Talaat PhD), Alexandria University, Alexandria, Egypt; Faculty of Medicine (M Elhadi MD), University of Tripoli, Tripoli, Libya; Department of Pediatrics (C Eltaha MD), Texas A&M University, Dallas, TX, United States of America; Department of Bacteriology and Virology (M Eslami PhD), Department of Immunology (D Haghmorad PhD), Cancer Research Center (D Haghmorad PhD), Semnan University of Medical Sciences, Semnan, Iran; Cancer Research Center (M Eslami PhD), Semnan University of Medical Scinces, Semnan, Iran; Department of Ophthalmology (U A Eze MD), Federal Medical Centre, Asaba, Nigeria; Postgraduate School (U A Eze MD), College of Medicine and Veterinary Medicine (G Verras MD), University of Edinburgh, Edinburgh, United Kingdom; Centre for Public Health, Equity and Human Flourishing (N K Fauk PhD), Centre for Health Policy Research (Prof P Ward PhD), Torrens University Australia, Adelaide, SA, Australia; Institute of Resource Governance and Social Change, Kupang, Indonesia (N K Fauk PhD); Department of Public Health and Infectious Diseases (G Fekadu PhD), City University of Hong Kong, Hong Kong, China; Department of Pharmacy (G Fekadu PhD), Department of Public Health (M E Getachew MPH), Wollega University, Nekemte, Ethiopia; Department of Social Sciences (Prof N Ferreira PhD), University of Nicosia, Nicosia, Cyprus; Institute of Public Health (F Fischer PhD), Charité Medical University Berlin, Berlin, Germany; Clinical Science Department (Prof M O Folayan FWACS), Nigerian Institute of Medical Research, Lagos, Nigeria; Department of Pharmacology (Prof B Foroutan PhD), Iranshahr University of Medical Sciences, Iranshahr, Iran; Department of Dermatology (T Fukumoto PhD), Kobe University, Kobe, Japan; Department of Community & Family Medicine (S G MD, V J MD), All India Institute of Medical Sciences, Gorakhpur, India; Department of

Community Medicine (Prof M A Gadanya MD), Aminu Kano Teaching Hospital, Kano, Nigeria; Department of Community Medicine (Prof A M Gaidhane MD), Datta Meghe Institute of Medical Sciences, Wardha, India; Department of Community & Family Medicine (A P Gandhi MD), All India Institute of Medical Sciences, Nagpur, India; Department of General Medicine (M Ganiyani MD), Grant Medical College & Sir J.J. Group of Hospitals, Mumbai, India; Department of Reproductive and Family Health (T G Gebremeskel PhD), Axum College of Health Science, Axum, Ethiopia; College of Medicine and Public Health (T G Gebremeskel PhD), Flinders University, Adelaide, SA, Australia; Infectious Disease Research Center (Prof K Ghadiri MD), Pediatric Department (Prof K Ghadiri MD), Kermanshah University of Medical Sciences, Kermanshah, Iran; School of Public Health (A Ghashghaee BSc), Qazvin University of Medical Sciences, Qazvin, Iran; Iran University of Medical Sciences, (A Ghashghaee BSc); Electrical and Computer Engineering (E Gholami PhD), University of California Davis, Davis, CA, United States of America; Department of Dermatology (N Gholizadeh MD), Mazandaran University of Medical Sciences, Sari, Iran; Orthodontics Department (M Ghorbani DDS), Endodontics Department (P Saeedi DDS), Applied Biomedical Research Center (Prof A Sahebkar PhD), Biotechnology Research Center (Prof A Sahebkar PhD), Mashhad University of Medical Sciences, Mashhad, Iran; Country Office (A U Gil PhD), World Health Organization (WHO), Astana, Kazakhstan; Moscow Medical Academy, (A U Gil PhD); Department of Nursing (A A Girmay MSc, G M Tsegay MSc), Aksum University, Aksum, Ethiopia; Department of Health Systems and Policy Research (M Golechha PhD), Indian Institute of Public Health, Gandhinagar, India; Department of Life Sciences, Health and Healthcare Professions (Prof D Golinelli MD), Link Campus University, Rome, Italy; Health Services Research, Evaluation and Policy Unit (Prof D Golinelli MD), Other, Ravenna, Italy; Department of Epidemiology (Prof A C Goulart PhD), University of São Paulo, São Paulo, Brazil; University of São Paulo, (Prof A C Goulart PhD); Department of Internal Medicine (A Goyal MD), Lerner Research Institute (X Liu PhD), Cleveland Clinic, Cleveland, OH, United States of America; Department of Toxicology (S Gupta MSc), Shriram Institute for Industrial Research, Delhi, India; Department of Public Health (B Gupta PhD), Torrens University Australia, Melbourne, VIC, Australia; Medical Research Unit (H Harapan PhD), Universitas Syiah Kuala (Syiah Kuala University), Banda Aceh, Indonesia; Department of Zoology and Entomology (A I Hasaballah PhD), Al-Azhar University, Cairo, Egypt; Department of Pharmacy (Prof M S Hasnain PhD), Palamau Institute of Pharmacy, Daltonganj, India; Gastrointestinal and Liver Diseases Research Center (S Hassanipour PhD), Caspian Digestive Disease Research Center (S Hassanipour PhD), Inflammatory Lung Diseases Research Center (N Rahmanian PhD), Guilan University of Medical Sciences, Rasht, Iran; Community-Oriented Nursing Midwifery Research Center (M Heidari PhD), Modeling in Health Research Center (A Mohammadian-Hafshejani PhD), Shahrekord University of Medical Sciences, Shahrekord, Iran; Department of Microbiology (K Hezam PhD), Taiz University, Taiz, Yemen; School of Medicine (K Hezam PhD), Nankai University, Tianjin, China; School of Dentistry (N Hoan DDS), Hanoi Medical University, Hanoi, Vietnam; School of Social Sciences (P Hoogar PhD), The Apollo University, Chittoor, India; Institute of Research and Development (Prof M Hosseinzadeh PhD), Duy Tan University, Da Nang, Vietnam; International Master Program for Translational Science (H Huynh BS), International Ph.D. Program in Medicine (L Minh MD), Research Center for Artificial Intelligence in Medicine (L Minh MD), Taipei Medical University, Taipei, Taiwan; School of Public Health (H Zhang MS), Peking University, Beijing, China; Department of International Health (H Zhang MS), Department of Epidemiology (T G Hundie MD), Johns Hopkins University, Baltimore, MD, United States of America (E Melese MD); Department of Health Promotion and Education (S Ibitoye PhD), Department of Obstetrics and Gynecology (O B Oghenetega MSc), College of Medicine (A P Okekunle PhD), Department of Medicine (O

V Olalusi MD), Faculty of Public Health (I I Olufadewa MHS), University of Ibadan, Ibadan, Nigeria; The National Centre for Remote and Rural Health and Care (A Ikiroma PhD), National Health Service (NHS) Scotland, Edinburgh, Scotland; Division of Infectious Diseases (K S Ikuta MD), Veterans Affairs Greater Los Angeles, Los Angeles, CA, United States of America; West Africa RCC (O S Ilesanmi PhD), Africa Centre for Disease Control and Prevention, Abuja, Nigeria; Department of Community Medicine (O S Ilesanmi PhD), Department of Medicine (A S Oguntade MSc), Department of Neurology (O V Olalusi MD), Department of Oral and Maxillofacial Surgery (A A Salami BDS), University College Hospital, Ibadan, Ibadan, Nigeria; Faculty of Medicine (I M Ilic PhD, Prof M M Santric-Milicevic PhD), School of Public Health and Health Management (Prof M M Santric-Milicevic PhD), University of Belgrade, Belgrade, Serbia; Department of Research and Innovation (A Iradukunda MD), ARNECH Research and Consulting Office, Bujumbura, Burundi; Department of Biotechnology (M A Isa PhD), Sharda University, Greater Noida, India; Department of Clinical Pharmacy & Pharmacy Practice (Prof N Ismail PhD), Asian Institute of Medicine, Science and Technology, Bedong, Malaysia; Malaysian Academy of Pharmacy, Puchong, Malaysia (Prof N Ismail PhD); Knowledge Translation Program (I O Iyamu MD), Centre for Health Evaluation and Outcome Sciences, Vancouver, BC, Canada; School of Population and Public Health (I O Iyamu MD), School of Nursing (A Pashaei MSc), University of British Columbia, Vancouver, BC, Canada; Department of Health Studies (K H Jacobsen PhD), University of Richmond, Richmond, VA, United States of America; Department of Leukemia (A Jain MD), The University of MD Anderson Cancer Center, Houston, TX, United States of America; Department of Health and Safety (A A Jairoun PhD), Dubai Municipality, Dubai, United Arab Emirates; The World Academy of Sciences UNESCO, Trieste, Italy (Prof M Jakovljevic PhD); Shaanxi University of Technology, Hanzhong, China (Prof M Jakovljevic PhD); School of Pharmaceutical Management (Prof M D Janodia PhD), IIHMR University, Jaipur, India; Department of Parasitology (A Javadi Mamaghani PhD), Shahid Beheshti University of Medical Sciences, Tehran, Iran; parasitology (A Javadi Mamaghani PhD), Tabriz University of Medical Sciences, Tabriz, Iran; Department of Public Health (A Jema MPH), Madda Walabu University, Goba, Ethiopia; Zoonoses Research Center (M Jokar DVM), Islamic Azad University, Karaj, Iran; Rothschild Foundation Hospital (Prof J B Jonas MD), Institute of Molecular and Clinical Ophthalmology Basel, Paris, France; Singapore Eye Research Institute (Prof J B Jonas MD), Singapore Eye Research Institute, Singapore, Singapore; Department of Economics (C E Joshua BSc), National Open University, Benin City, Nigeria; Department of Social Work (M Kabir PhD), Pabna University of Science and Technology, Pabna, Bangladesh; School of Public Health (Z Kabir PhD), University College Cork, Cork, Ireland; Department of Oral and Maxillofacial Pathology (V Kadashetti MDS), Department of Public Health Dentistry (Prof S M Kondlahalli MD), Krishna Vishwa Vidyapeeth (Deemed to be University), Karad, India; Department of Dermatology (F Kaliyadan MD), King Faisal University, Hofuf, Saudi Arabia; Faculty of Dentistry (K K Kanmodi MPH, A A Salami BDS), University of Puthisastra, Phnom Penh, Cambodia; Office of the Executive Director (K K Kanmodi MPH), Cephas Health Research Initiative Inc, Ibadan, Nigeria; Department of Community Medicine (S Kannan S MD), ESIC Medical College and Hospital Chennai, Chennai, India; School of Health Professions and Human Services (I M Karaye MD), Hofstra University, Hempstead, NY, United States of America; Department of Anesthesiology (I M Karaye MD), Montefiore Medical Center, Bronx, NY, United States of America; International Research Center of Excellence (G A Kayode PhD), Institute of Human Virology Nigeria, Abuja, Nigeria; Julius Centre for Health Sciences and Primary Care (G A Kayode PhD), Utrecht University, Utrecht, Netherlands; Amity Institute of Forensic Sciences (H Khajuria PhD, B P Nayak PhD), Amity Institute of Pharmacy (K Munjal PhD), Amity Institute of Public Health (M Shannawaz PhD), Amity University, Noida, India; College of Health Sciences (N Khalid PhD), Abu Dhabi University, Adu Dhabi,

United Arab Emirates; Halal Research Center of the Islamic Republic of Iran (IRI) (F Khamesipour PhD), Iran Food and Drug Administration, Tehran, Iran; Department of Epidemiology and Biostatistics (E A Khan MPH), Health Services Academy, Islamabad, Pakistan; Department of Clinical Pharmacy (Y H Khan PhD, T Mallhi PhD), Jouf University, Sakaka, Saudi Arabia; Population Science Department (M Khan PhD), Jatiya Kabi Kazi Nazrul Islam University, Mymensingh, Bangladesh; Department of Public Health (M Khan PhD), University of Sydney, Sydney, NSW, Australia (S R Okeke PhD); College of Health, Wellbeing and Life Sciences (K Khatab), Sheffield Hallam University, Sheffield, United Kingdom; College of Arts and Sciences (K Khatab), Ohio University, Zanesville, OH; Department of Biochemistry (F Khidri PhD), Liaquat University Of Medical and Health Sciences, Jamshoro, Pakistan; Department of Health Management and Economics (M Khosravi PhD), Qom University of Medical Sciences, Qom, Iran; Department of Public Health (J Khubchandani PhD), New Mexico State University, Las Cruces, NM, United States of America; Broad Institute of MIT and Harvard, Cambridge, MA, United States of America (M Kim MD); Samsung Advanced Institute for Health Sciences & Technology (SAIHST) (J Kim MD), Sungkyunkwan University, Seoul, South Korea; School of Traditional Chinese Medicine (Y Kim PhD), Xiamen University Malaysia, Sepang, Malaysia; School of Health Sciences (Prof A Kisa PhD), Kristiania University College, Oslo, Norway; Department of International Health and Sustainable Development (Prof A Kisa PhD), Tulane University, New Orleans, LA, United States of America; Department of Nursing and Health Promotion (S Kisa PhD), Oslo Metropolitan University, Oslo, Norway; Department of Physiology (S Komaki MD), Hamedan University of Medical Sciences, Hamedan, Iran; Department of Internal and Pulmonary Medicine (Prof P A Koul MD), Sheri Kashmir Institute of Medical Sciences, Srinagar, India; Kasturba Medical College, Manipal (S Koulmane Laxminarayana MD), Manipal Academy of Higher Education, Udupi, India; Department of Anthropology (Prof K Krishan PhD), Panjab University, Chandigarh, India; Department of Demography (Prof B Kuate Defo PhD), Department of Social and Preventive Medicine (Prof B Kuate Defo PhD), University of Montreal, Montreal, QC, Canada; Department of Mathematics (M Kuddus PhD), Department of Population Science and Human Resource Development (Prof M Rahman DrPH), University of Rajshahi, Rajshahi, Bangladesh; Atchabarov Scientific-Research Institute of Fundamental and Applied Medicine (M Kulimbet MSc), National Research Institute of Fundamental and Applied Medicine named after B. Atchabarov (A Zhumagaliuly MD), Kazakh National Medical University, Almaty, Kazakhstan; Center of Medicine and Public Health (M Kulimbet MSc), Asfendiyarov Kazakh National Medical University, Almaty, Kazakhstan; Geospatial Information Science and Engineering Hub (V Kumar PhD), Indian Institute of Technology, Mumbai, India; Centre for Studies in Economics and Planning (V Kumar PhD), Central University of Gujarat, Gandhinagar, India; Institute for Excellence in Health Equity (M Kumar PhD), The Center for Drug Use and HIV Research (CDUHR) (P Meylakhs PhD), School of Global Public Health (E K Peprah PhD), New York University, New York, NY, United States of America; Department of Psychiatry (M Kumar PhD), School of Public Health (R G Wamai PhD), University of Nairobi, Nairobi, Kenya; Indian Council of Medical Research, New Delhi, India (D K Lal MD); Faculty of Medicine (N Le MD), Department of General Medicine (V T Nguyen MD), Department of Internal Medicine (T H Tran MD), University of Medicine and Pharmacy at Ho Chi Minh City, Ho Chi Minh City, Vietnam (T T Le MD); Department of Cardiovascular Research (N Le MD), Methodist Hospital, Merrillville, IN, United States of America; Department of Precision Medicine (Prof S Lee MD), Sungkyunkwan University, Suwon-si, South Korea; Department of Public Health (T L Lerango MPH), Department of Pediatrics and Child Health Nursing (M M Sibhat MSc), Dilla University, Dilla, Ethiopia; Department of Health Promotion and Health Education (M Li PhD), National Taiwan Normal University, Taipei, Taiwan; Department of Quantitative Health Science (X Liu PhD), Department of Neonatology (I

Qattea MD), Case Western Reserve University, Cleveland, OH, United States of America; Center for Evidence-Based Medicine and Clinical Research (R Liu PhD), Hubei University of Medicine, Shiyan, China; Department of Chemistry (H I M. Amin PhD), Salahaddin University-Erbil, Erbil, Iraq; Department of Medical Biochemical Analysis (H I M. Amin PhD), Cihan University-Erbil, Erbil, Iraq; Centre for Public Health and Wellbeing (Z Ma PhD), University of the West of England, Bristol, United Kingdom; Rama Medical College Hospital and Research Centre, Uttar Pradesh, India (K Malhotra MBBS); Institute of Applied Health Research (K Malhotra MBBS), University of Birmingham, Birmingham, United Kingdom; School of Public Health (K Malhotra MBBS), Centre for Heart Rhythm Disorders (J Noubiap MD), University of Adelaide, Adelaide, SA, Australia; Rabigh Faculty of Medicine (Prof A Malik PhD), King Abdulaziz University, Jeddah, Saudi Arabia; The University of Lahore, (Prof A Malik PhD); Department of Electrical Engineering (I Malik PhD), Prince Sattam bin Abdulaziz University, Al Kharj, Saudi Arabia; Research Department (B P Marasini PhD, A Pandey MPH), Nepal Health Research Council, Kathmandu, Nepal; Department of Biotechnology (B P Marasini PhD), Tribhuvan University, Kathmandu, Nepal; Department of Infectious Diseases (B A Martinez-Guerra MSc), Instituto Nacional de Nutrición Salvador Zubirán (Salvador Zubiran National Institute of Medical Sciences and Nutrition), Mexico City, Mexico; Fortaleza Campus (F R Martins-Melo PhD), Federal Institute of Education, Science and Technology of Ceará, Fortaleza, Brazil; Department of Nutrition and Dietetics (M Martorell PhD), Centre for Healthy Living (M Martorell PhD), University of Concepción, Concepción, Chile; Faculty of Humanities and Health Sciences (Prof R R Marzo MD), Curtin University, Sarawak, Malaysia; Jeffrey Cheah School of Medicine and Health Sciences (Prof R R Marzo MD), School of Pharmacy (S Shrestha PharmD, Y Wong PhD), Monash University, Subang Jaya, Malaysia; Department of General Medicine (N Mathur MD), Geetanjali Medical College and Hospital, UDAIPUR, India; Research & Innovation Center (Prof Z A Memish MD), Ministry of Health, Riyadh, Saudi Arabia; Peru Country Office (W Mendoza MD), United Nations Population Fund (UNFPA), Lima, Peru; Comprehensive Cancer Center (T J Meretoja MD), Helsinki University Hospital, Helsinki, Finland; Department of Virology (F Zakham PhD), University of Helsinki, Helsinki, Finland (T J Meretoja MD); University Centre Varazdin (T Mestrovic PhD), University North, Varazdin, Croatia; Laboratory for Comparative Social Research (P Meylakhs PhD), National Research University Higher School of Economics, St. Petersburg, Russia; Northwestern University, Chicago, IL, United States of America (L Mhlanga PhD); South African Centre for Epidemiological Modelling and Analysis (SACEMA) (L Mhlanga PhD), Department of Epidemiology (J L Tamuzi MSc), Department of Industrial Psychology (E Teye-Kwadjo PhD), Stellenbosch University, Cape Town, South Africa; National Cancer Registry (I Michalek PhD), Pathology Department (I Michalek PhD), Maria Sklodowska-Curie National Research Institute of Oncology, Warsaw, Poland; Multidisciplinary Department of Medical-Surgical and Dental Specialties (G Minervini PhD), Univerity of Campania "Luigi Vanvitelli", Naples, Italy; Saveetha Dental College and Hospitals (G Minervini PhD, M Tovani-Palone PhD), Department of Pharmacology (S Muthupandian PhD), Centre of Molecular Medicine and Diagnostics (COMManD) (Prof S Patil PhD), Saveetha University, Chennai, India; Institute of Addiction Research (ISFF) (B Moazen MSc), Frankfurt University of Applied Sciences, Frankfurt am Main, Germany; Molecular Biology Unit (N S Mohamed MSc), Bio-Statistical and Molecular Biology Department (N S Mohamed MSc), Sirius Training and Research Centre, Khartoum, Sudan; Department of Pharmaceutical Sciences (S Mohammed PhD), Notre Dame of Maryland University, Baltimore, MD, United States of America; Department of Pharmacy (S Mohammed PhD), Mizan-Tepi University, Mizan, Ethiopia; Clinical Epidemiology and Public Health Research Unit (L Monasta DSc), Burlo Garofolo Institute for Maternal and Child Health, Trieste, Italy; AI & Cyber Futures Institute (M Moni PhD), Charles Sturt University, Bathurst, NSW, Australia; Department

of Ophthalmology & Vision Science (F Montazeri MD), University of California Davis, Sacramento, CA, United States of America; Department of Epidemiology and Biostatistics (Y Moradi PhD), Department of Microbiology (M Sedighi PhD), Kurdistan University of Medical Sciences, Sanandaj, Iran; Groningen Research Institute of Pharmacy (Prof S Mubarik PhD), University Medical Center Groningen (Prof M J Postma PhD), University of Groningen, Groningen, Netherlands; Department of Epidemiology and Biostatistics (Prof S Mubarik PhD), Wuhan University, Wuhan, China; Department of Surgery (G D Mukoro MD), Ahmadu Bello University Teaching Hospital, Zaria, Nigeria; Department of Surgery (F Mulita PhD, G Verras MD), General University Hospital of Patras, Patras, Greece; Faculty of Medicine (F Mulita PhD), University of Thessaly, Larissa, Greece; Department of Community and Global Health (Y Munkhsaikhan MD), The University of Tokyo, Tokyo, Japan; Department of Pediatrics & Pediatric Pulmonology (Prof G Mustafa MD), Institute of Mother & Child Care, Multan, Pakistan; Prince Fahad bin Sultan Chair for Biomedical Research (S Muthupandian PhD), University of Tabuk, Tabuk, Saudi Arabia; Research and Analytics Department (A J Nagarajan MTech), Initiative for Financing Health and Human Development, Chennai, India; Department of Research and Analytics (A J Nagarajan MTech), Bioinsilico Technologies, Chennai, India; Department of Computer Science (P Naghavi MS), University of Illinois Urbana-Champaign, Urbana, IL, United States of America; Department Health Services Research (G Naik MPH), Department of Radiology (M Tanwar MD), University of Alabama at Birmingham, Birmingham, AL, United States of America; Faculty of Pharmacy (F Nainu PhD), Hasanuddin University, Makassar, Indonesia; Department of Community Medicine (S N K Navaratna MD), University of Peradeniya, Kandy, Sri Lanka; Postgraduate Institute of Medicine (S N K Navaratna MD), University of Colombo, Colombo, Sri Lanka; Department of Biotechnology (M Naveed PhD), University of Central Punjab, Lahore, Pakistan; School of Pharmacy (S O Nduaguba PhD), West Virginia University, Morgantown, WV, United States of America; Applied Cellular and Molecular Research Center (M Nematollahi PhD), Kerman University of Medical Sciences, Kerman, Iran; Department of Public Health (G Nguefack-Tsague PhD), University of Yaoundé I, Yaoundé, Cameroon; Cardiovascular Research Department (H Q Nguyen MD), Methodist Hospital, Merrillville, IL, United States of America; International Islamic University Islamabad, Islamabad, Pakistan (R K Niazi PhD); Institute for Mental Health Policy Research (Y T Nigatu PhD), Centre for Addiction and Mental Health, Toronto, ON, Canada; Department of Nephrology and Hypertension (N Nikravangolsefid MD), Department of Radiology (F Nugen PhD), Department of Informatics and Radiology (S Vahdati MD), Mayo Clinic, Rochester, MN, United States of America; Department of Public Health (V Niranjan PhD), HSE Ireland, Dublin, Ireland; Department of Public Health (V Niranjan PhD), UNICAF, Larnaca, Cyprus; Technical Department (C A Nnaji PhD), South African Medical Research Council, Cape Town, South Africa; School of Public Health and Family Medicine (C A Nnaji PhD), Department of Medicine (Prof F Thienemann PhD), University of Cape Town, Cape Town, South Africa; Department of Statistics (S Noor MS), Shahjalal University of Science and Technology, Sylhet, Bangladesh; School of Medicine (N Not applicable PhD), Xiamen University, Xiamen, China; Department of Paediatrics (C A Nri-Ezedi PhD), Nnamdi Azikiwe University, Awka, Nigeria; School of Information (F Nugen PhD), University of California Berkeley, Berkeley, CA, United States of America; School of Nursing (J Nutor PhD), University of California San Francisco, San Francisco, CA, United States of America; Center of Excellence in Reproductive Health Innovation (CERHI) (C I Nzoputam MPH), University of Benin, Benin City, Nigeria; Department of Physiology (O J Nzoputam PhD), University of Benin, Edo, Nigeria; Department of Physiology (O J Nzoputam PhD), Benson Idahosa University, Benin City, Nigeria; Institute of Cardiovascular Science (A S Oguntade MSc), Center for Clinical Microbiology (Prof A Zumla PhD), University College London, London, United Kingdom; Department of Food and Nutrition (A P Okekunle

PhD), Seoul National University, Seoul, South Korea; School of Pharmacy (O C Okonji MSc), University of the Western Cape, Cape Town, South Africa; Department of Psychiatry and Behavioural Neurosciences (A T Olagunju MD), McMaster University, Hamilton, ON, Canada; Department of Psychiatry (A T Olagunju MD), University of Lagos, Lagos, Nigeria; Department of Population and Community Health (B O Olakunde PhD), University of North Texas Health Science Center, Fort Worth, TX, United States of America; Department of Nursing Science (M I Olatubi PhD), Bowen University, Iwo, Nigeria; Slum and Rural Health Initiative Research Academy (I I Olufadewa MHS), Slum and Rural Health Initiative, Ibadan, Nigeria; Department of Pharmacotherapy and Pharmaceutical Care (M Ordak PhD), Department of Biochemistry and Pharmacogenomics (M Zielińska MPharm), Medical University of Warsaw, Warsaw, Poland; Sickle Cell Unit (Prof V N Orish PhD), Ho Teaching Hospital, Ho, Ghana; Department of Infectious Diseases (E Ortiz-Brizuela MD), Salvador Zubiran National Institute of Medical Sciences and Nutrition, Mexico City, Mexico; Dirección de Prestaciones Económicas y Sociales (E Ortiz-Brizuela MD), Instituto Mexicano del Seguro Social, Ciudad de México, Mexico; School of Medicine (U L Osuagwu PhD), Western Sydney University, Bathurst, NSW, Australia; Department of Optometry and Vision Science (U L Osuagwu PhD), University of KwaZulu-Natal, KwaZulu-Natal, South Africa; Division of Infectious Diseases (Prof A Ouyahia PhD), University Hospital of Setif, Setif, Algeria; Department of Respiratory Medicine (Prof M P P A DNB), Jagadguru Sri Shivarathreeswara University, Mysore, India; Research Institute for Medicines (C Palladino PhD), Universidade de Lisboa (University of Lisbon), Lisbon, Portugal; Research Department (A Pandey MPH), Public Health Research Society Nepal, Kathmandu, Nepal; Department of Neurology (L D Panos MD), University of Bern, Bern, Switzerland; Department of Neurology (L D Panos MD), University of Cyprus, Nicosia, Cyprus; Alexander von Humboldt Institute of Tropical Medicine (J L Paredes MD), Cayetano Heredia University, Lima, Peru; Department of Community Medicine (P P Parija MD), All India Institute of Medical Sciences, Jammu, India; Department of Epidemiology and Community Health (R R Parikh MD), University of Minnesota, Minneapolis, MN, United States of America; Department of Research and Training (S K Patel PhD), Population Council, New Delhi, India; Research Consultancy (A R Pathan PhD), Author Gate Publications, Malegaon, India; College of Dental Medicine (Prof S Patil PhD), Roseman University of Health Sciences, South Jordan, UT, United States of America; Department of Genetics (S Pawar PhD), Yale University, New Haven, CT, United States of America; Australian Institute of Health Innovation (P Peprah MSc), Macquarie University, Sydney, NSW, Australia; Institute of Collective Health (Prof M Pereira PhD), Federal University of Bahia, Salvador, Brazil; Department of Biology (Prof S Perna PhD), College of Health and Sport Sciences (A G Vaithinathan MSE), University of Bahrain, Zallaq, Bahrain; Department of Statistics and Econometrics (I Petcu PhD), Bucharest University of Economic Studies, Bucharest, Romania; Department of Internal Medicine (H Pham MD), Weiss Memorial Hospital, Chicago, IL, United States of America; Basic Medical Sciences Department (J D Pillay PhD), Durban University of Technology, Durban, South Africa; Department of Data Management and Analysis (R Poluru PhD), The International Clinical Epidemiology Network (INCLEN) Trust International, New Delhi, India; Center of Excellence in Higher Education for Pharmaceutical Care Innovation (Prof M J Postma PhD), Universitas Padjadjaran (Padjadjaran University), Bandung, Indonesia; Non-communicable Diseases Research Center (N Pourtaheri PhD), Bam University of Medical Sciences, Bam, Iran; Humanities and Social Sciences (Prof J Pradhan PhD), National Institute of Technology Rourkela, Rourkela, India; Center for AIDS Health and Disparity Research, Meharry Medical College (P Prakash PhD), Not Listed, Nashville, TN, TN, United States of America; Department of Medical Oncology (Prof T N Prakasham DM), Nizam's Institute of Medical Sciences, Hyderabad, India; Health Sciences Department (D R A Pribadi MSc), Muhammadiyah University of Surakarta, Sukoharjo, Indonesia; Research and

Development Coordination (I Rafique PhD), National Institutes of Health, Islamabad, Pakistan; Department of Medical Laboratory Technologies (Prof F Rahim PhD), Al-Noor Center of Research and Innovation (Prof F Rahim PhD), Alnoor University, Mousl, Iraq; Future Technology Research Center (A Rahmani PhD), National Yunlin University of Science and Technology, Yunlin, Taiwan; Department of Public Health (V Rahmanian PhD), Torbat Jam Faculty of Medical Sciences, Torbat Jam, Iran; Department of Community Medicine (S Rajaa MD), Employees' State Insurance Model Hospital, Chennai, India; Department of Cardiology (Prof M M Ramadan PhD), Department of Hygiene and Zoonoses (H Ramadan PhD), Faculty of Pharmacy (Prof M A Saleh PhD), Mansoura University, Mansoura, Egypt; Department of Radiology (S Ramasamy MD), Stanford University, Stanford, CA, United States of America; Translational Health Research Institute (K Rana PhD), Western Sydney University, Sydney, NSW, Australia; Research Department (C L Ranabhat PhD), Eastern Scientific LLC, Richmond, KY, United States of America; Department of Health Promotion and Administration (C L Ranabhat PhD), Eastern Kentucky University, Richmond, KY, United States of America; Department of Oral Pathology, Microbiology and Forensic Odontology (S Rao MDS), Sharavathi Dental College and Hospital, Shimogga, India; Department of Family Medicine (Prof D Rathish MPH), Department of Community Medicine (N D Wickramasinghe MD), Rajarata University of Sri Lanka, Anuradhapura, Sri Lanka; Department of Global Health Policy (S K Rauniyar PhD), University of Tokyo, Tokyo, Japan; Academic Public Health England (Prof S Rawaf MD), Public Health England, London, United Kingdom; Department of Biological Sciences (Prof E M M Redwan PhD), King Abdulaziz University, Jeddah, Egypt; Department of Protein Research (Prof E M M Redwan PhD), Research and Academic Institution, Alexandria, Egypt; Department of Epidemiology and Biostatistics (Prof M Rezaeian PhD), Rafsanjan University of Medical Sciences, Rafsanjan, Iran; Department of Pharmacology and Toxicology (Prof J A B Rodriguez PhD), University of Antioquia, Medellin, Colombia; Warwick Medical School (Prof J A B Rodriguez PhD), University of Warwick, Coventry, United Kingdom; Technical Department (K Rotimi MSc), Malaria Consortium, Abuja, Nigeria; Department of Public Health Pharmacy (K Rotimi MSc), West African Postgraduate College of Pharmacists, Lagos, Nigeria; Department of Biochemistry and Food Analysis (N Roy PhD), Patuakhali Science and Technology University, Patuakhali, Bangladesh; Department of Internal Medicine (G M Rwegerera MD), University of Botswana, Gaborone, Botswana; Department of Medicine (C J Sabet MA), Georgetown University, Washington, DC, United States of America; Department of Pharmaceutical Chemistry (Prof M Saeb PhD), International Medical University, Gdańsk, Poland; Clinical and Biomedical Research Center (Prof U Saeed PhD), Foundation University Islamabad, Islamabad, Pakistan; International Center of Medical Sciences Research (ICMSR), Islamabad, Pakistan (Prof U Saeed PhD); Faculty of Medicine, Bioscience and Nursing (S Z Safi PhD), MAHSA University, Selangor, Malaysia; Interdisciplinary Research Centre in Biomedical Materials (IRCBM) (S Z Safi PhD), COMSATS Institute of Information Technology, Lahore, Pakistan; Department of Community & Family Medicine (S S Sahoo MD), Department of Radiodiagnosis (P Singh MD), All India Institute of Medical Sciences, Bathinda, India; Department of Psychiatry (Z Saif MBA), Ministry of Health, Manama, Bahrain; Department of Statistics (M R Sajid PhD), University of Gujrat, Gujrat, Pakistan; Department of Health Education & Promotion (Prof L Salehi PhD), A.C.S. Medical College and Hospital, Karaj, Iran; Research Center for Health, Safety and Environment (Prof L Salehi PhD), Alborz University of Medical Sciences, Karaj, Iran; Department of Entomology (A M Samy PhD), Medical Ain Shams Research Institute (MASRI) (A M Samy PhD), Ain Shams University, Cairo, Egypt; Department of Pediatrics (Prof R K Sanjeev MD), Sree Balaji Medical College and Hospital, Chennai, India; Indira Gandhi Medical College and Research Institute, Puducherry, India (A Saravanan MD); Nuffield Department of Medicine (B Sartorius PhD), University of Oxford, Oxford, United

Kingdom; UGC Centre of Advanced Study in Psychology (M Satpathy PhD), Utkal University, Bhubaneswar, India; Udyam-Global Association for Sustainable Development, Bhubaneswar, India (M Satpathy PhD); Department of Public Health Sciences (M Sawhney PhD), University of North Carolina at Charlotte, Charlotte, NC, United States of America; Department of Public Health (B Semagn MPH), Debre Berhan University, Debre Berhan, Ethiopia; Department of Medicine and Surgery (Y Sethi MBBS), Government Doon Medical College, Dehradun, India; National Heart, Lung, and Blood Institute (A Seylani BS), National Institute of Health, Rockville, MD, United States of America; Department of Microbiology (P A Shah MBBS), Rajiv Gandhi University of Health Sciences, Bangalore, India; Independent Consultant, Karachi, Pakistan (M A Shaikh MD); Department of Pathobiology (M Shamshirgaran PhD), Shahid Bahonar University of Kerman, Kerman, Iran; Department for Evidence-based Medicine and Evaluation (A Sharifan PharmD), University for Continuing Education Krems, Krems, Austria; Department of Biomedical Sciences (J Sharifi-Rad PhD), Korea University, Seoul, South Korea; K S Hegde Medical Academy (Prof M Shetty MD), Nitte University, Mangalore, India; Department of Public Health (D Shiferaw MPH), Dambi Dollo University, Dembi Dollo, Ethiopia; Department of Veterinary Public Health and Preventive Medicine (A Shittu MSc), Usmanu Danfodiyo University, Sokoto, Sokoto, Nigeria; Research and Academics, Pharmacy (S Shrestha PharmD), Other, Bhaktapur, Nepal; Department of Medical Microbiology and Infectious Diseases (E E Siddig MD), Erasmus University, Rotterdam, Netherlands; Research Faculty (M J Siedner MD), Africa Health Research Institute, Durban, South Africa; School of Medicine (Prof J A Singh MD), Henry JN Taub Department of Emergency Medicine (Prof L Szarpak PhD), Baylor College of Medicine, Houston, TX, United States of America; Medicine Service (Prof J A Singh MD), US Department of Veterans Affairs (VA), Houston, TX, United States of America; Department of Pharmacology (H Singh DM), Government Medical College and Hospital, Chandigarh, India; Department of Internal Medicine (R Sinto MD), University of Indonesia, Jakarta Pusat, Indonesia; Department of Internal Medicine (R Sinto MD), Dr. Cipto Mangunkusumo National Hospital, Jakarta Pusat, Indonesia; Department of Infectious Diseases and Epidemiology (A A Skryabina MD), Pirogov Russian National Research Medical University, Moscow, Russia; Department of Infectious Diseases (Prof A Sokhan PhD), Kharkiv National Medical University, Kharkiv, Ukraine; Clinical line (Prof A Sokhan PhD), Other, Vienna, Austria; Department of Biochemistry (S Solanki MD), American University of Integrative Sciences, Bridgetown, Barbados; Department of Systemic Pathology (R Solanki MD), Touro College of Osteopathic Medicine, Middletown, NY, United States of America; Department of Pathology (R Solanki MD), American University of the Caribbean School of Medicine, Cupecoy, Saint Martin; Department of Medicine (S K Sulaiman MD), Yobe State University Teaching Hospital, Yobe, Nigeria; Department of Clinical Research and Development (Prof L Szarpak PhD), LUXMED Group, Warsaw, Poland; Department of Pharmacology (S T Y MD), All India Institute of Medical Sciences, Deoghar, India; School of Dentistry and Oral Health (S K Tadakamadla PhD), Griffith University, Gold Coast, QLD, Australia; Living Systems Institute (Y Taheri Abkenar PharmD), Faculty of Health and Life Sciences (A Udoh PhD), University of Exeter, Exeter, United Kingdom; Department of Environmental, Agricultural and Occupational Health (J Taiba MPH), University of Nebraska Medical Center, Omaha, NE, United States of America; Sri Ramachandra Medical College and Research Institute, Chennai, India (J Taiba MPH); Department of Dermatology (M Tampa PhD), Carol Davila University of Medicine and Pharmacy, Bucharest, Romania; Department of Dermato-Venereology (M Tampa PhD), Dr. Victor Babes Clinical Hospital of Infectious Diseases and Tropical Diseases, Bucharest, Romania; Department of Medicine (J L Tamuzi MSc), Northlands Medical Group, Omuthiya, Namibia; Department of Surgery (K Tan PhD), Saw Swee Hock School of Public Health (Prof S Yi PhD), National University of Singapore, Singapore, Singapore; Egas

Moniz School of Health and Science (Prof N Taveira PhD), Egas Moniz Cooperativa de Ensino Superior CRL (University Institute "Egas Moniz"), Monte da Caparica, Portugal; Faculty of Pharmacy (Prof N Taveira PhD), University of Lisbon, Lisbon, Portugal; Department of Clinical Pharmacy (G Teklay MSc), Mekelle University, Mekelle, Ethiopia; Department of Psychology (E Teye-Kwadjo PhD), University of Ghana, Legon, Ghana; School of Humanities and Social Sciences (R Thakur PhD), Indian Institute of Technology Mandi, Mandi, India; Department of Pharmacology (P Thangaraju MD), All India Institute of Medical Sciences, Raipur, India; Eastern Health Clinical School (R Thapa PhD), Monash University, Melbourne, VIC, Australia; Other, (R Thapa PhD); Department of Internal Medicine (Prof F Thienemann PhD), University of Zürich, Zurich, Switzerland; Department of Global Health (Prof J Thomas PhD), Sustainable Policy Solutions Foundation, South Yatta, VIC, Australia; Department of Business Analytics (T H Tran MD), University of Massachusetts Dartmouth, Dartmouth, MA, United States of America; School of Medicine and Dentistry (M T N Tran PhD), Queensland University of Technology, GoldCoast, QLD, Australia; Health Informatics Department (M T N Tran PhD), Hanoi Medical University, Ha Noi, Vietnam; Mbarara University of Science and Technology, Mbarara, Uganda (A C Tsai MD); Department of Allied Health Sciences (I Ullah PhD), Iqra University Chak Shahzad Campus, Islamabad, Pakistan; Natural and Medical Sciences Research Center (A Ullah MS), University of Nizwa, Nizwa, Oman; Medical Genomics Research Department (Prof M Umair PhD), King Abdullah International Medical Research Center, Riyadh, Saudi Arabia; Department of Life Sciences (Prof M Umair PhD), University of Management and Technology, Lahore, Pakistan; UKK Institute, Tampere, Finland (Prof T J Vasankari PhD); Faculty of Medicine and Health Technology (Prof T J Vasankari PhD), Tampere University, Tampere, Finland; Department of Physiotherapy (J H Villafañe PhD), Universidad Europea de Madrid (European University of Madrid), Villaviciosa de Odón, Spain; Department of Cultures, Societies and Global Studies (R G Wamai PhD), Northeastern University, Boston, MA, United States of America; School of Life Course and Population Sciences (Prof Y Wang PhD), King's College London, London, United Kingdom; Key Laboratory of Computer-Aided Drug Design (M Waqas PhD), Guangdong Medical University, Dongguan, China; Department of Biotechnology and Genetic Engineering (M Waqas PhD), Hazara University Mansehra, Mansehra, Pakistan; Cardiology Department (Prof R G Weintraub MB), Royal Children's Hospital, Melbourne, VIC, Australia; Department of Critical Care and Neurosciences (Prof R G Weintraub MB), Murdoch Childrens Research Institute, Parkville, VIC, Australia; Competence Center of Mortality-Follow-Up of the German National Cohort (R Westerman DSc), Federal Institute for Population Research, Wiesbaden, Germany; National Data Management Center for Health (NDMC) (M A Woldekidan MPH), Ethiopian Public Health Institute, Addis Ababa, Ethiopia; School of Public Health (X Wu MPH), Fudan University, Shenzhen, China; Department of Basic Medical Sciences (S Yaghoubi PhD), Neyshabur University of Medical Sciences, Neyshabur, Iran; Biostatics, Epidemiology, and Science Computing Department (S Yezli PhD), King Faisal Specialist Hospital & Research Center, Riyadh, Saudi Arabia; KHANA Center for Population Health Research, Phnom Penh, Cambodia (Prof S Yi PhD); Department of Health Management (A Yiğit PhD), Süleyman Demirel Üniversitesi (Süleyman Demirel University), Isparta, Turkiye; Department of Epidemiology (D Yin DrPH), Xuzhou Medical University, Xuzhou, China; Pharmacy Department (Y Yismaw MSc), Alkan Health Science, Business and Technology College, Bahir Dar, Ethiopia; Department of Pediatrics (Prof D Yon MD), Kyung Hee University, Seoul, South Korea; Department of Biostatistics (Prof N Yonemoto PhD), University of Toyama, Toyama, Japan; Department of Public Health (Prof N Yonemoto PhD), Juntendo University, Tokyo, Japan; Faculty of Medicine and Health Sciences (F Zakham PhD), Hodeidah University, Hodeidah, Yemen; School of Public Policy and Administration (J Zhang BA), Xi 'an Jiaotong University, Xi'an, China; College of Traditional Chinese

Medicine (H Zhao MD), Hebei University, Baoding, China; School of Public Health and Emergency Management (B Zhu PhD), Southern University of Science and Technology, Shenzhen, China; The Second Clinical Medical School (Q Zhuang MD), The College of Public Health (Q Zhuang MD), Nanjing Medical University, Nanjing, China; School of Public Health (L Zihao MPH), Bengbu Medical College, Bengbu, China; NIHR-Biomedical Research Centre (NIHR-BRC) (Prof A Zumla PhD), University College London Hospitals, London, United Kingdom

Authors' Contributions

Managing the overall research enterprise

Aleksandr Aravkin, Sam Bryne, Simon Hay, Hmwe H Kyu, Christopher JL Murray, Mohsen Naghavi, Amanda Novotney, Austin Schumacher, and Peng Zheng

Writing the first draft of the manuscript

Austin Carter, Deepa Jahagirdar, Emmanuel Mpolya, Hmwe H Kyu, Khai Hoan Tram, Magdalene Walters, and Meixin Zhang

Primary responsibility for applying analytical methods to produce estimates

Austin Carter, Tahvi Frank, Jiawei He, Deepa Jahagirdar, Christine Lin, Erin May, Jianing Ma, Spencer Pease, Magdalene Walters, and Meixin Zhang

Primary responsibility for seeking, cataloguing, extracting, or cleaning data; designing or coding figures and tables

Edmond Brewer, Sam Bryne, Haley Comfort, Jianing Ma, and Avina Vongpradith

Designing or coding figures and tables

Edmond Brewer, Austin Carter, Regina-Mae Villanueva Dominguez, Dylan Lasher, Megan Verma, Avina Vongpradith, and Meixin Zhang

Providing data or critical feedback on data sources

Hedayat Abbastabar, Parsa Abdi, Meriem Abdoun, Jeza Muhamad Abdul Aziz, Richard Gyan Aboagye, Eman Abu-Gharbieh, Salahdein Aburuz, Oyelola A Adegboye, Charles Oluwaseun Adetunji, Juliana Bunmi Adetunji, Qorinah Estiningtyas Sakilah Adnani, Fatemeh Afrashteh, Saira Afzal, Shahin Aghamiri, Bright Opoku Ahinkorah, Aqeel Ahmad, Shahzaib Ahmad, Ali Ahmed, Ayman Ahmed, Haroon Ahmed, Syed Anees Ahmed, Gizachew Taddesse Akalu, Karolina Akinosoglou, Hanadi Al Hamad, Omar Ali Mohammed Al Zaabi, Samer O Alalalmeh, Fahad Mashhour Alanezi, Daniel Shewaye Alayu, Seyedeh Yasaman Alemohammad, Abid Ali, Mohammed Usman Ali, Waad Ali, Joseph Uy Almazan, Diala Altwalbeh, Reza Amani, Tewodros Getnet Amera, Ganiyu Adeniyi Amusa, Abhishek Anil, Abdul-Azeez Adeyemi Anjorin, Carl Abelardo T Antonio, Saleha Anwar, Geminn Louis Carace Apostol, Aleksandr Y Aravkin, Brhane Berhe Aregawi, Mulusew A Asemahagn, Mubarek Yesse Ashemo, Marvellous O Asika, Maha Moh'd Wahbi Atout, Avinash Aujayeb, Hamzeh Awad, Beatriz Paulina Ayala Quintanilla, Sina Azadnajafabad, Darshan B B, Muhammad Badar, Abdulaziz T Bako, Wondu Feyisa Balcha, Kiran Bam, Hiba Jawdat Barqawi, Zarrin Basharat, Hameed Akande Bashiru, Afisu Basiru, Mohammad-Mahdi Bastan, Kavita Batra, Ravi Batra, Tahmina Begum, Maryam Beiranvand, Melaku Ashagrie Belete, Alice A Beneke, Azizullah Beran, Alemshet Yirga Berhie, Amiel Nazer C Bermudez, Robert S Bernstein, Kebede A Beyene, Nikha Bhardwaj, Pankaj Bhardwaj, Ajay Nagesh Bhat, Gurjit Kaur Bhatti, Aadam Olalekan Bodunrin,

Hamed Borhany, Colin Stewart Brown, Danilo Buonsenso, Katrin Burkart, Yasser Bustanji, Chao Cao, Muthia Cenderadewi, Joshua Chadwick, Chiranjib Chakraborty, Rama Mohan Chandika, Akhilanand Chaurasia, Guangjin Chen, Dinh-Toi Chu, Isaac Sunday Chukwu, Eric Chung, Haley Comfort, Rosa A S Couto, Silvia Magali Cuadra-Hernández, Omid Dadras, Tukur Dahiru, José das Neves, Nihar Ranjan Dash, Mohsen Dashti, Kebede Deribe, Don C Des Jarlais, Hardik Dineshbhai Desai, Sameer Dhingra, Daniel Diaz, Deepa Dongarwar, Ojas Prakashbhai Doshi, Viola Savy Dsouza, Senbagam Duraisamy, Arkadiusz Marian Dziedzic, Hisham Atan Edinur, Ferry Efendi, Temitope Cyrus Ekundayo, Iman El Sayed, Muhammed Elhadi, Ugochukwu Anthony Eze, Ali Fatehizadeh, Nelsensius Klau Fauk, Nuno Ferreira, Florian Fischer, Morenike Oluwatoyin Folayan, Tahvi D Frank, Sridevi G, Muktar A Gadanya, Aravind P Gandhi, Mohammad Arfat Ganiyani, Mesfin Gebrehiwot, Motuma Erena Getachew, Keyghobad Ghadiri, Afsaneh Ghasemzadeh, Ahmad Ghashghaee, Nasim Gholizadeh, Mahsa Ghorbani, Mahaveer Golechha, Davide Golinelli, Alessandra C Goulart, Anmol Goyal, Mesay Dechasa Gudeta, Bhawna Gupta, Sapna Gupta, Arief Hargono, Md Saquib Hasnain, Shoaib Hassan, Soheil Hassanipour, Mbuzeleni Mbuzeleni Hlongwa, Mehdi Hosseinzadeh, Hong-Han Huynh, Segun Emmanuel Ibitoye, Kevin S Ikuta, Irena M Ilic, Jeffrey W Imai-Eaton, Vinothini J, Deepa Jahagirdar, Akhil Jain, Ammar Abdulrahman Jairoun, Mihajlo Jakovljevic, Manthan Dilipkumar Janodia, Amirreza Javadi Mamaghani, Ali Kabir, Vidya Kadashetti, Suthanthira Kannan S, Arman Karimi Behnagh, Gbenga A Kayode, Himanshu Khajuria, Nauman Khalid, Anees Ahmed Khalil, Faham Khamesipour, Ejaz Ahmad Khan, Gulfaraz Khan, Mohammad Jobair Khan, Feriha Fatima Khidri, Zahra Khorrami, Majid Khosravi, Sezer Kisa, Somayeh Komaki, Shivakumar KM Marulasiddaiah Kondlahalli, Mukhtar Kulimbet, Vishnutheertha Kulkarni, Rakesh Kumar, Vijay Kumar, Dharmesh Kumar Lal, Ming-Chieh Li, Stephen S Lim, Liknaw Workie Limenh, Christine Lin, Runben Liu, Rakesh Lodha, Jianing Ma, Zheng Feei Ma, Azeem Majeed, Elaheh Malakan Rad, Ahmad Azam Malik, Iram Malik, Tauqeer Hussain Mallhi, Bishnu P Marasini, Francisco Rogerlândio Rogerlândio Martins-Melo, Miquel Martorell, Roy Rillera Marzo, Navgeet Mathur, Erin A May, Hadush Negash Meles, Endalkachew Belayneh Melese, Ziad Ahmed Memish, Walter Mendoza, Ritesh G Menezes, Tomislav Mestrovic, Peter Meylakhs, Laurette Mhlanga, Giuseppe Minervini, Babak Moazen, Sakineh Mohammad-Alizadeh-Charandabi, Hussen Mohammed, Salahuddin Mohammed, Fateme Montazeri, Maryam Moradi, Yousef Moradi, Vincent Mougin, Emmanuel A Mpolya, George Duke Mukoro, Kavita Munjal, B.V. Murlimanju, Christopher J L Murray, Saravanan Muthupandian, Mohsen Naghavi, Pirouz Naghavi, Gurudatta Naik, Firzan Nainu, Mohammad Sadeq Najafi, Samidi Nirasha Kumari Navaratna, Biswa Prakash Nayak, Georges Nguefack-Tsague, Hien Quang Nguyen, Nasrin Nikravangolsefid, Nawsherwan Not applicable, Chisom Adaobi Nri-Ezedi, Fred Nugen, Chimezie Igwegbe Nzoputam, Ogochukwu Janet Nzoputam, Kehinde O Obamiro, Ismail A Odetokun, Ayodipupo Sikiru Oguntade, Osaretin Christabel Okonji, Andrew T Olagunju, Oladotun Victor Olalusi, Abdulahi Opejin, Michal Ordak, Verner N Orish, Edgar Ortiz-Brizuela, Uchechukwu Levi Osuagwu, Jagadish Rao Padubidri, Ashok Pandey, Leonidas D Panos, Jose L Paredes, Romil R Parikh, Ava Pashaei, Aslam Ramjan Pathan, Spencer A Pease, Emmanuel K Peprah, Marcos Pereira, Simone Perna, Hoang Tran Pham, Julian David Pillay, Jalandhar Pradhan, Prem Prakash, Dimas Ria Angga Pribadi, Tina Priscilla, Jagadeesh Puvvula, Raghu Anekal Radhakrishnan, Ibrar Rafique, Fakher Rahim, Amir Masoud Rahmani, Nazanin Rahmanian, Vahid Rahmanian, Sathish Rajaa, Shakthi Kumaran Ramasamy, Mithun Rao, Sowmya J Rao, Mohammad-Mahdi Rashidi, Salman Rawaf, Elrashdy Moustafa Mohamed Redwan, Allen Guy Ross, Pooya Saeedi, Rajesh Sagar, Amirhossein Sahebkar, Soumya Swaroop Sahoo, Zahra Saif, Mirza Rizwan Sajid, Nasir Salam, Afeez Abolarinwa Salami, Mohamed A Saleh, Milena M Santric-Milicevic, Benn Sartorius, Sabyasachi Senapati, Allen Seylani, Pritik A Shah, Samiah Shahid, Masood Ali Shaikh, Mohammad Ali Shamshirgaran, Anas Shamsi, Mohd Shanawaz, Amin

Sharifan, Mahabalesh Shetty, Desalegn Shiferaw, Reza Shirkoohi, Aminu Shittu, Emmanuel Edwar Siddig, Mark J Siedner, Jasvinder A Singh, Robert Sinto, Ranjan Solanki, Shipra Solanki, Lukasz Szarpak, Mohammad Tabish, Yasaman Taheri Abkenar, Jabeen Taiba, JJacques Lukenze Tamuzi, Manoj Tanwar, Elvis Enowbeyang Tarkang, Gebrehiwot Teklay, Enoch Teye-Kwadjo, Rajshree Thapa, Rekha Thapar, Friedrich Thienemann, Marcos Roberto Tovani-Palone, Khai Hoan Tram, Thang Huu Tran, Alexander C Tsai, Munkhtuya Tumurkhuu, Megan Verma, Anh Truc Vo, Magdalene K Walters, Yanzhong Wang, Robert G Weintraub, Gebre Adhanom Weldu, Ronny Westerman, Mesfin Agachew Woldekidan, Zenghonga Wu, Saber Yezli, Arzu Yiğit, Naohiro Yonemoto, Fathiah Zakham, Hanqing Zhao, Peng Zheng, Abzal Zhumagaliuly, Liu Zihao, Alimuddin Zumla, and Samer H Zyoud.

Developing methods or computational machinery

Jeffrey W Imai-Eaton, Spencer A Pease, Magdalene K Walters, Peng Zheng

Providing critical feedback on methods or results

Yohannes Habtegiorgis Abate, Hedayat Abbastabar, Parsa Abdi, Meriem Abdoun, Jeza Muhamad Abdul Aziz, Hassan Abidi, Olumide Abiodun, Richard Gyan Aboagye, Lucas Guimarães Abreu, Yonas Derso Abtew, Eman Abu-Gharbieh, Salahdein Aburuz, Isaac Yeboah Addo, Victor Adekanmbi, Charles Oluwaseun Adetunji, Kishor Adhikari, Qorinah Estiningtyas Sakilah Adnani, Fatemeh Afrashteh, Saira Afzal, Shahin Aghamiri, Feleke Doyore Agide, Williams Agyemang-Duah, Bright Opoku Ahinkorah, Aqeel Ahmad, Sajjad Ahmad, Shahzaib Ahmad, Ayman Ahmed, Haroon Ahmed, Mohammed Ahmed, Safoora Ahmed, Syed Anees Ahmed, Gizachew Taddesse Akalu, Karolina Akinosoglou, Salah Al Awaidy, Hanadi Al Hamad, Amjad S Al Mosa, Omar Ali Mohammed Al Zaabi, Samer O Alalalmeh, Nazmul Alam, Noore Alam, Fahad Mashhour Alanezi, Daniel Shewaye Alayu, Mohammad T AlBataineh, Seyedeh Yasaman Alemohammad, Adel Ali Saeed Al-Gheethi, Abid Ali, Mohammed Usman Ali, Syed Shujait Ali, Waad Ali, Akram Al-Ibraheem, Awais Altaf, Diala Altwalbeh, Nelson Alvis-Guzman, Walid Adnan Al-Zyoud, Reza Amani, Tewodros Getnet Amera, Edward Kwabena Ameyaw, Sohrab Amiri, Ganiyu Adeniyi Amusa, Abhishek Anil, Abdul-Azeez Adeyemi Anjorin, Carl Abelardo T Antonio, Saleha Anwar, Razique Anwer, Ekenedilichukwu Emmanuel Anyabolo, Anayochukwu Edward Anyasodor, Geminn Louis Carace Apostol, Ali Ardekani, er Areda, Brhane Berhe Aregawi, Abdulfatai Aremu, Keivan Armani, Mubarek Yesse Ashemo, Marvellous O Asika, Haftu Asmerom Asmerom, Maha Moh'd Wahbi Atout, Avinash Aujayeb, Hamzeh Awad, Adedapo Wasiu Awotidebe, Beatriz Paulina Ayala Quintanilla, Firayad Ayele, Sina Azadnajafabad, Darshan B B, Giridhara Rathnaiah Babu, Muhammad Badar, Saeed Bahramian, Abdulaziz T Bako, Wondu Feyisa Balcha, Kiran Bam, Biswajit Banik, Mainak Bardhan, Till Winfried Bärnighausen, Hiba Jawdat Barqawi, Zarrin Basharat, Hameed Akande Bashiru, Afisu Basiru, Mohammad-Mahdi Bastan, Saurav Basu, Prapthi Persis Bathini, Kavita Batra, Ravi Batra, Nebiyou Simegnew Bayleyegn, Tahmina Begum, Amir Hossein Behnoush, Maryam Beiranvand, Abel Cherkos Belete, Melaku Ashagrie Belete, Azizullah Beran, Alemshet Yirga Berhie, Amiel Nazer C Bermudez, Robert S Bernstein, Nikha Bhardwaj, Pankaj Bhardwaj, Ajay Nagesh Bhat, Vivek Bhat, Gurjit Kaur Bhatti, Jasvinder Singh Singh Bhatti, Keralem Anteneh Bishaw, Khushboo D Bisht, Trupti Bodhare, Aadam Olalekan Bodunrin, Hamed Borhany, Edmond D Brewer, Colin Stewart Brown, Danilo Buonsenso, Katrin Burkart, Yasser Bustanji, Sam Byrne, Chao Cao, Rosario Cárdenas, Austin Carter, Muthia Cenderadewi, Joshua Chadwick, Chiranjib Chakraborty, Sandip Chakraborty, Rama Mohan Chandika, Vijay Kumar Chattu, Akhilanand Chaurasia, Guangjin Chen, Patrick R Ching, Hitesh Chopra, Dinh-Toi Chu, Isaac Sunday Chukwu, Zinhle Cindi, Haley Comfort, Natalia Cruz-Martins, Silvia Magali Cuadra-Hernández, Omid Dadras, Tukur Dahiru, Xiaochen Dai, Aso Mohammad Darwesh, José das Neves, Nihar Ranjan Dash, Mohsen Dashti, Fernando Pio De la

Hoz, Shayom Debopadhaya, Louisa Degenhardt, Ivan Delgado-Enciso, Kebede Deribe, Don C Des Jarlais, Hardik Dineshbhai Desai, Amol S Dhane, Sameer Dhingra, Daniel Diaz, Michael R Diaz, Delaney D Ding, Thanh Chi Do, Sushil Dohare, Regina-Mae Villanueva Dominguez, Deepa Dongarwar, Wendel Mombaque dos Santos, Ojas Prakashbhai Doshi, Ashel Chelsea Dsouza, Haneil Larson Dsouza, Viola Savy Dsouza, Senbagam Duraisamy, Arkadiusz Marian Dziedzic, Alireza Ebrahimi, Abdelaziz Ed-Dra, Hisham Atan Edinur, Ferry Efendi, Michael Ekholuenetale, Temitope Cyrus Ekundayo, Iman El Sayed, Muhammed Elhadi, Sharareh Eskandarieh, Majid Eslami, Ugochukwu Anthony Eze, Ayesha Fahim, Ali Fatehizadeh, Nelsensius Klau Fauk, Patrick Fazeli, Nuno Ferreira, Florian Fischer, Morenike Oluwatoyin Folayan, Behzad Foroutan, Tahvi D Frank, Takeshi Fukumoto, Sridevi G, Abhay Motiramji Gaidhane, Aravind P Gandhi, Mohammad Arfat Ganiyani, Miglas Welay Gebregergis, Mesfin Gebrehiwot, Teferi Gebru Gebremeskel, Motuma Erena Getachew, Keyghobad Ghadiri, Ahmad Ghashghaee, Ehsan Gholami, Nasim Gholizadeh, Mahsa Ghorbani, Alem Abera Girmay, Mahaveer Golechha, Alessandra C Goulart, Anmol Goyal, Mesay Dechasa Gudeta, Bhawna Gupta, Sapna Gupta, Awoke Derbie Habteyohannes, Dariush Haghmorad, Arvin Haj-Mirzaian, Demelash Woldeyohannes Handiso, Zaim Anan Haq, Harapan Harapan, Arief Hargono, Ahmed I Hasaballah, Md Saquib Hasnain, Shoaib Hassan, Soheil Hassanipour, Simon I Hay, Omar E Hegazi, Mohammad Heidari, Kamal Hezam, Nguyen Quoc Hoan, Praveen Hoogar, Mehdi Hosseinzadeh, Ahmad Hosseinzadeh Adli, Kiavash Hushmandi, Hong-Han Huynh, Segun Emmanuel Ibitoye, Adalia Ikiroma, Kevin S Ikuta, Olayinka Stephen Ilesanmi, Irena M Ilic, Jeffrey W Imai-Eaton, Arnaud Iradukunda, Mustafa Alhaji Isa, Ihoghosa Osamuyi Iyamu, Vinothini J, Kathryn H Jacobsen, Deepa Jahagirdar, Akhil Jain, Ammar Abdulrahman Jairoun, Mihajlo Jakovljevic, Manthan Dilipkumar Janodia, Amirreza Javadi Mamaghani, Mohammad Jokar, Jost B Jonas, Nitin Joseph, Ali Kabir, Md. Awal Kabir, Vidya Kadashetti, Feroze Kaliyadan, Kehinde Kazeem Kanmodi, Suthanthira Kannan S, Ibraheem M Karaye, Arman Karimi Behnagh, Molly B Kassel, Gbenga A Kayode, Himanshu Khajuria, Nauman Khalid, Anees Ahmed Khalil, Faham Khamesipour, Ejaz Ahmad Khan, Gulfaraz Khan, Mohammad Jobair Khan, Yusra H Khan, Feriha Fatima Khidri, Zahra Khorrami, Majid Khosravi, Jong Yeob Kim, Min Seo Kim, Yun Jin Kim, Adnan Kisa, Sezer Kisa, Somayeh Komaki, Shivakumar KM Marulasiddaiah Kondlahalli, Sindhura Lakshmi Koulmane Laxminarayana, Kewal Krishan, Md Abdul Kuddus, Mukhtar Kulimbet, Vishnutheertha Kulkarni, Nithin Kumar, Rakesh Kumar, Vijay Kumar, Hmwe H Kyu, Muhammad Awwal Ladan, Dharmesh Kumar Lal, Dylan Lasher, Thao Thi Thu Le, Seung Won Lee, Kate E LeGrand, Temesgen L Lerango, Ming-Chieh Li, Virendra S Ligade, Stephen S Lim, Liknaw Workie Limenh, Christine Lin, Xuefeng Liu, Rakesh Lodha, Arianna Maever Loreche, Hawraz Ibrahim M. Amin, Jianing Ma, Zheng Feei Ma, Azeem Majeed, Elaheh Malakan Rad, Hardeep Singh Malhotra, Kashish Malhotra, Ahmad Azam Malik, Iram Malik, Tauqeer Hussain Mallhi, Mohammad Ali Mansournia, Bishnu P Marasini, Bernardo Alfonso Martinez-Guerra, Francisco Rogerlândio Rogerlândio Martins-Melo, Miquel Martorell, Navgeet Mathur, Anna Laura W McKowen, Hadush Negash Meles, Endalkachew Belayneh Melese, Ziad Ahmed Memish, Walter Mendoza, Ritesh G Menezes, Tomislav Mestrovic, Peter Meylakhs, Laurette Mhlanga, Ana Carolina Micheletti Gomide Nogueira de Sá, Giuseppe Minervini, Le Huu Nhat Minh, Babak Moazen, Sakineh Mohammad-Alizadeh-Charandabi, Abdollah Mohammadian-Hafshejani, Hussen Mohammed, Mustapha Mohammed, Salahuddin Mohammed, Ali H Mokdad, Lorenzo Monasta, Mohammad Ali Moni, Fateme Montazeri, Maryam Moradi, Yousef Moradi, Rohith Motappa, Vincent Mougin, Emmanuel A Mpolya, George Duke Mukoro, Francesk Mulita, Kavita Munjal, Yanjinlkham Munkhsaikhan, B.V. Murlimanju, Christopher J L Murray, Fungai Musaigwa, Ghulam Mustafa, Saravanan Muthupandian, Ahamarshan Jayaraman Nagarajan, Mohsen Naghavi, Pirouz Naghavi, Gurudatta Naik, Firzan Nainu, Mohammad Sadeq Najafi, Shumaila Nargus, Samidi Nirasha Kumari Navaratna, Muhammad Naveed, Biswa Prakash

Nayak, Vinod C Nayak, Sabina Onyinye Nduaguba, Chernet Tafere Negesse, Mohammad Hadi Nematollahi, Georges Nguefack-Tsague, Dang H Nguyen, Hien Quang Nguyen, Van Thanh Nguyen, Robina Khan Niazi, Yeshambel T Nigatu, Nasrin Nikravangolsefid, Vikram Niranjan, Chukwudi A Nnaji, Syed Toukir Ahmed Noor, Nawsherwan Not applicable, Jean Jacques Noubiap, Amanda Novotney, Chisom Adaobi Nri-Ezedi, Fred Nugen, Jerry John Nutor, Chimezie Igwegbe Nzoputam, Ogochukwu Janet Nzoputam, Kehinde O Obamiro, Ismail A Odetokun, Onome Bright Oghenetega, Ayodipupo Sikiru Oguntade, Sylvester Reuben Okeke, Akinkunmi Paul Okekunle, Osaretin Christabel Okonji, Andrew T Olagunju, Babayemi Oluwaseun Olakunde, Oladotun Victor Olalusi, Matthew Idowu Olatubi, Abdulhakeem Abayomi Olorukooba, Isaac Iyinoluwa Olufadewa, Obinna E Onwujekwe, Abdulahi Opejin, Michal Ordak, Verner N Orish, Edgar Ortiz-Brizuela, Uchechukwu Levi Osuagwu, Amel Ouyahia, Mahesh Padukudru P A, Jagadish Rao Padubidri, Ashok Pandey, Leonidas D Panos, Jose L Paredes, Pragyan Paramita Parija, Romil R Parikh, Ava Pashaei, Maja Pasovic, Aslam Ramjan Pathan, Shankargouda Patil, Veincent Christian Filipino Pepito, Emmanuel K Peprah, Prince Peprah, Marcos Pereira, Simone Perna, Hoang Tran Pham, Julian David Pillay, Ramesh Poluru, Maarten J Postma, Naeimeh Pourtaheri, Jalandhar Pradhan, Prem Prakash, Dimas Ria Angga Pribadi, Tina Priscilla, Jagadeesh Puvvula, Asma Saleem Qazi, Raghu Anekal Radhakrishnan, Quinn Rafferty, Ibrar Rafique, Fakher Rahim, Afarin Rahimi-Movaghar, Vafa Rahimi-Movaghar, Mosiur Rahman, Amir Masoud Rahmani, Shayan Rahmani, Mohammad Rahmanian, Nazanin Rahmanian, Vahid Rahmanian, Sathish Rajaa, Hazem Ramadan, Mahmoud Mohammed Ramadan, Shakthi Kumaran Ramasamy, Pushkal Sinduvadi Ramesh, Kritika Rana, Mithun Rao, Sowmya J Rao, Mohammad-Mahdi Rashidi, Devarajan Rathish, Santosh Kumar Rauniyar, Salman Rawaf, Elrashdy Moustafa Mohamed Redwan, Robert C Reiner Jr., Jefferson Antonio Buendia Rodriguez, Kevin T Root, Allen Guy Ross, Cameron John Sabet, Basema Ahmad Saddik, Mohammad Reza Saeb, Pooya Saeedi, Rajesh Sagar, Fatemeh Saheb Sharif-Askari, Narjes Saheb Sharif-Askari, Soumya Swaroop Sahoo, Zahra Saif, Mirza Rizwan Sajid, Nasir Salam, Afeez Abolarinwa Salami, Mohamed A Saleh, Hossein Samadi Kafil, Abdallah M Samy, Rama Krishna Sanjeev, Milena M Santric-Milicevic, Benn Sartorius, Anudeep Sathyanarayan, Maheswar Satpathy, Austin E Schumacher, Sabyasachi Senapati, Yashendra Sethi, Allen Seylani, Pritik A Shah, Samiah Shahid, Masood Ali Shaikh, Ali Shamekh, Mohammad Ali Shamshirgaran, Anas Shamsi, Mohd Shanawaz, Mohammed Shannawaz, Amin Sharifan, Javad Sharifi-Rad, Shamee Shastry, Rekha Raghuveer Shenoy, Mahabalesh Shetty, Pavanchand H Shetty, Desalegn Shiferaw, Reza Shirkoohi, Aminu Shittu, Sunil Shrestha, Migbar Mekonnen Sibhat, Emmanuel Edwar Siddig, Mark J Siedner, Harmanjit Singh, Jasvinder A Singh, Paramdeep Singh, Surjit Singh, Robert Sinto, Amanda E Smith, Farrukh Sobia, Anton Sokhan, Ranjan Solanki, Shipra Solanki, Sahabi K Sulaiman, Lukasz Szarpak, Sree Sudha T Y, Mohammad Tabish, Santosh Kumar Tadakamadla, Yasaman Taheri Abkenar, Jabeen Taiba, Iman M Talaat, Mircea Tampa, Jacques Lukenze Tamuzi, Manoj Tanwar, Elvis Enowbeyang Tarkang, Nuno Taveira, Gebrehiwot Teklay, Behailu Terefe Tesfaye, Enoch Teye-Kwadjo, Ramna Thakur, Rajshree Thapa, Friedrich Thienemann, Joe Thomas, Khai Hoan Tram, Thang Huu Tran, Alexander C Tsai, Guesh Mebrahtom Tsegay, Munkhtuya Tumurkhuu, Arit Udoh, Atta Ullah, Irfan Ullah, Muhammad Umair, Muhammad Umar, Bhaskaran Unnikrishnan, Asokan Govindaraj Vaithinathan, Shoban Babu Varthya, Megan Verma, Georgios-Ioannis Verras, Jorge Hugo Villafañe, Avina Vongpradith, Theo Vos, Mandaras Tariku Walde, Magdalene K Walters, Yanzhong Wang, Muhammad Waqas, Paul Ward, Robert G Weintraub, Haftom Legese Weldetinsaa, Gebre Adhanom Weldu, Ronny Westerman, Nuwan Darshana Wickramasinghe, Mesfin Agachew Woldekidan, Nigus Kassie Worku, Xinsheng Wu, Zenghong Wu, Gesila Endashaw Yesera, Saber Yezli, Siyan Yi, Arzu Yiğit, Dehui Yin, Yazachew Yismaw, Dong Keon Yon, Naohiro Yonemoto, Fathiah Zakham, Haijun Zhang, Jingya Zhang, Meixin Zhang, Hanqing Zhao,

Peng Zheng, Bin Zhu, Qingyuan Zhuang, Abzal Zhumagaliuly, Magdalena Zielińska, Liu Zihao, Mohammad Zoladl, Alimuddin Zumla, and Samer H Zyoud.

Drafting the work or revising it critically for important intellectual content

Hedayat Abbastabar, Atef Abdelkader, Parsa Abdi, Meriem Abdoun, Olumide Abiodun, Richard Gyan Aboagye, Lucas Guimarães Abreu, Yonas Derso Abtew, Ahmed Abu-Zaid, Isaac Yeboah Addo, Charles Oluwaseun Adetunji, Juliana Bunmi Adetunji, Daniel Adedayo Adeyinka, Kishor Adhikari, Leticia Akua Adzigbli, Fatemeh Afrashteh, Antonella Agodi, Bright Opoku Ahinkorah, Aqeel Ahmad, Sajjad Ahmad, Shahzaib Ahmad, Ali Ahmed, Ayman Ahmed, Ibrar Ahmed, Mohammed Ahmed, Safoora Ahmed, Syed Anees Ahmed, Gizachew Taddesse Akalu, Salah Al Awaidy, Amjad S Al Mosa, Nazmul Alam, Noore Alam, Fahad Mashhour Alanezi, Daniel Shewaye Alayu, Mohammad T AlBataineh, Abid Ali, Liaqat Ali, Mohammed Usman Ali, Waad Ali, Akram Al-Ibraheem, Diala Altwalbeh, Nelson Alvis-Guzman, Walid Adnan Al-Zyoud, Reza Amani, Tewodros Getnet Amera, Sohrab Amiri, Hubert Amu, Abdul-Azeez Adeyemi Anjorin, Carl Abelardo T Antonio, Saleha Anwar, Razique Anwer, Ekenedilichukwu Emmanuel Anyabolo, Anayochukwu Edward Anyasodor, Ali Ardekani, Avinash Aujayeb, Hamzeh Awad, Adedapo Wasiu Awotidebe, Beatriz Paulina Ayala Quintanilla, Firayad Ayele, Shahkaar Aziz, Giridhara Rathnaiah Babu, Muhammad Badar, Saeed Bahramian, Abdulaziz T Bako, Kiran Bam, Biswajit Banik, Mainak Bardhan, Hiba Jawdat Barqawi, Zarrin Basharat, Hameed Akande Bashiru, Afisu Basiru, Mohammad-Mahdi Bastan, Saurav Basu, Kavita Batra, Ravi Batra, Nebiyou Simegnew Bayleyegn, Tahmina Begum, Maryam Beiranvand, Abel Cherkos Belete, Melaku Ashagrie Belete, Apostolos Beloukas, Azizullah Beran, Alemshet Yirga Berhie, Amiel Nazer C Bermudez, Robert S Bernstein, Nikha Bhardwaj, Pankaj Bhardwaj, Ajay Nagesh Bhat, Vivek Bhat, Trupti Bodhare, Souad Bouaoud, Edmond D Brewer, Colin Stewart Brown, Katrin Burkart, Yasser Bustanji, Austin Carter, Muthia Cenderadewi, Chiranjib Chakraborty, Patrick R Ching, Sonali Gajanan Choudhari, Eric Chung, Zinhle Cindi, Haley Comfort, Silvia Magali Cuadra-Hernández, Bashir Dabo, Omid Dadras, Gizachew Worku Dagnew, Xiaochen Dai, Aso Mohammad Darwesh, José das Neves, Nihar Ranjan Dash, Mohsen Dashti, Shayom Debopadhaya, Ivan Delgado-Enciso, Don C Des Jarlais, Hardik Dineshbhai Desai, Keshab Deuba, Amol S Dhane, Sameer Dhingra, Daniel Diaz, Michael R Diaz, Sushil Dohare, Deepa Dongarwar, Ashel Chelsea Dsouza, Viola Savy Dsouza, Senbagam Duraisamy, Arkadiusz Marian Dziedzic, Alireza Ebrahimi, Abdelaziz Ed-Dra, Hisham Atan Edinur, Ferry Efendi, Michael Ekholuenetale, Iman El Sayed, Muhammed Elhadi, Chadi Eltaha, Sharareh Eskandarieh, Ugochukwu Anthony Eze, Ali Fatehizadeh, Patrick Fazeli, Ginenus Fekadu, Nuno Ferreira, Belete Sewasew Firew, Florian Fischer, Morenike Oluwatoyin Folayan, Behzad Foroutan, Tahvi D Frank, Takeshi Fukumoto, Sridevi G, Muktar A Gadanya, Abhay Motiramji Gaidhane, Abduzhappar Gaipov, Aravind P Gandhi, Mohammad Arfat Ganiyani, Miglas Welay Gebregergis, Mesfin Gebrehiwot, Keyghobad Ghadiri, Afsaneh Ghasemzadeh, Ahmad Ghashghaee, Ehsan Gholami, Nasim Gholizadeh, Mahsa Ghorbani, Artyom Urievich Gil, Mahaveer Golechha, Alessandra C Goulart, Mesay Dechasa Gudeta, Bhawna Gupta, Sapna Gupta, Awoke Derbie Habteyohannes, Arvin Haj-Mirzaian, Rabih Halwani, Demelash Woldeyohannes Handiso, Harapan Harapan, Arief Hargono, Ahmed I Hasaballah, Md Saquib Hasnain, Shoaib Hassan, Soheil Hassanipour, Simon I Hay, Jiawei He, Omar E Hegazi, Mbuzeleni Mbuzeleni Hlongwa, Praveen Hoogar, Mehdi Hosseinzadeh, Ahmad Hosseinzadeh Adli, Tsegaye Gebreyes Hundie, Kiavash Hushmandi, Segun Emmanuel Ibitoye, Adalia Ikiroma, Kevin S Ikuta, Irena M Ilic, Jeffrey W Imai-Eaton, Mustafa Alhaji Isa, Nahlah Elkudssiah Ismail, Vinothini J, Kathryn H Jacobsen, Akhil Jain, Ammar Abdulrahman Jairoun, Mihajlo Jakovljevic, Amirreza Javadi Mamaghani, Alelign Tasew Jema, Mohammad Jokar, Charity Ehimwenma Joshua, Md. Awal Kabir, Zubair Kabir, Vidya Kadashetti, Feroze Kaliyadan, Suthanthira Kannan S, Ibraheem M Karaye, Arman Karimi Behnagh, Gbenga A Kayode,

Himanshu Khajuria, Nauman Khalid, Anees Ahmed Khalil, Faham Khamesipour, Ejaz Ahmad Khan, Gulfaraz Khan, M Nuruzzaman Khan, Yusra H Khan, Zahra Khorrami, Majid Khosravi, Jagdish Khubchandani, Jong Yeob Kim, Min Seo Kim, Yun Jin Kim, Somayeh Komaki, Shivakumar KM Marulasiddaiah Kondlahalli, Kewal Krishan, Barthelemy Kuate Defo, Md Abdul Kuddus, Mukhtar Kulimbet, Vishnutheertha Kulkarni, Manasi Kumar, Nithin Kumar, Rakesh Kumar, Vijay Kumar, Hmwe H Kyu, Dharmesh Kumar Lal, Dylan Lasher, Nhi Huu Hanh Le, Thao Thi Thu Le, Kate E LeGrand, Ming-Chieh Li, Virendra S Ligade, Stephen S Lim, Hawraz Ibrahim M. Amin, Jianing Ma, Zheng Feei Ma, Azeem Majeed, Elaheh Malakan Rad, Iram Malik, Mohammad Ali Mansournia, Bishnu P Marasini, Bernardo Alfonso Martinez-Guerra, Miquel Martorell, Roy Rillera Marzo, Navgeet Mathur, Hadush Negash Meles, Endalkachew Belayneh Melese, Ziad Ahmed Memish, Walter Mendoza, Ritesh G Menezes, Tuomo J Meretoja, Tomislav Mestrovic, Peter Meylakhs, Irmina Maria Michalek, Giuseppe Minervini, Babak Moazen, Nouh Saad Mohamed, Abdollah Mohammadian-Hafshejani, Hussen Mohammed, Salahuddin Mohammed, Ali H Mokdad, Fateme Montazeri, Maryam Moradi, Yousef Moradi, Sumaira Mubarik, George Duke Mukoro, Kavita Munjal, B.V. Murlimanju, Christopher J L Murray, Fungai Musaigwa, Ghulam Mustafa, Saravanan Muthupandian, Mohsen Naghavi, Pirouz Naghavi, Gurudatta Naik, Firzan Nainu, Mohammad Sadeq Najafi, Muhammad Naveed, Georges Nguefack-Tsague, Hien Quang Nguyen, Van Thanh Nguyen, Robina Khan Niazi, Nasrin Nikravangolsefid, Chukwudi A Nnaji, Nawsherwan Not applicable, Jean Jacques Noubiap, Amanda Novotney, Chisom Adaobi Nri-Ezedi, Fred Nugen, Jerry John Nutor, Chimezie Igwegbe Nzoputam, Ogochukwu Janet Nzoputam, Ismail A Odetokun, Onome Bright Oghenetega, Ayodipupo Sikiru Oguntade, Sylvester Reuben Okeke, Andrew T Olagunju, Oladotun Victor Olalusi, Abdulhakeem Abayomi Olorukooba, Ahmed Omar Bali, Abdulahi Opejin, Michal Ordak, Verner N Orish, Edgar Ortiz-Brizuela, Uchechukwu Levi Osuagwu, Mahesh Padukudru P A, Jagadish Rao Padubidri, Claudia Palladino, Leonidas D Panos, Pragyan Paramita Parija, Romil R Parikh, Ava Pashaei, Maja Pasovic, Sangram Kishor Patel, Aslam Ramjan Pathan, Shankargouda Patil, Shrikant Pawar, Spencer A Pease, Veincent Christian Filipino Pepito, Prince Peprah, Marcos Pereira, Simone Perna, Ionela-Roxana Petcu, Hoang Tran Pham, Julian David Pillay, Maarten J Postma, Prem Prakash, Thejeswar N N Prakasham, Dimas Ria Angga Pribadi, Jagadeesh Puvvula, Ibrahim Qattea, Asma Saleem Qazi, Raghu Anekal Radhakrishnan, Quinn Rafferty, Ibrar Rafique, Mosiur Rahman, Amir Masoud Rahmani, Shayan Rahmani, Mohammad Rahmanian, Nazanin Rahmanian, Sathish Rajaa, Shakthi Kumaran Ramasamy, Kritika Rana, Chhabi Lal Ranabhat, Mithun Rao, Sowmya J Rao, Mohammad-Mahdi Rashidi, Devarajan Rathish, Santosh Kumar Rauniyar, Salman Rawaf, Elrashdy Moustafa Mohamed Redwan, Mohsen Rezaeian, Jefferson Antonio Buendia Rodriguez, Kevin T Root, Kunle Rotimi, Nitai Roy, Godfrey M Rwegerera, Basema Ahmad Saddik, Umar Saeed, Pooya Saeedi, Sher Zaman Zaman Safi, Rajesh Sagar, Fatemeh Saheb Sharif-Askari, Narjes Saheb Sharif-Askari, Amirhossein Sahebkar, Soumya Swaroop Sahoo, Zahra Saif, Mirza Rizwan Sajid, Nasir Salam, Mohamed A Saleh, Leili Salehi, Hossein Samadi Kafil, Aswini Saravanan, Benn Sartorius, Monika Sawhney, Austin E Schumacher, Mansour Sedighi, Birhan Ewunu Semagn, Yashendra Sethi, Allen Seylani, Pritik A Shah, Samiah Shahid, Masood Ali Shaikh, Mohammed Shannawaz, Amin Sharifan, Javad Sharifi-Rad, Shamee Shastry, Mahabalesh Shetty, Pavanchand H Shetty, Premalatha K Shetty, Desalegn Shiferaw, Aminu Shittu, Sunil Shrestha, Emmanuel Edwar Siddig, Mark J Siedner, Harmanjit Singh, Robert Sinto, Anna Aleksandrovna Skryabina, Ranjan Solanki, Shipra Solanki, Reed J D Sorensen, Lukasz Szarpak, Sree Sudha T Y, Mohammad Tabish, Yasaman Taheri Abkenar, Jabeen Taiba, Mircea Tampa, Jacques Lukenze Tamuzi, Ker-Kan Tan, Manoj Tanwar, Elvis Enowbeyang Tarkang, Enoch Teye-Kwadjo, Ramna Thakur, Pugazhenthan Thangaraju, Rajshree Thapa, Rekha Thapar, Friedrich Thienemann, Khai Hoan Tram, Mai Thi Ngoc Tran, Alexander C Tsai, Arit Udoh,

Atta Ullah, Irfan Ullah, Muhammad Umar, Bhaskaran Unnikrishnan, Sanaz Vahdati, Shoban Babu Varthya, Tommi Juhani Vasankari, Megan Verma, Georgios-Ioannis Verras, Jorge Hugo Villafañe, Anh Truc Vo, Avina Vongpradith, Theo Vos, Magdalene K Walters, Richard G Wamai, Yanzhong Wang, Muhammad Waqas, Gizachew Tadesse Wassie, Ronny Westerman, Nuwan Darshana Wickramasinghe, Mesfin Agachew Woldekidan, Yen Jun Wong, Xinsheng Wu, Sajad Yaghoubi, Gesila Endashaw Yesera, Saber Yezli, Siyan Yi, Dehui Yin, Naohiro Yonemoto, Fathiah Zakham, Jingya Zhang, Peng Zheng, Magdalena Zielińska, Liu Zihao, Yossef Teshome Zikarg, Alimuddin Zumla, and Samer H Zyoud.

Managing the estimation or publications process

Simon I Hay, Hmwe H Kyu, Christopher J L Murray, Amanda Novotney, and Magdalene K Walters
